# Nrg4 Secreted by Brown Adipose Tissue Suppresses Ferroptosis of Sepsis-Induced Liver Injury

**DOI:** 10.1007/s10753-024-02230-z

**Published:** 2025-02-17

**Authors:** Linqi Feng, Jun Cui, Wenlong Chen, Lei Zhu, Panpan Li, Haitao Zhou, Yang Sun, Wei Yi

**Affiliations:** 1 Department of Cardiovascular Surgery, Xijing Hospital, The Fourth Military Medical University, Xi’an, China; 2 Department of Geriatrics, Xijing Hospital, The Fourth Military Medical University, Xi’an, China

**Keywords:** Brown adipose tissue, Sepsis, Liver injury, Nrg4, Ferroptosis

## Abstract

Sepsis is a leading cause of death, with the liver being particularly vulnerable to sepsis-related injuries. This damage significantly contributes to disease progression, underscoring the need for new treatments. Brown adipose tissue (BAT) secretes various cytokines, including neuregulin 4 (Nrg4), which plays a protective role in hepatic glucose and lipid metabolism. Ferroptosis, a key type of cell death in sepsis-induced liver injury, has recently gained attention. This study aimed to investigate how BAT-secreted cytokines alleviate liver ferroptosis in sepsis. Septic liver injury was induced in the control and BAT group using cecal ligation and puncture (CLP) and lipopolysaccharide injections. BAT removal worsened ferroptosis; in contrast, CL316243 activation reduced it. These findings suggest that Nrg4 secretion following BAT activation protects the liver during sepsis by inhibiting ferroptosis. Future therapies targeting BAT activation and Nrg4 could potentially mitigate sepsis-induced liver damage, offering new insights into treatment strategies.

**Supplementary Information:**

The online version contains supplementary material available at 10.1007/s10753-024-02230-z.

## Introduction

Sepsis, a life-threatening syndrome arising from infection, disrupts the intricate balance of the host's immune system, ultimately precipitating organ dysfunction [1]. It often accompanied by multiple organ failure, which contributes to high morbidity and mortality [2]. Owing to its unique anatomical and physiological properties, the liver acts as a key barrier in the immune system, playing a crucial role in immune surveillance and defense against pathogens. It also regulates synthesis of acute-phase proteins and cytokines, adapts metabolism to inflammation, and modulates the body's immune response [3].Liver dysfunction frequently arises in the initial phases of sepsis and is intimately tied to poor patient outcomes. [4]. Sepsis-induced liver injury can impair the clearance of bacteria and lipopolysaccharides (LPS), leading to dysfunction of distant organs and promoting the release of inflammatory cytokines, which may cause secondary cholangitis, cirrhosis, and further organ damage [5]. Despite extensive research, no effective treatment for septic liver injury has been established, and the molecular mechanism remains unclear.

Sepsis is marked by overactivation of the sympathetic nervous system, along with widespread adrenergic stimulation [6]. This condition can stimulate the browning process of white adipose tissue (WAT) in mice [7, 8]. Due to the sympathetic nervous system enhances brown adipose tissue (BAT) activity [9], the activation and functionality of BAT are intimately associated with the browning process of WAT. [10]. BAT has gained considerable attention in metabolism and endocrinology research. BAT serves as a heat-producing thermogenic tissue that contributes to the regulation of core body temperature via non-shivering thermogenesis. Recent studies have confirmed the existence and metabolic activity of BAT in adults [11–13]. Initially, BAT was discovered for its role in treating obesity and type 2 diabetes. BAT can be activated by transplantation [14, 15], cold exposure [16, 17], and specific activators [18, 19] leading to the release of "batokines,” such as protein and lipid factors. These molecules improve glucose and fatty acid metabolism systemically. BAT also influences liver glucose and lipid metabolism by secreting FGF21 and PLTP [20–22]. Additionally, BAT has been shown to have anti-inflammatory effects on the liver by secreting maresin 2 (MaR2) [23]; however, stress-induced BAT secretion of IL-6 can exacerbate LPS-induced terminal liver injury [24]. These findings reveal the explicit crosstalk between BAT and the liver, suggesting that BAT-secreted cytokines impact liver function. However, it remains unclear whether such interactions influence septic liver injury, and the specific batokines involved in liver injury during sepsis have yet to be identified.

In our study, we investigated the relationship between BAT and septic liver injury through BAT ectomy and activation with CL316243. Using two established sepsis models in mice (cecal ligation and puncture [CLP] and LPS intraperitoneal injection), we explored the crosstalk between BAT and the liver during sepsis. We examined the protective role of batokines secreted by BAT on septic liver injury and their potential therapeutic implications for treating liver injury in sepsis.

## Results

### BAT Ectomy in Mice Exacerbates Septic Liver Injury After CLP

**Fig. 1:**
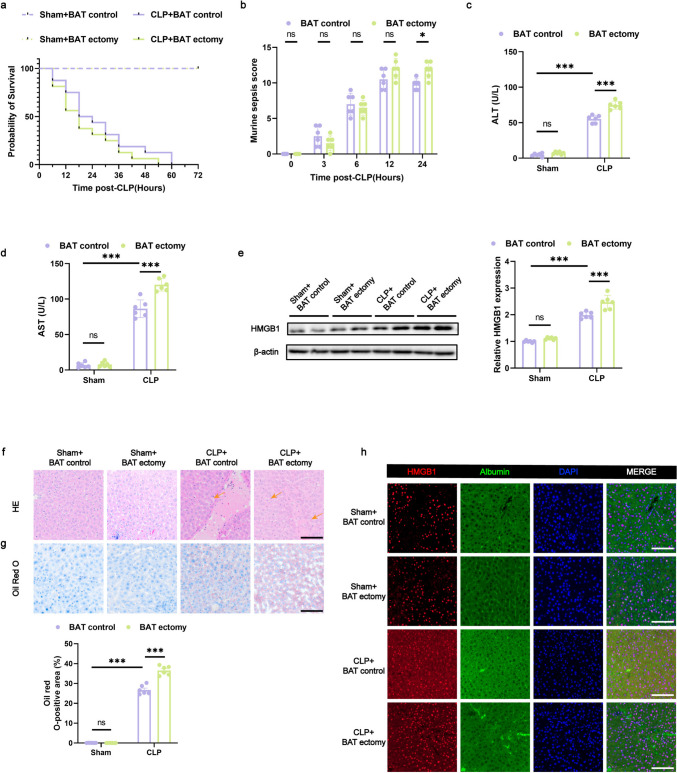
BAT ectomy in mice exacerbates septic liver injury after CLP (**a**) Survival curve of mice within 24 h post-CLP. *n* = 16 per group. **b** Murine sepsis scores at 0, 3, 6, 12, and 24 h post-CLP. *n* = 6 per group. **c**, **d** Serum ALT and AST levels 24 h post-CLP. *n* = 6 per group. **e** Protein expression of HMGB1 in liver tissue 24 h post-CLP. *n* = 6 per group. **f** Histological changes in liver tissue observed via HE staining (the arrows indicate infiltration of inflammatory cells), scale bar: 100 μm. **g** Oil Red O staining of liver sections highlighting lipid droplets accumulation, scale bar: 100 μm. *n* = 6 per group. **h** Representative confocal microscopy images of liver sections stained for HMGB1 (red), albumin (green), and nuclei (blue); scale bar: 100 μm. Data are presented as mean ± SEM. * *p* < 0.05; ** *p* < 0.01; *** *p* < 0.001; ns, not significant. CLP, cecal ligation and puncture; SEM, standard error of the mean

The characteristics of sepsis include overactivation of the sympathetic nervous system and sustained adrenergic stimulation throughout the body [6], both of which are closely linked to BAT activity [9]. Previous studies have demonstrated that BAT secretes multiple factors, including beta-blockers, which play protective roles in liver glucose and lipid metabolism and reduce inflammation. Based on this, we hypothesized that BAT exerts a safeguarding effect against liver injury following sepsis. To test this, CLP or sham surgery was performed in mice to determine whether BAT is involved in liver protection during sepsis. Before undergoing either the sham procedure or CLP, mice were randomly assigned to undergo BAT ectomy or not. As expected, compared with control mice that were subjected to sham surgical procedure, CLP-induced sepsis mice showed a trend of decreased survival rate (Fig. 1a). The murine sepsis score (MSS) [25], which assesses sepsis severity based on behavior, responsiveness, respiration, and appearance, was assessed at 0, 3, 6, 12, and 24 h post-CLP. The MSS confirmed the successful induction of sepsis in the CLP model (Fig. 1b). Significant increases in plasma alanine aminotransferase (ALT) and aspartate aminotransferase (AST) levels confirmed liver damage following CLP (Fig. 1c and d). High mobility group box 1 (HMGB1) assumes a crucial function in liver inflammation and cellular damage during bacterial sepsis. [26, 27]. Western blotting (WB) and immunofluorescence analysis of HMGB1 in CLP mice confirmed liver injury following sepsis (Fig. 1e and h). Hematoxylin and eosin (HE) staining of tissues from CLP mice revealed increased inflammatory cell infiltration; in contrast, Oil Red O staining indicated fat deposition, both of which are hallmarks of sepsis-induced liver damage (Fig. 1f and g).

Interestingly, compared with CLP mice that underwent sham surgery, BAT ectomy led to a further decreased survival rate (Fig. 1a), increased MSS and decreased body temperature (Fig. S1a), indicating more severe sepsis (Fig. 1b). Liver function was significantly worsened, as evidenced by the elevation of plasma ALT and AST levels (Fig. 1c and d), enhanced HMGB1 expression (Fig. 1e and h), intensified inflammatory infiltration (Fig. 1f), and augmented fat accumulation (Fig. 1g). HMGB1 expression (Fig. 1e and h), intensified inflammatory infiltration (Fig. 1f), and augmented fat deposition (Fig. 1g). These findings confirm that BAT has a protective impact on sepsis-induced liver injury, and its removal exacerbates this condition.

### BAT Ectomy in Mice Exacerbates Septic Liver Injury After LPS Administration

**Fig. 2:**
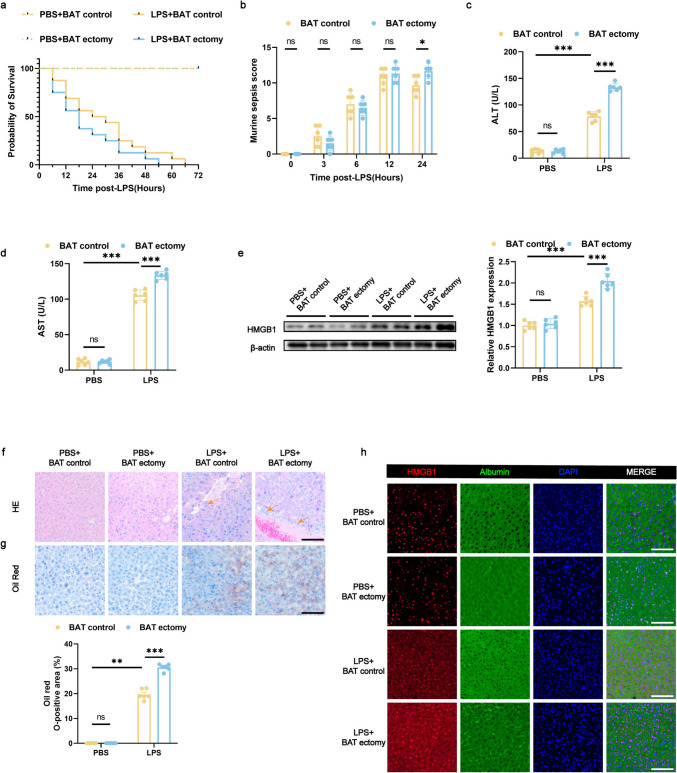
BAT ectomy in mice exacerbates septic liver injury after LPS administration (**a**) Survival curve of mice within 24 h after LPS administration. *n* = 16 per group. **b** Murine sepsis scores at specified intervals (0, 3, 6, 12, and 24 h post-LPS administration). *n* = 6 per group. **c**, **d** Serum concentrations of ALT and AST 24 h post LPS administration. *n* = 6 per group. **e** Protein expression of HMGB1 in liver tissue 24 h after LPS administration. *n* = 6 per group. **f** Histological changes in liver tissue shown by HE staining (the arrows indicate infiltration of inflammatory cells), scale bar: 100 μm. **g** Oil Red O staining of liver sections, highlighting lipid droplets accumulation in the liver tissue, scale bar: 100 μm. *n* = 6 per group. **h** Representative confocal microscopy images of liver sections stained for HMGB1 (red), albumin (green), and nuclei (blue); scale bar: 100 μm. Data are expressed as mean ± SEM. * *p* < 0.05; ** *p* < 0.01; *** *p* < 0.001; ns, not significant. LPS, lipopolysaccharide;; HE, hematoxylin and eosin

We aimed to establish a sepsis model by administering an intraperitoneal injection of LPS or PBS in mice. To investigate whether BAT plays a role in liver protection during sepsis, mice injected with LPS or PBS were randomly subjected to BAT ectomy 72 h prior to sepsis induction. As expected, compared with control mice injected with PBS, the mice suffering from LPS-induced sepsis experienced a decreasing trend in their survival rate. (Fig. 2a). The MSS confirmed the development of sepsis after LPS administration in the mice (Fig. 2b). After undergoing LPS administration, there was a marked elevation in the plasma concentrations of ALT and AST. WB, immunofluorescence, HE staining, and Oil Red O staining for HMGB1 in liver tissue revealed liver injury after sepsis (Fig. 2c–h).

In this study, BAT ectomy was employed to examine its effect on LPS-induced liver injury. Compared with control mice exposed to LPS, those that underwent BAT ectomy prior to LPS treatment exhibited a low survival rate (Fig. 2a), a significant increase in MSS and decreased body temperature (Fig. S1b), indicating more severe sepsis symptoms (Fig. 2b), and elevated serum levels of AST and ALT (Fig. 2c and d). Histological analysis showed reduced inflammatory cell infiltration and increased fat deposition (Fig. 2f and g), supporting the conclusion that BAT ectomy worsens LPS-induced septic liver injury (Fig. 2e and h). Additionally, WB and immunofluorescence results revealed that BAT ectomy increased the protein expression of HMGB1 in liver tissue after LPS administration (Fig. 2e and h). These findings confirm that BAT serves a crucial protective function against sepsis-induced liver injury, and its removal exacerbates liver damage caused by LPS-induced sepsis.

### BAT Activation in Mice Limits Septic Liver Injury After CLP

Given the exacerbation of CLP- and LPS-induced septic liver injury following BAT ectomy, our hypothesis was confirmed: BAT exerts a protective effect against septic liver injury. Since activated BAT secretes adipokines that modulate liver inflammation, and sepsis is associated with inflammatory damage to multiple organs, we propose that activated BAT may help mitigate liver damage during sepsis. One well-established method for activating BAT is through CL316243, a recognized β3-adrenergic receptor activator, which enhances BAT thermogenesis and stimulates the secretion of various batokines [23].

**Fig. 3:**
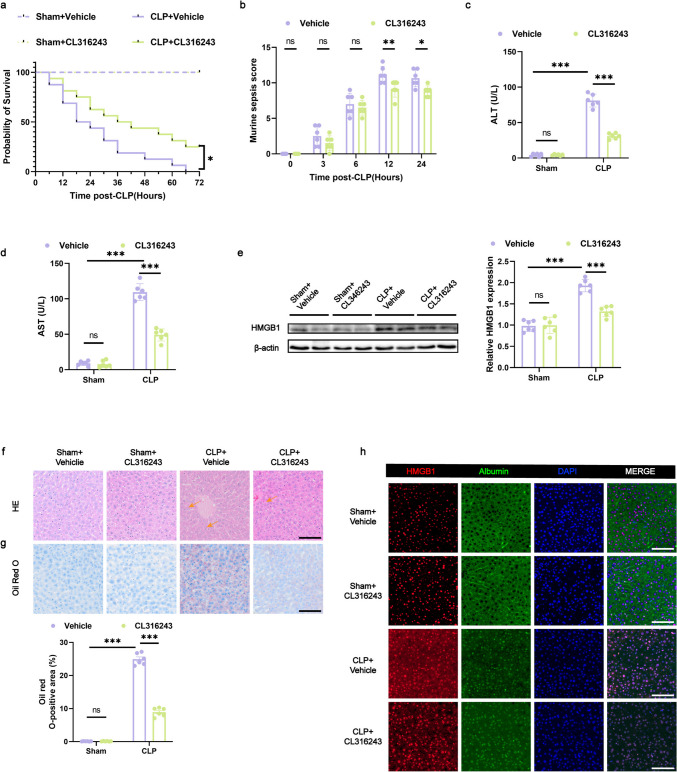
BAT activation in mice limits septic liver injury after CLP (**a**) Survival curve of mice within 24 h post-CLP. *n* = 16 per group. **b** Murine sepsis scores at specified intervals (0, 3, 6, 12, and 24 h post-CLP). *n* = 6 per group. **c**, **d** Serum ALT and AST levels 24 h post-CLP. *n* = 6 per group. **e** Protein expression of HMGB1 in liver tissue 24 h post-CLP. *n* = 6 per group. **f** Histological changes in liver tissue observed via HE staining (the arrows indicate infiltration of inflammatory cells), scale bar: 100 μm. **g** Oil Red O staining of liver sections highlighting lipid droplet accumulation, scale bar: 100 μm. *n* = 6 per group. **h** Representative confocal microscopy of liver sections stained for HMGB1 (red), albumin (green), and nuclei (blue); scale bar: 100 μm. Data are presented as mean ± SEM. * *p* < 0.05; ** *p* < 0.01; *** *p* < 0.001; ns, not significant. CLP, cecal ligation and puncture; HE, hematoxylin and eosin

To assess whether activated BAT provides liver protection during sepsis, mice were intraperitoneally injected with either vehicle or CL316243 for 7 consecutive days, followed by sham surgery to induce a CLP sepsis model. Notably, compared with vehicle-treated mice, those that received CL316243 showed an increased survival rate following CLP-induced sepsis (Fig. 3a). Additionally, a decrease in MSS indicated a reduction in sepsis severity (Fig. 3b). However, there was no difference in body temperature at 24 h after CLP (Fig. S1c). Plasma levels of ALT and AST were significantly reduced, suggesting improved liver function (Fig. 3c and d). Furthermore, WB and immunofluorescence analysis revealed lower levels of HMGB1 in the liver, indicating reduced liver injury (Fig. 3e and h). Histological analyses, including HE staining for inflammatory infiltration and Oil Red O staining for fat deposition, showed that CL316243 significantly limited liver damage during sepsis (Fig. 3f and g). These findings confirm that BAT activation markedly alleviates liver damage in CLP-induced sepsis.

### BAT Activation in Mice Limits Septic Liver Injury After LPS Administration

Based on the significant aggravation of LPS-induced liver injury following BAT ectomy, we aimed to determine whether activated BAT exerts a protective influence on the liver subsequent to LPS-induced sepsis. An LPS-induced sepsis mouse model was developed through intraperitoneal administration of either vehicle or CL316243 for 7 consecutive days, followed by an intraperitoneal injection of LPS or PBS.

**Fig. 4:**
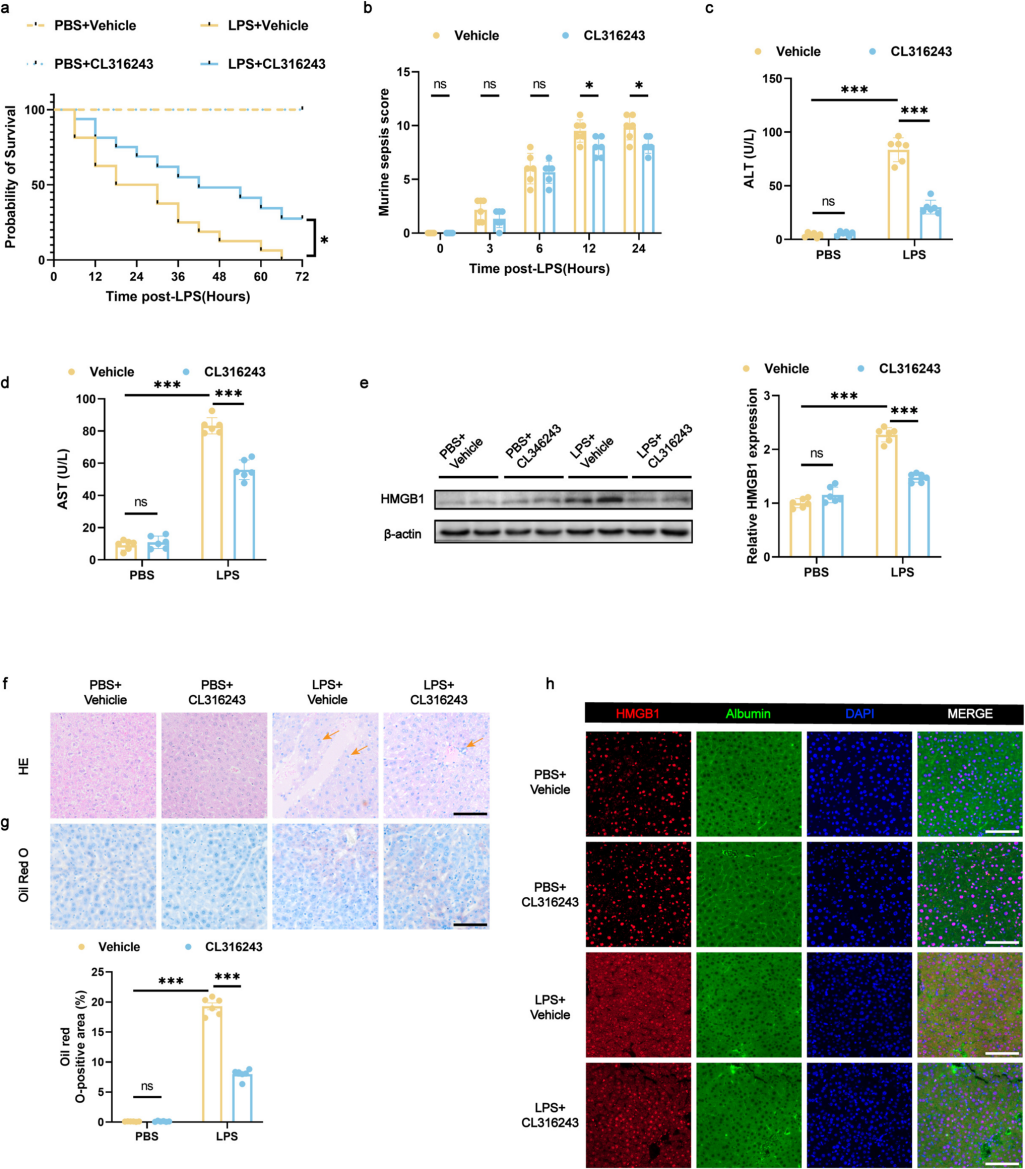
BAT activation in mice limits septic liver injury after LPS administration (**a**) Survival curve of mice within 24 h post-LPS administration. *n* = 16 per group. **b** Murine sepsis scores at specified intervals (0, 3, 6, 12, and 24 h post-LPS administration). *n* = 6 per group. **c**, **d** Serum ALT and AST concentrations 24 h post-LPS. *n* = 6 per group. **e** Protein expression of HMGB1 in liver tissue 24 h post-LPS administration. *n* = 6 per group. **f** Histological changes in liver tissue observed via HE staining (the arrows indicate infiltration of inflammatory cells), scale bar: 100 μm. **g** Representative Oil Red O-stained liver sections from mice, highlighting lipid droplet accumulation in liver tissue, scale bar: 100 μm. *n* = 6 per group. **h** Representative confocal microscopy images of liver sections stained for HMGB1 (red), albumin (green), and nuclei (blue); scale bar: 100 μm. Data are presented as mean ± SEM. * *p* < 0.05; ** *p* < 0.01; *** *p* < 0.001; ns, not significant. LPS, lipopolysaccharide; HE, hematoxylin and eosin

Furthermore, we sought to demonstrate that CL316243 could alleviate liver injury caused by LPS-induced sepsis. Consistent with our hypothesis, mice receiving CL316243 displayed a notably enhanced survival rate, in contrast to those that received the vehicle control after LPS administration (Fig. 4a), the MSS was significantly decreased (Fig. 4b) and the body temperature was increased (Fig. S1d). These findings indicate that both mortality and sepsis symptoms were reduced. Additionally, plasma ALT and AST levels were significantly decreased (Fig. 4c and d), and inflammatory infiltration (Fig. 4f) and fat deposition (Fig. 4g) were reduced, suggesting substantial improvement in liver function. As a molecular marker of liver injury severity during sepsis, HMGB1 Levels were assessed. These results confirm that CL316243 significantly mitigated liver injury during sepsis and indicate that BAT activation is essential in alleviating liver injury caused by sepsis after LPS administration.

### Nrg4 as a Potential BAT Adipokine for Septic Liver Injury

As previously mentioned, BAT plays a protective role in mitigating liver injury during sepsis. We hypothesize that certain factors secreted by BAT may contribute to this protective effect. From a review of the literature on batokines and recent studies, Nrg4, known for its liver-protective properties, emerged as a potential candidate. Based on this, we propose that Nrg4 may be a key factor through which BAT exerts its protective effects on the liver during sepsis.

**Fig. 5:**
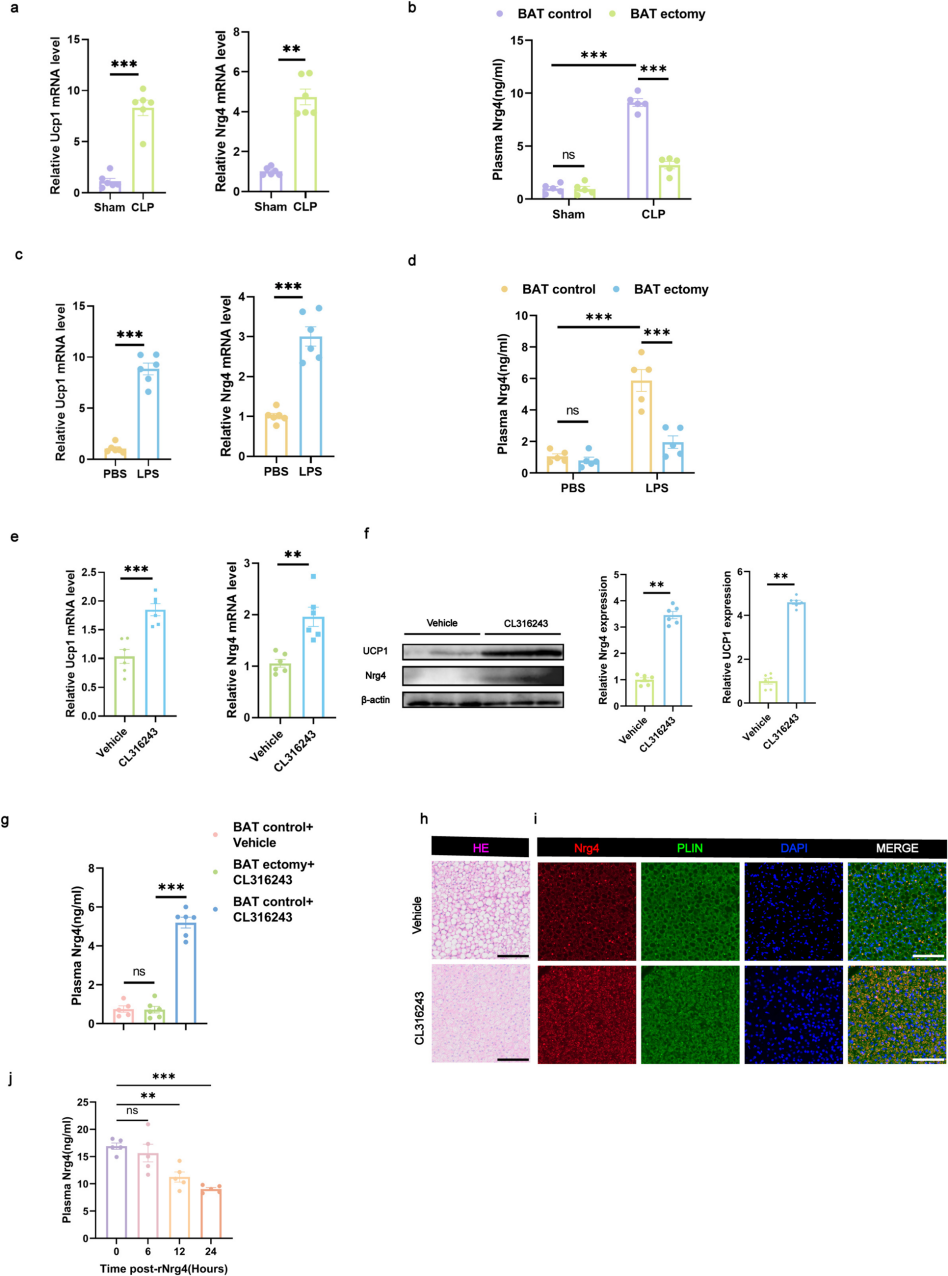
Nrg4 as a potential BAT adipokine for septic liver injury (**a**, **c**, **e**) qRT-PCR analysis quantifying Ucp1 and Nrg4 mRNA levels. *n* = 6 per group. **b**, **d** Serum concentrations of Nrg4. *n* = 6 per group. **f** Protein expression of UCP1 and Nrg4 in BAT. *n* = 6 per group. **g** Serum concentrations of Nrg4. *n* = 5–6. **h** Histological changes in BAT observed via HE staining, scale bar: 100 μm. **i** Representative confocal microscopy images of BAT sections stained for Nrg4 (red), PLIN (green), and nuclei (blue); scale bar: 100 μm. **j** Serum concentrations of Nrg4 at specified intervals (0, 6, 12, and 24 h post-rNrg4 administration). *n* = 5 per group. Data are presented as mean ± SEM. * *p* < 0.05; ** *p* < 0.01; *** *p* < 0.001; ns, not significant. qRT-PCR, quantitative real-time polymerase chain reaction; HE, hematoxylin and eosin; BAT, brown adipose tissue; PLIN, perilipin

In CLP-treated mice, both uncoupling protein 1 (Ucp1) and Nrg4 mRNA expression levels in BAT were significantly elevated compared with sham-operated controls (Fig. 5a). Additionally, plasma levels of Nrg4 were significantly increased (Fig. 5b). Similarly, LPS-treated mice showed a significant rise in Ucp1 and Nrg4 mRNA expression in BAT and higher plasma concentrations of Nrg4 compared with PBS-treated controls (Fig. 5c and d). These results suggest that sepsis stimulates UCP1-dependent thermogenesis and induces the secretion of Nrg4 from BAT into the plasma.

We further explored whether BAT activation via CL316243 mimics the activation seen during sepsis. In comparison to the vehicle group, mice administered with CL316243 demonstrated markedly elevated levels of Ucp1 and Nrg4 mRNA and protein expression in BAT. (Fig. 5e and f). HE staining revealed a significant reduction in lipid droplets within BAT, indicating thermogenic activation (Fig. 5h). Additionally, immunofluorescence staining demonstrated a significant increase in Nrg4 secretion (Fig. 5i), along with elevated plasma Nrg4 levels (Fig. 5g).

These findings collectively demonstrate that CL316243 activates BAT, promoting the secretion of Nrg4 into the bloodstream. In summary, sepsis induces both BAT thermogenesis and Nrg4 secretion; however, CL316243 further enhances Nrg4 release from BAT. These results align with previously reported studies.

### Exogenous Nrg4 Limits Septic Liver Injury After CLP

**Fig. 6:**
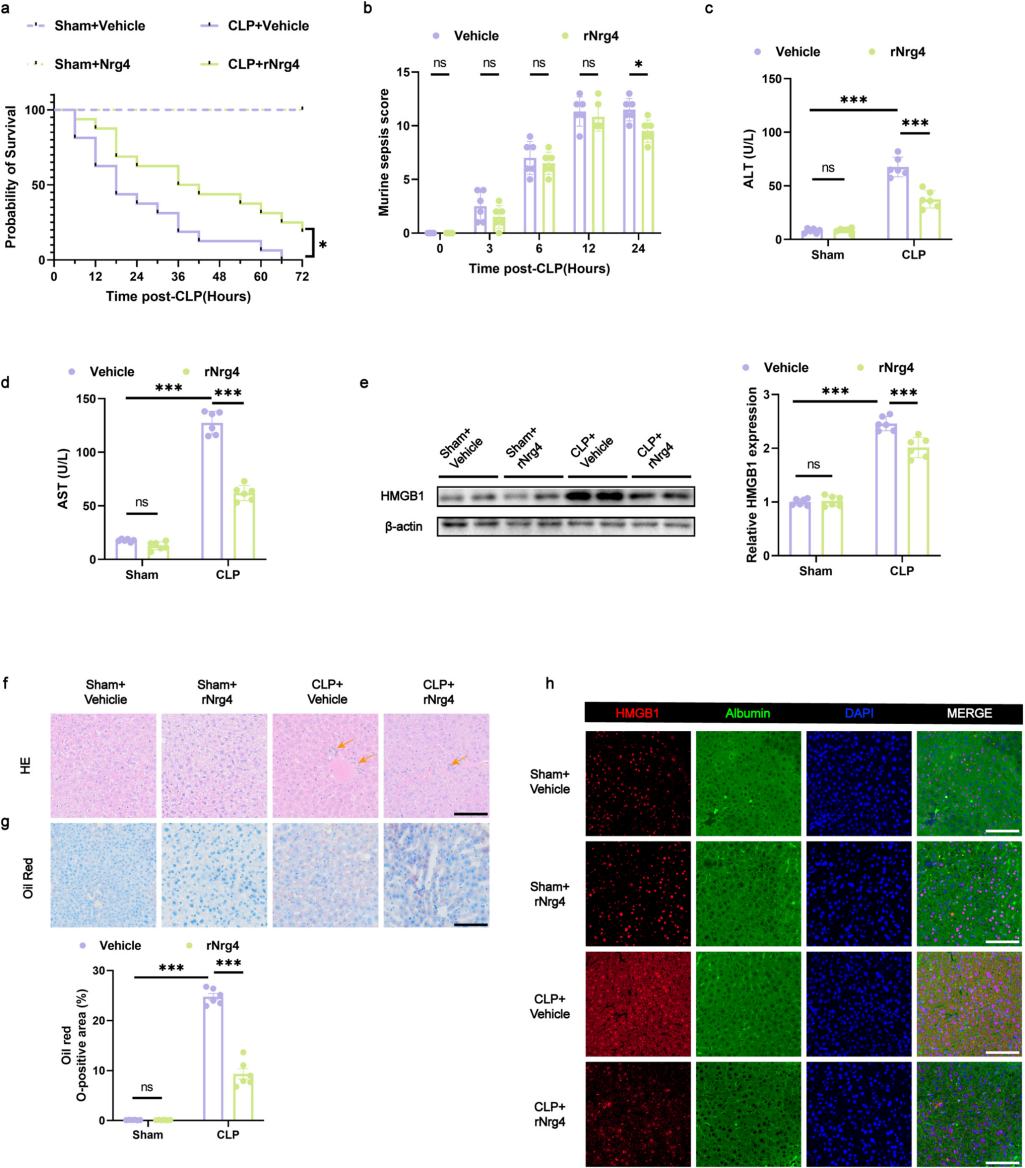
Exogenous Nrg4 limits septic liver injury after CLP (**a**) Survival curve of mice within 24 h post-CLP. *n* = 16 per group. **b** Murine sepsis scores at specified intervals (0, 3, 6, 12, and 24 h post-CLP). *n* = 6 per group. **c**, **d** Serum ALT and AST concentrations 24 h post-CLP. *n* = 6 per group. **e** Protein expression of HMGB1 in liver tissue 24 h post-CLP. *n* = 6 per group. f Histological changes in liver tissue observed via HE staining (the arrows indicate infiltration of inflammatory cells), scale bar: 100 μm. **g** Oil Red O staining of liver sections highlighting lipid droplet accumulation, scale bar: 100 μm. *n* = 6 per group. **h** Representative confocal microscopy images of liver sections stained for HMGB1 (red), albumin (green), and nuclei (blue); scale bar: 100 μm. Data are presented as mean ± SEM. * *p* < 0.05; ** *p* < 0.01; *** *p* < 0.001; ns, not significant. CLP, cecal ligation and puncture

Previous experiments have demonstrated that both sepsis and CL316243 can stimulate BAT to secrete Nrg4. To investigate whether Nrg4 mitigates sepsis-induced liver injury, we administered Nrg4 protein to CLP mice. We injected rNrg4 into mice 24 h before CLP surgery, and then measured the Nrg4 content in mouse serum at 0, 6, 12, and 24 h after exogenous injection of rNrg4. We found that the serum of mice maintained a high level of rNrg4 that could reach the therapeutic dose 24 h after rNrg4 injection (Fig. 5j). Compared with vehicle-treated CLP mice, recombinant Nrg4 (rNrg4) administration markedly improved survival rates (Fig. 6a) and reduced MSS, indicating alleviation of sepsis symptoms (Fig. 6b). However, there was no difference in body temperature at 24 h after CLP (Fig. S1e). Additionally, plasma ALT and AST levels were notably decreased, suggesting improved liver function (Fig. 6c and d). WB and immunofluorescence analysis showed decreased HMGB1 levels in the liver, indicating reduced liver injury (Fig. 6e and h). HE staining revealed diminished inflammatory infiltration; in contrast, Oil Red O staining indicated less fat deposition, further confirming that rNrg4 significantly reduced sepsis-induced liver damage (Fig. 6f and g). Collectively, these results show that rNrg4 significantly alleviates CLP-induced liver injury during sepsis.

### Exogenous Nrg4 Alleviates Liver Injury During Sepsis by Reducing Inflammation and Ferroptosis in the Liver

**Fig. 7:**
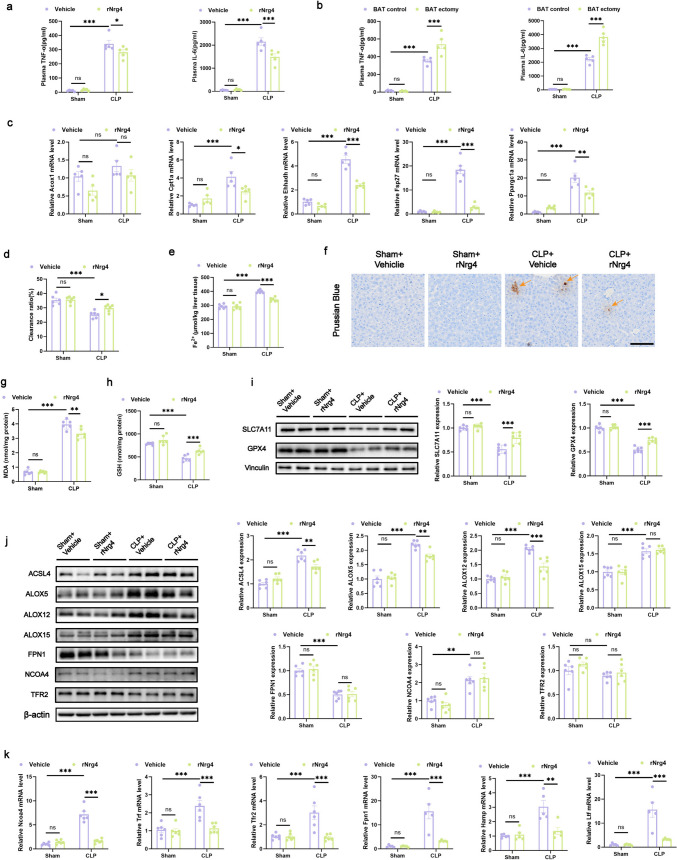
Exogenous Nrg4 alleviates liver injury during sepsis by reducing inflammation and ferroptosis in the liver (**a**, **b**) Serum concentrations of TNF-α and IL-6. *n* = 5 per group. **c** qRT-PCR analysis quantifying Acox1, Cpt1a, Ehhadh, Fap27 and Ppargc1a mRNA levels. *n* = 5 per group. **d** Hydroxyl free radical scavenging capacity in mice liver tissue. *n* = 6 per group. **e** Fe^2+^ levels in mice liver tissue. *n* = 6 per group. **f** Prussian blue staining of liver slices highlights iron in liver tissue (the arrows indicate the stained hemosiderin in the tissue), scale bar: 100 μm. (g) GSH levels in mice liver tissue. *n* = 6 per group. **h** MDA levels in mice liver tissue. *n* = 6 per group. **i** Western blot analysis showing SLC7A11 and GPX4 expression in mice liver tissue. *n* = 6 per group. **j** Western blot analysis showing ACSL4, ALOX5, ALOX12, ALOX15, FPN1, NCOA4 and TFR2 expression in mice liver tissue. *n* = 6 per group. **k** qRT-PCR analysis quantifying Ncoa4, Trf, Tfr2, Fpn1, Hamp and Ltf mRNA levels. *n* = 5–6 per group. Data are presented as mean ± SEM. * *p* < 0.05; ** *p* < 0.01; *** *p* < 0.001; ns, not significant

In order to explore the mechanism of exogenous nrg4 limiting septic liver injury after CLP, we first measured the contents of inflammatory molecules TNF- α and IL-6 in the serum of CLP mice after rNrg4 treatment, and found that compared with vehicle treatment, the contents of TNF- α and IL-6 in the serum of CLP mice in rNrg4 treatment group were significantly decreased (Fig. 7a), suggesting that the systemic inflammation of mice was significantly reduced. After that, we measured the contents of inflammatory molecules TNF- α and IL-6 in the serum of mice in the BAT control group and BAT ectomy group (Fig. 7b), and found that compared with the BAT control group, the contents of TNF- α and IL-6 in the serum of CLP mice in the BAT ectomy group were significantly increased, suggesting that the systemic inflammation of mice was significantly aggravated, which was consistent with our previous conclusion that BAT ectomy aggravated sepsis in mice.

Previous researches have shown that Nrg4 regulates liver lipid metabolism [28], so we measured the expression of genes related to lipid metabolism in the liver of CLP mice injected with rnrg4. Notably, compared with vehicle treated mice, the hepatic expression of enoyl-CoA hydratase and 3-hydroxyacyl CoA dehydrogenase (Ehhadh)and carnitine palmitoyl transferase 1a (Cpt1a), several genes involved in fatty acid β—oxidation, was significantly reduced in rnrg4 treated mice, while there was no significant difference in acyl-CoA oxidase 1 (Acox1). The expression of PPARG coactivator 1 alpha (Ppargc1a), which is involved in glucose and fatty acid metabolism, and cell death-inducing DFFA-like effector c (Fsp27), a lipid droplet protein involved in hepatic steatosis, were also significantly reduced (Fig. 7c). Moreover, there is a strong link between ferroptosis and lipid metabolism. Recent evidence highlights ferroptosis as a key factor in the progression of sepsis-induced liver injury, implying a potential regulatory function for Nrg4 in modulating ferroptosis in this context.

To explore this, we first measured the levels of hydroxy free radial scavenging capacity and Fe^2+^ in the liver of CLP mice. Compared with vehicle-treated mice, the hydroxy free radial scavenging capacity of rNrg4 group was significantly increased, while the Fe^2+^ level was significantly decreased (Fig. 7d and e). At the same time, the hemosiderin in the mice liver in rNrg4 group was significantly reduced (Fig. 7f). Meanwhile, compared with vehicle-treated mice, malondialdehyde (MDA) levels were significantly reduced in the rNrg4 group (Fig. 7g), indicating lower ferroptosis. Solute carrier family 7 member 11 (SLC7A11), glutathione (GSH), and glutathione peroxidase 4 (GPX4) are critical modulators of lipid peroxidation during ferroptosis [29]. We found a marked elevation in GSH levels in the livers of rNrg4-treated mice compared with vehicle controls (Fig. 7h). WB revealed a significant upregulation of SLC7A11 and GPX4 expression (Fig. 7i), suggesting that rNrg4 mitigated the ferroptosis observed in sepsis-induced liver injury.

To further explore the mechanism of rnrg4 improving iron death after sepsis, we determined the expression of additional molecules related to iron death. Compared with vehicle treated mice, the protein expression of acyl-CoA synthetase long chain family member 4 (ACSl4), arachidonate 5-lipoxygenase (ALOX5) and arachidonate 12-lipoxygenase (ALOX12), molecules involved in lipid synthesis in iron death, was significantly decreased in rNrg4 treated mice, while there was no significant difference in arachidonate 15-lipoxygenase (ALOX15) (Fig. 7j). The mRNA expression of transferrin (Trf), lactotransferrin (Ltf) and transferrin receptor 2 (Tfr2) involved in iron uptake was significantly decreased, while the protein expression of TFR2 was not significantly different. Meanwhile, the mRNA of nuclear receptor coactivator 4 (Ncoa4), solute carrier family 40 member 1 (Fpn1) and hepcidin antimicrobial peptide (Hamp), molecules involved in ferritin degradation, were significantly decreased in rNrg4 treated mice (Fig. 7k), while there was no difference in the protein expression of NCOA4 and FPN1 (Fig. 7j). The above results proved that Nrg4 alleviated iron death in sepsis induced liver injury, which also supported the previous conclusion.

### BAT Alleviates Liver Injury During Sepsis by Reducing Ferroptosis in the Liver

**Fig. 8:**
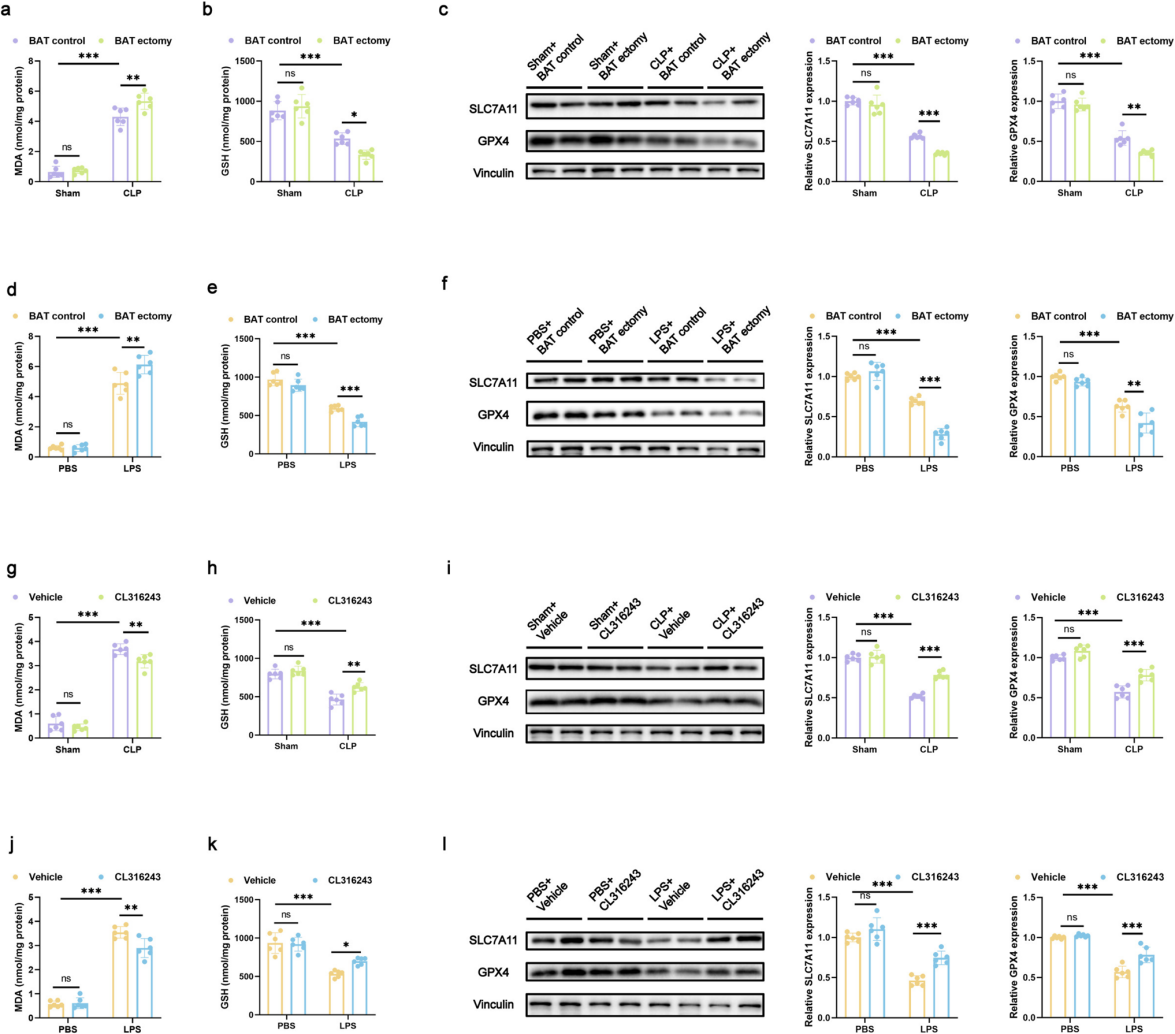
BAT alleviates liver injury during sepsis by reducing ferroptosis in the liver (**a**, **d**, **g**, **j**) GSH levels in mice liver tissue. *n* = 6 per group. **b**, **e**, **h**, **k** MDA levels in mice liver tissue. *n* = 6 per group. **c**, **f**, **i**, **l** Western blot analysis showing SLC7A11 and GPX4 expression in mice liver tissue. *n* = 6 per group. Data are presented as mean ± SEM. * *p* < 0.05; ** *p* < 0.01; *** *p* < 0.001; ns, not significant

We subsequently assessed the impact of BAT ectomy on liver ferroptosis in CLP-and LPS-induced sepsis. In CLP mice with BAT ectomy, liver MDA levels were significantly increased (Fig. 8a); in contrast, GSH, SLC7A11, and GPX4 expression levels were significantly decreased (Fig. 8b and c) compared with mice without ectomy. Similar results were seen in LPS-induced sepsis, where liver MDA levels were elevated (Fig. 8d), and GSH, SLC7A11, and GPX4 expression was diminished in BAT-excised mice (Fig. 8e and f).

Finally, we examined the impact of BAT activation on liver ferroptosis in CLP- and LPS-induced sepsis. In CLP mice treated with CL316243, liver MDA levels were significantly decreased (Fig. 8g); in contrast, GSH, SLC7A11, and GPX4 levels were significantly elevated (Fig. 8h and i). Consistent findings were observed in LPS-induced sepsis, where CL316243-treated mice showed reduced liver MDA levels (Fig. 8j) and increased GSH, SLC7A11, and GPX4 expression (Fig. 8k, l).

These findings demonstrate that BAT ectomy significantly worsen ferroptosis in septic livers caused by CLP and LPS. However, activation of BAT with CL316243 significantly alleviates ferroptosis under the same conditions. Building on previous results, we confirmed that sepsis triggers BAT activation, leading to the secretion of Nrg4, which mitigates liver ferroptosis and provides protection against liver injury.

## Discussion

**Fig. 9:**
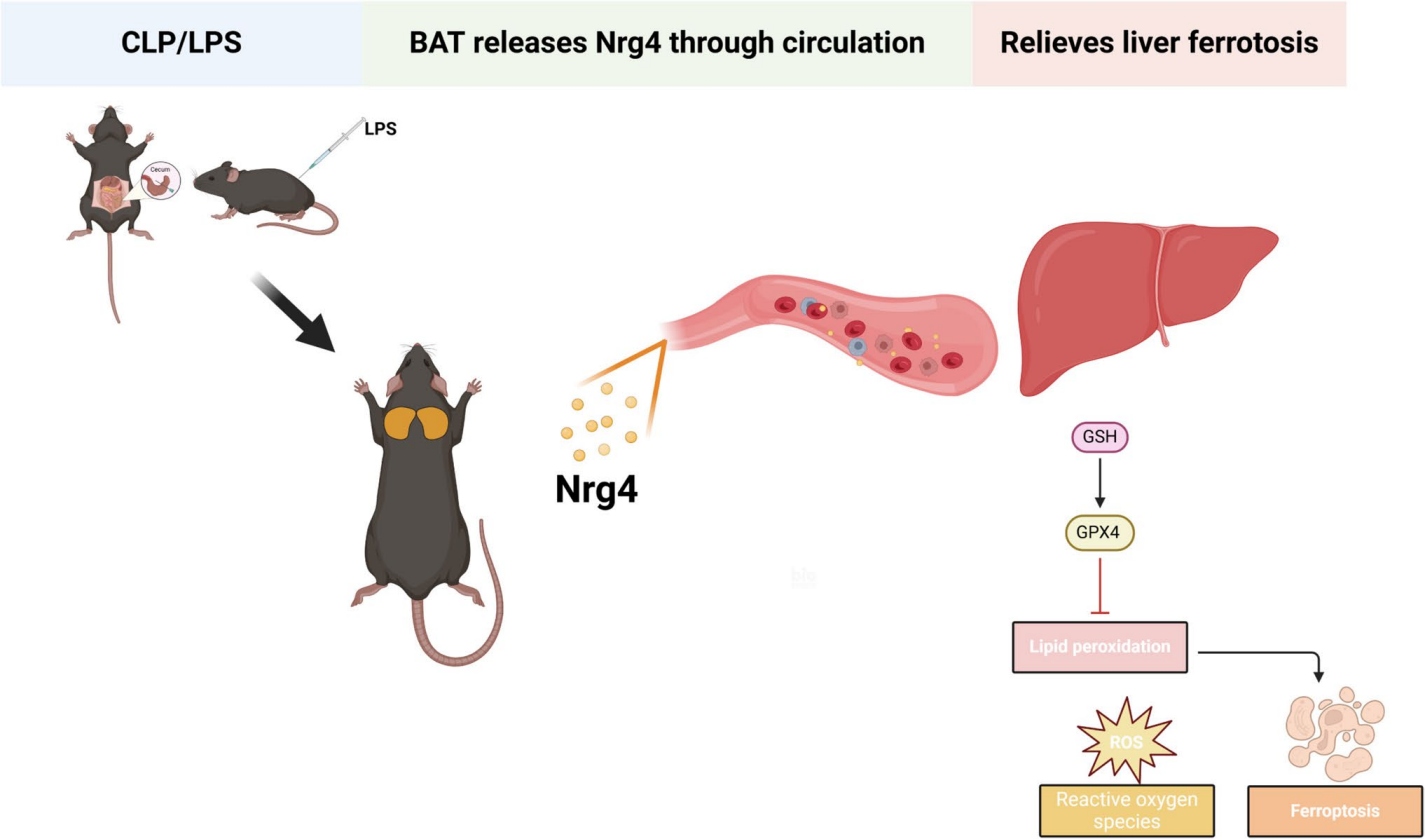
A schematic representation depicting the protective role of Nrg4, secreted by BAT, in septic liver injury During CLP- or LPS-induced sepsis, BAT is activated and releases Nrg4 into circulation, protecting the liver by inhibiting ferroptosis in septic liver injury. (Created using BioRender.com). BAT, brown adipose tissue; CLP, cecal ligation and puncture; LPS, lipopolysaccharide

In this present research, we report that BAT activation in mice following sepsis is associated with exacerbation of septic liver injury. However, pharmacological activation of BAT alleviates sepsis-induced liver damage. We identified Nrg4 as a potential hepatoprotective adipokine secreted by BAT. After sepsis, Nrg4 expression in mouse BAT increased, and plasma levels of Nrg4 rose, consistent with findings from drug-activated BAT. Exogenous Nrg4 injections alleviated liver damage and ferroptosis in septic mice by increasing GSH, SLC7A11, and GPX4 levels, ultimately reducing ferroptosis in liver cells (Fig. 9).

Sepsis, the primary cause of mortality in intensive care units, leads to systemic infections that result in fatal organ dysfunction [30], including acute liver injury, a severe and life-threatening complication that worsens patient prognosis. Currently, no specific treatment exists for sepsis-induced liver injury, highlighting the need for new therapeutic strategies to enhance liver function [3] and decrease mortality rates among sepsis patients.

Given the overactivation of the sympathetic nervous system during sepsis, which intimately linked to BAT activity, and previous studies showing that sepsis promotes the browning of WAT, we focused on BAT role in sepsis. Prior research has demonstrated that BAT protects various organs by regulating inflammation, glucose, and lipid metabolism. Among these, the liver—an important target organ for BAT-secreted cytokines—drew our attention. However, the relationship between BAT and its protective effect on the liver during sepsis has not been studied until now. Our study partially elucidated this connection. Recent studies have shown that certain physiological conditions and diseases activate BAT. For example, psychological stress may activate BAT to release IL-6, regulating the balance between pro-and anti-inflammatory responses [24]. Additionally, myocardial ischemia–reperfusion injury can activate BAT with negative effects on myocardial injury [31]. In our study, liver injury was aggravated in septic mice after BAT ectomy but significantly reduced after pharmacological activation of BAT with CL316243, confirming that sepsis activates BAT, which then exerts a protective effect against liver damage.

Despite these findings, using BAT ectomy to explore the immediate impact of its removal presents limitations due to the rapid compensatory expansion of additional BAT depots, complicating further studies on sepsis-induced injury [32, 33]. In our study, the survival rate of septic mice did not significantly decrease after BAT ectomy (Fig. 1a and b), likely due to the rapid compensation of BAT depots, which maintained some protective effects against sepsis. BAT surgical ectomy is difficult to completely remove the diffuse BAT throughout the body. In rats, surgical removal of interscapular, cervical, and axillary BAT accounts for only 40% of total BAT. After removal of these BAT, other BAT will undergo compensatory expansion [32]. In mice, although there is also compensatory expansion, the expansion of BAT after interscapular BAT removal is not as strong [33], which may be related to differences between species. Therefore, we speculate that the rapid compensation of BAT depots has a minor effect on mice, which is consistent with our experimental results. During sepsis in mice, we focused on body temperature, the change of body temperature in mice may be related to the activation and removal of BAT, but body temperature is a process of comprehensive changes, and there may be compensatory regulation in nerve and body fluid, which may not be directly related to the severity of sepsis in mice.

We observed a consistent increase in UCP1 and Nrg4 expression in activated BAT; however, no correlation between the two could be established. In a study using Nrg4 knockout mice, it was confirmed that Nrg4 secretion is not linked to UCP1, indicating that Nrg4 release may be part of BAT endocrine function rather than its thermogenic process [28].

Nrg4, a secreted cytokine, is predominantly expressed in adipose tissue, with much lower levels found in tissues such as the skeletal muscle, liver, brain, heart, and kidney [28, 34]. Nrg4 belongs to the epidermal growth factor-like extracellular ligand family and activates erb-b2 receptor tyrosine kinase 3 (Erbb3) and erb-b2 receptor tyrosine kinase 4 (Erbb4) receptor tyrosine kinases, triggering a series of downstream signaling events [35, 36]. Studies involving Nrg4 gain and loss of function in mice have conclusively shown that Nrg4 prevents diet-induced insulin resistance and hepatic steatosis by inhibiting adipogenic changes in hepatocytes [28], and it also alleviates the progression of non-alcoholic steatohepatitis by reducing the expression of inflammatory liver genes [35]. Furthermore, research indicates that Nrg4 secreted by BAT exerts anti-inflammatory effects by reducing vascular inflammation and adhesion responses [37]. Therefore, BAT-derived Nrg4 is instrumental in modulating inflammation and lipid metabolism across various organs and may act as a significant conduit between BAT and liver function. This study demonstrates that sepsis induces Nrg4 secretion from mouse BAT, which enters the circulation and offers protection to the liver. In our study, BAT was activated and the Nrg4 content increased after CLP. Because Nrg4 was highly enriched in BAT, the reduction of Nrg4 content may be mainly caused by BAT activated by CLP activation. After BAT ectomy, the content of Nrg4 in CLP group decreased significantly but was still higher than that in BAT ectomy group without CLP, possibly due to the production of Nrg4 in white adipose tissue or other parts. We will further explore the expression of Nrg4 in other Nrg4 producing tissues after CLP in future studies.

The mechanisms underlying septic acute liver injury involve inflammatory factors, oxidative stress, autophagy, and apoptosis. Increasing evidence highlights the critical function of ferroptosis in the initiation and progression of septic liver injury. [38, 39]. The liver is rich in mitochondria, and many of its functions depend on mitochondrial energy production. During sepsis, mitochondrial reactive oxygen species (ROS) levels increase, causing ROS-induced mitochondrial damage, leading to dysfunction and subsequent liver failure. Mitochondrial oxidative is a pivotal factor contributing significantly to organ dysfunction in sepsis [40, 41], and there is a strong association between mitochondrial oxidative stress and ferroptosis. Ferroptosis, an iron-dependent form of cell death, is initiated by lipid peroxidation, primarily caused by free iron and ROS. An imbalance between the oxidative defense system and oxidative damage characterizes this process [42, 43]. Key regulators of ferroptosis include SLC7A11, GSH, and GPX4. In the process of ferroptosis, there is a reduction in the levels of GPX4 and GSH, which consequently triggers lipid peroxidation. [29]. SLC7A11, an amino acid antiporter, is involved in the cysteine-glutamate exchange system, which facilitates the absorption of extracellular cysteine and the export of glutamate. It promotes the synthesis of GSH and GPX4, enhances antioxidant activity, and reduces lipid peroxidation, thereby preventing iron accumulation [44]. The cystine-glutamate reverse transport system facilitates the uptake of cysteine, which in turn promotes GSH synthesis and enhances the antioxidant activity of GPX4, thereby inhibiting ferroptosis. [44, 45]. Our study is the first to show that BAT can reduce liver ferroptosis by secreting Nrg4, thereby protecting against sepsis-induced liver injury.

In conclusion, BAT is activated during sepsis and protects against liver injury in mice. Our research has pinpointed Nrg4 as a crucial liver-protective protein that is secreted by BAT subsequent to sepsis-induced liver damage. The findings indicate that BAT-derived Nrg4 suppresses ferroptosis and attenuates sepsis-induced liver injury by promoting the expression of SLC7A11, GSH, and GPX4, suggesting potential therapeutic application of BAT activation and its secreted Nrg4 in treating septic liver injury. However, this study has some limitations. The precise relationship between Nrg4 secretion and BAT thermogenic activation has not been fully explored, nor is it clear whether Nrg4 is a critical mediator of protection against sepsis-induced liver damage. Additionally, Nrg4 role in damage to other organs during sepsis remains understudied. This study highlights the crosstalk between BAT and septic liver injury, and future research could focus on using BAT activation or Nrg4 as a therapeutic approach to mitigate liver injury in sepsis, providing valuable guidance for treating sepsis-related liver damage.

## Materials and Methods

### Mice and Sepsis Model

The mice were obtained from the Fourth Military Medical University in Xi'an, China. The animals were maintained in a controlled environment characterized by a consistent temperature of 22 °C and a 12-h light–dark cycle, with unlimited access to food and water. The mice were reared until they attained the age of 8 weeks. During the experiment, male mice were randomly allocated to either the sham surgery group or the sepsis model group.

Sepsis was induced using CLP in mice at the age of 8–10 weeks. Mice were first anesthetized with 2% isoflurane. The distal one-third of the cecum was ligated, and a 22-gauge needle was used to puncture it, allowing a minor quantity of intestinal contents to be expressed. Mice were then used for experiments 24 h post-procedure.

Sepsis was also induced using LPS in mice at the age of 8–10 weeks. Mice were injected intraperitoneally with LPS (10 mg kg^−1^, L2880, Sigma-Aldrich) and used for experiments 24 h later.

### Animal Procedures

For the BAT ectomy study, male mice underwent either sham surgery or scapular BAT ectomy under isoflurane anesthesia, followed by CLP/LPS for 72 h or sham treatment for 72 h. The mice were compassionately euthanized 24 h after the CLP/LPS procedure to harvest tissue and plasma.

In the in vivo CL316243 (HY-116771A, MCE) injection studies, male mice received 1 mg kg^−1^ day^−1^ CL316243 via intraperitoneal injection. On the 7th day of injection, mice were subjected to CLP/LPS or sham/PBS treatment and were euthanized 24 h post-CLP/LPS for tissues and plasma collection.

For the rNrg4 (RPC174Mu01, CLOUD-CLONE Corp) injection in vivo studies, male mice were administered 1 mg kg^−1^ day^−1^ CL316243 intraperitoneally. 24 h before CLP/LPS or sham treatment, mice received an intravenous injection of rNrg4 (100 μg kg^−1^). Mice were then subjected to CLP/LPS or sham/PBS treatment and euthanized 24 h post-CLP/LPS to collect tissues and plasma.

### MSS

MSS was used to assess disease severity in a fecal-induced peritonitis model, as detailed in Table 1 on page 157 of *Scoring Sepsis Severity in Mice* [25]*.*

### WB

WB was performed using RIPA buffer (Beyotime, China), that incorporated a combination of protease and phosphatase inhibitors(Beyotime), for the lysis of tissues to enable protein extraction. The concentration of protein was accurately determined utilizing an enhanced BCA protein assay kit (Beyotime). Identical quantities of protein were extracted via SDS-PAGE and subsequently transferred onto a polyvinylidene fluoride membrane. The membranes underwent blocking with TBST buffer enriched with 2% bovine serum albumin for 1 h, subsequently subjected to an overnight incubation process with primary antibodies. After that, membranes underwent incubation with secondary antibodies. Protein bands were detected with an ECL substrate (Elabscience) and analyzed with ImageJ software.

The antibodies used in this study included anti-β actin (AC026, Abclonal, RRID:AB_2768234), anti-HMGB1 (10,829–1-AP, Proteintech, RRID:AB_2232989), anti-SLC7A11 (26,864–1-AP, Proteintech, RRID:AB_2880661), anti-GPX4 (ab125066, Abcam, RRID:AB_10973901), anti-UCP1 (23,673–1-AP, Proteintech, RRID:AB_2828003), anti-Nrg4 (PA5-102,641, Invitrogen), anti-ACSL4 (sc-365230, Santa cruz, RRID: AB_10843105), anti-ALOX5 (sc-136195, Santa cruz, RRID: AB_2274005), anti-ALOX12 (sc-365194, Santa cruz, RRID: AB_10709144), anti-ALOX15 (A6864, Abclonal, RRID: AB_2767425), anti-FPN1 (sc-518125, Santa cruz), anti-NCOA4 (10,968–1-AP, Proteintech), and anti-TFR2 (A9845, Abclonal, RRID: AB_2772579). The secondary antibodies were horseradish peroxidase-conjugated goat anti-rabbit IgG (A0208, Beyotime, RRID:AB_2892644) and horseradish peroxidase-conjugated goat anti-mouse IgG (A0216, Beyotime, RRID:AB_2860575)..

### ALT and AST Assay

The concentrations of serum ALT and AST were determined using ALT assay kits (E-BC-K235-M, Elabscience) and AST assay kits (E-BC-K236-M, Elabscience), following the manufacturer's instructions.

### Nrg4 ELISA Assay

The concentrations of serum Nrg4 concentrations were determined using ELISA kits (CSB-EL016080MO, Cusabio) according to the manufacturer's protocols.

### MDA and GSH Assay

Serum MDA levels were measured using MDA assay kits (S0131S; Beyotime), and serum GSH levels were assessed using GSH assay kits (A006-2–1; Njjc Bio), following the manufacturers’ guidelines.

### Ferrous Iron Assay and Hydroxyl Free Radical Scavenging Capacity

The ferrous iron (Fe^2+^) levels in the liver tissue were measured using ferrous iron colorimetric assay kit (E-BC-K773-M; Elabsicence), and hydroxyl free radical cavenging capacity in the liver tissue were assessed using hydroxyl free radical scavenging capacity assay kit (E-BC-K527-M; Elabsicence), following the manufacturers’ guidelines.

### Immunofluorescence and Fluorescence Imaging

Liver and adipose tissue samples were preserved in 4% paraformaldehyde (PFA), embedded in paraffin, and sliced into sections measuring sliced into sections with a thickness of 5 μm. After dewaxing and rehydration, antigen retrieval was car-ried out using an ethylenediaminetetraacetic acid-based solution (pH 9.0) in a boiling water bath for 20 min. The sections were then permeabilized with 0.3% Triton X-100 and blocked with QuickBlock™ Immunostaining Blocking Buffer (Beyotime). Primary antibodies were applied to the sections, which were incubated overnight. Fluorescent secondary antibodies were added and incubated in the dark for 2 h, followed by nuclei counterstaining with DAPI (Sigma Aldrich, St. Louis, Mis-souri, USA). The following antibodies were used: anti-HMGB1 (10,829–1-AP, Proteintech, RRID:AB_2232989), anti-albumin (66,051–1-Ig, Proteintech, RRID:AB_11042320), anti-PLIN (ab61682, Abcam, RRID:AB_944751), and anti-Nrg4 (PA5-102,641, Invitrogen). Secondary antibodies conjugated to fluorescent dyes included goat anti-rabbit Alexa Fluor 555 (ab150078, Abcam, RRID:AB_2722519), goat anti-mouse Alexa Fluor 488 (ab150113, Abcam, RRID:AB_2576208), and donkey anti-goat Alexa Fluor 488 (ab150129, Abcam, RRID:AB_2687506). Visualization was performed using a Virtual Slide Microscopy VS200 (OLYMPUS, Japan).

#### Histopathological Analysis

Liver and adipose tissues were fixed in 4% PFA, embedded in paraffin, and sectioned into 5 μm thick slices. After deparaffinization and rehydration, the sections were stained with HE (Servicebio, China) and Prussian Blue (Servicebio, China) according to the manufacturer's protocol, and images were captured utilizing a Virtual Slide Microscopy VS200 (Olympus, Japan).

For further analysis, liver tissues were embedded in an optimal cutting temperature compound (Sakura, Japan). They were sectioned into 5 µm slices, stained with Oil Red O (Servicebio, China), and visualized with a Virtual Slide Microscope VS200 (Olympus, Japan).

#### Quantitative Polymerase Chain Reaction (qPCR)

Total RNA was extracted using RNA simple (TIANGEN, China) and reverse-transcribed into cDNA using StarLighter Script RT all-in-one mix (FOREVER STAR, China). qPCR was performed on a CFX Connect Real-Time PCR Detection System (Bio-Rad, USA) using SYBR Green PCR Mix (FOREVER STAR, China). ACTB/β-actin mRNA levels were used for normalization, And target gene expression was calculated using the 2^^−ΔΔCt^ method.

#### Statistical Analysis

Data are presented as the mean ± standard error of the mean. Differences between the two groups were analyzed using an unpaired Student's t-test. For comparisons among four groups, a two-way analysis of variance (ANOVA) followed by Tukey’s post hoc test was used. For three-group comparisons, one-way ANOVA followed by Tukey’s post hoc test was applied (GraphPad Prism 8). Statistical significance was set at *p* < 0.05.

## Supplementary Information

Below is the link to the electronic supplementary material.

Supplementary file1 (PDF 2.70 MB)

## Data Availability

Data will be made available on request.
