# Supplementary material for: Nrg4 Secreted by Brown Adipose Tissue Suppresses Ferroptosis of Sepsis-Induced Liver Injury
Source: Inflammation. 2025 Feb 17;48(4):2783–801. doi: 10.1007/s10753-024-02230-z (PMC12336088; doi:10.1007/s10753-024-02230-z)
Supplement: Supplementary file 1 — Supplementary file1 (PDF 2.70 MB) [file 10753_2024_2230_MOESM1_ESM.pdf]

Fig. S1

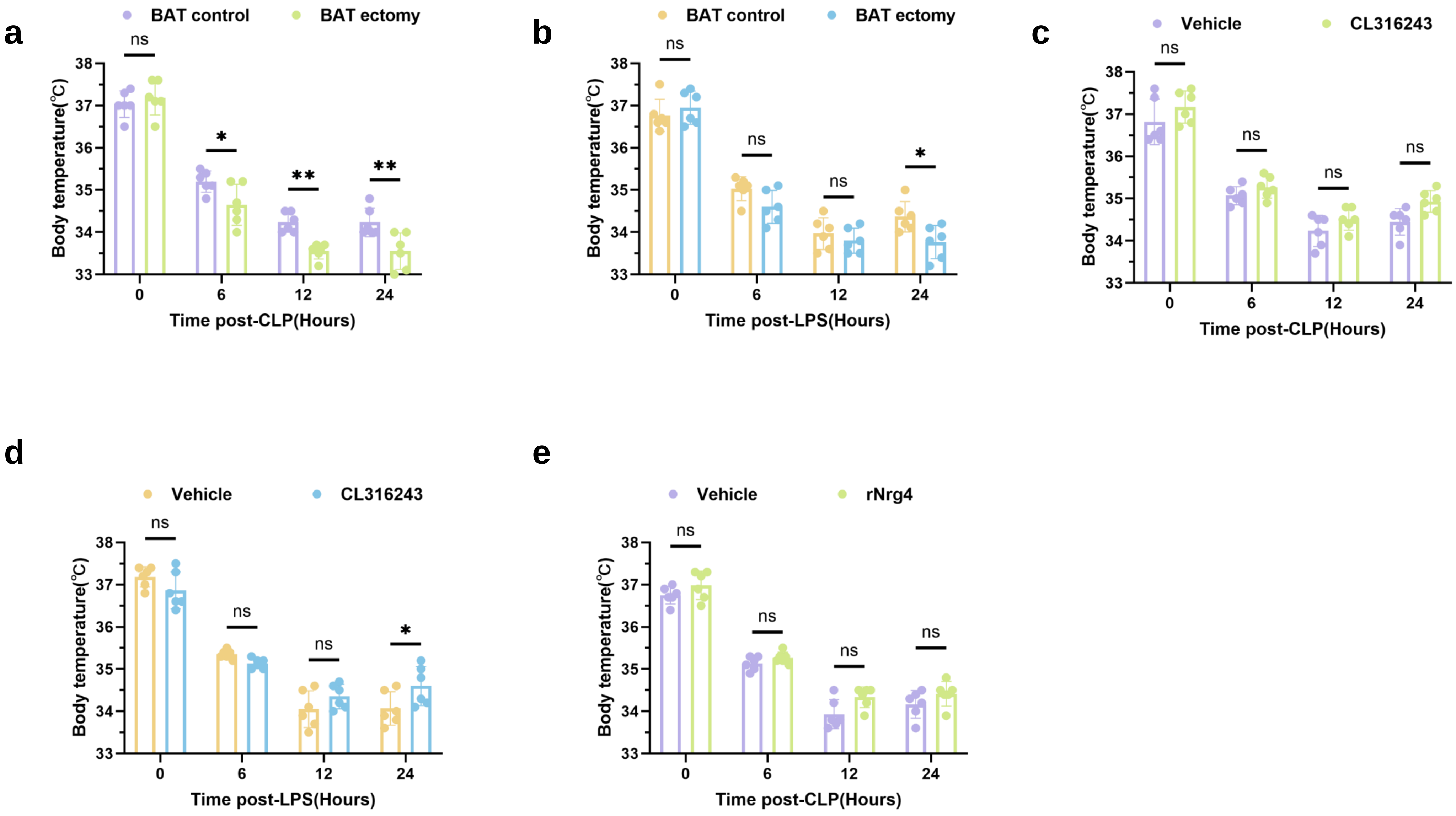
a
BAT control
BAT ectomy
Body temperature(℃)
Time post-CLP(Hours)
b
BAT control
BAT ectomy
Body temperature(℃)
Time post-LPS(Hours)
c
Vehicle
CL316243
Body temperature(℃)
Time post-CLP(Hours)
d
Vehicle
CL316243
Body temperature(℃)
Time post-LPS(Hours)
e
Vehicle
rNrg4
Body temperature(℃)
Time post-CLP(Hours)

Figure 1e

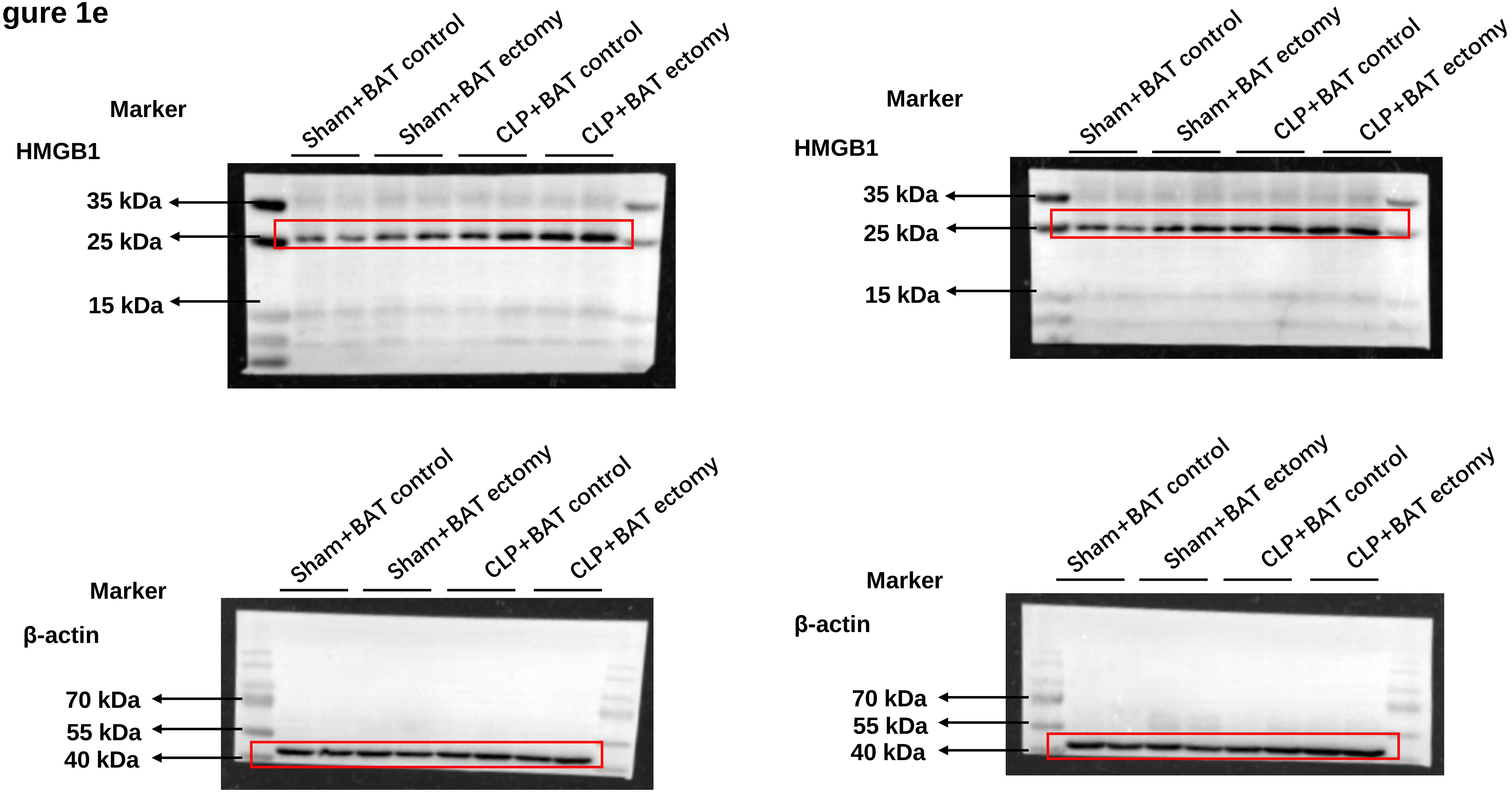
Marker
HMGB1
Sham+BAT control
Sham+BAT ectomy
CLP+BAT control
CLP+BAT ectomy
35 kDa
25 kDa
15 kDa
Marker
HMGB1
Sham+BAT control
Sham+BAT ectomy
CLP+BAT control
CLP+BAT ectomy
35 kDa
25 kDa
15 kDa
Marker
β-actin
Sham+BAT control
Sham+BAT ectomy
CLP+BAT control
CLP+BAT ectomy
70 kDa
55 kDa
40 kDa
Marker
β-actin
Sham+BAT control
Sham+BAT ectomy
CLP+BAT control
CLP+BAT ectomy
70 kDa
55 kDa
40 kDa

Figure 1e

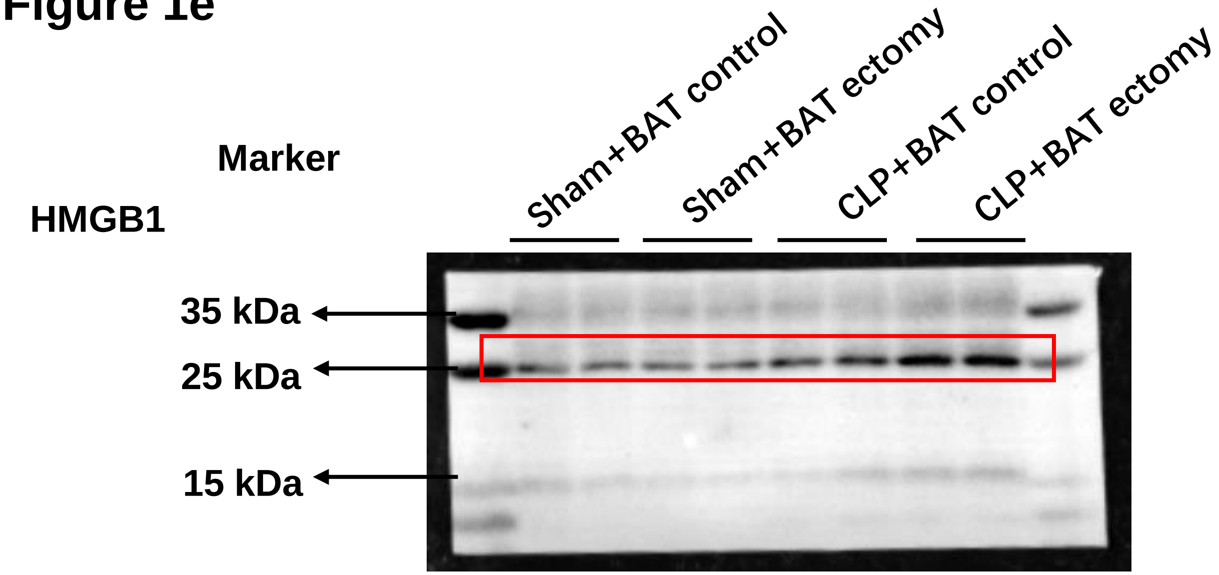

HMGB1
Marker
Sham+BAT control
Sham+BAT ectomy
CLP+BAT control
CLP+BAT ectomy
35 kDa
25 kDa
15 kDa

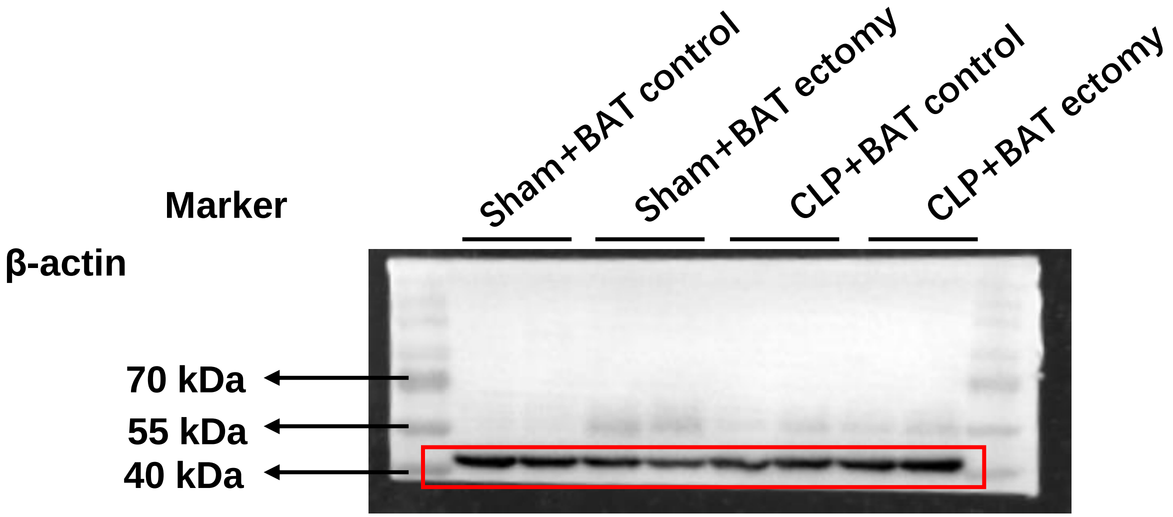

β-actin
Marker
Sham+BAT control
Sham+BAT ectomy
CLP+BAT control
CLP+BAT ectomy
70 kDa
55 kDa
40 kDa

Figure 2e

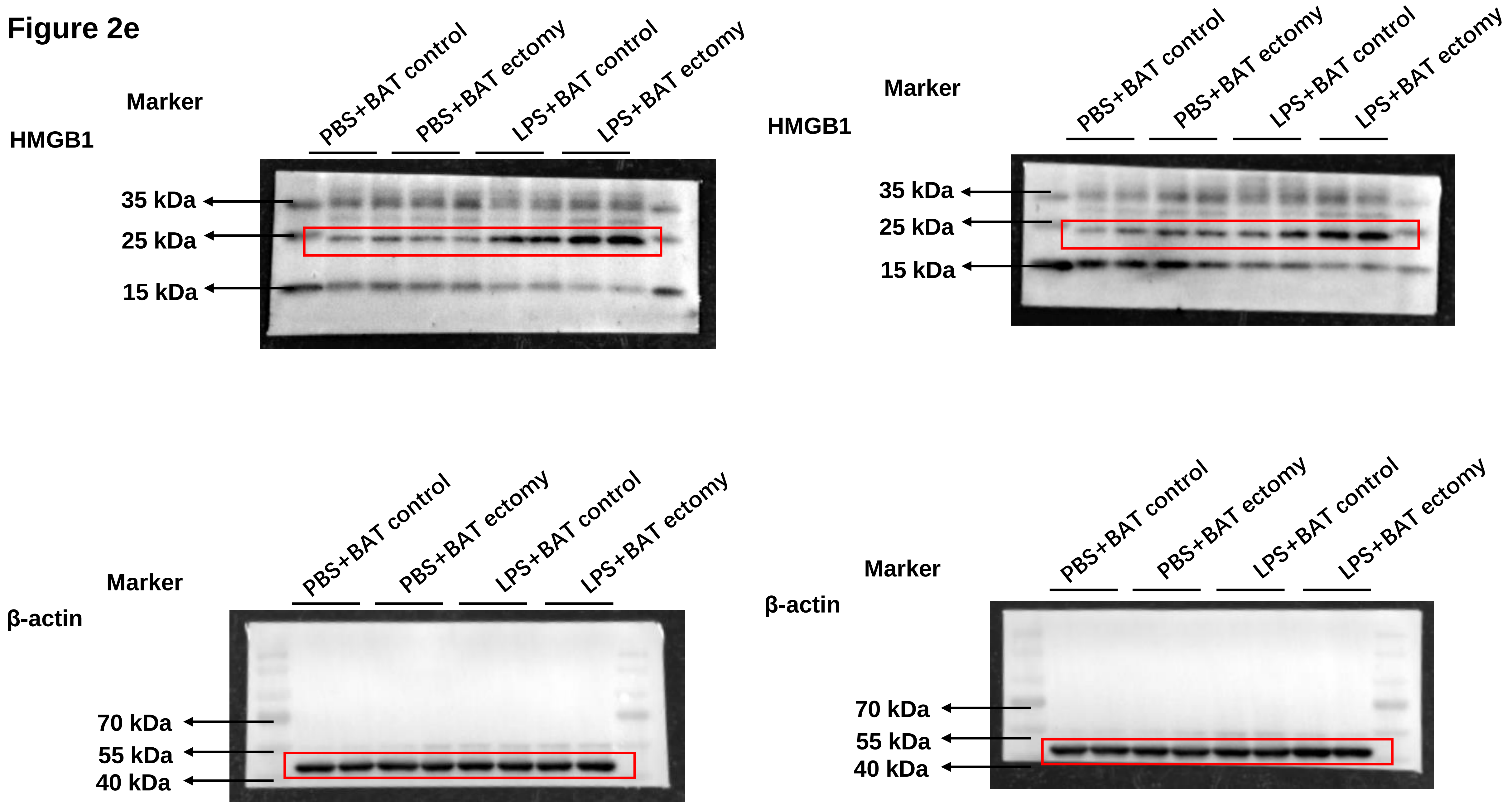

HMGB1
Marker
PBS+BAT control
PBS+BAT ectomy
LPS+BAT control
LPS+BAT ectomy
35 kDa
25 kDa
15 kDa
HMGB1
Marker
PBS+BAT control
PBS+BAT ectomy
LPS+BAT control
LPS+BAT ectomy
35 kDa
25 kDa
15 kDa
β-actin
Marker
PBS+BAT control
PBS+BAT ectomy
LPS+BAT control
LPS+BAT ectomy
70 kDa
55 kDa
40 kDa
β-actin
Marker
PBS+BAT control
PBS+BAT ectomy
LPS+BAT control
LPS+BAT ectomy
70 kDa
55 kDa
40 kDa

Figure 2e

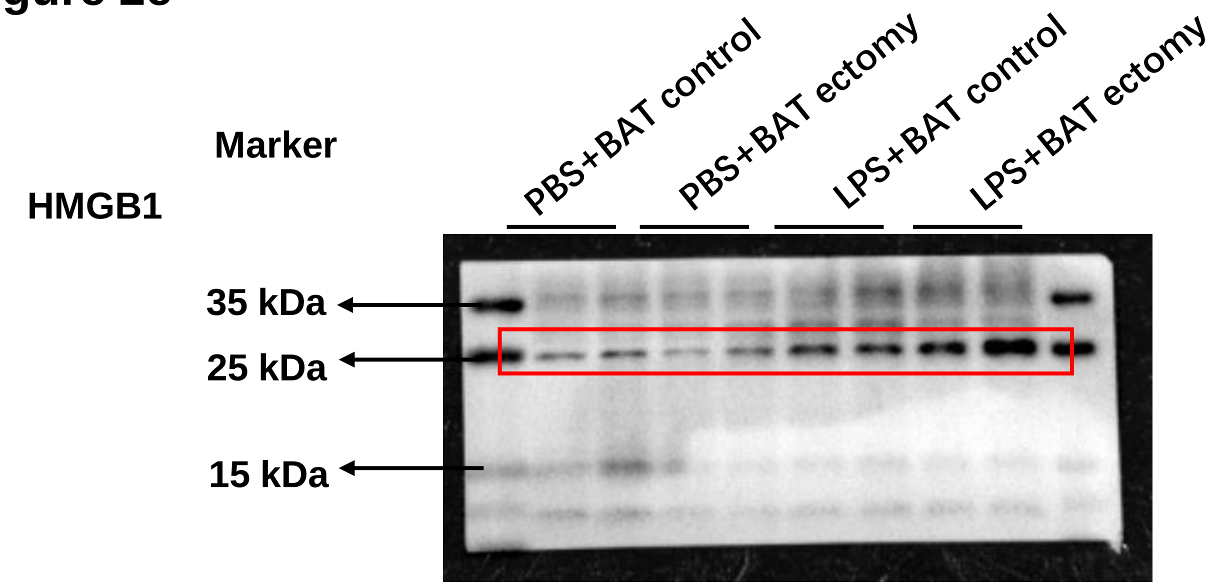
HMGB1
Marker
PBS+BAT control
PBS+BAT ectomy
LPS+BAT control
LPS+BAT ectomy
35 kDa
25 kDa
15 kDa

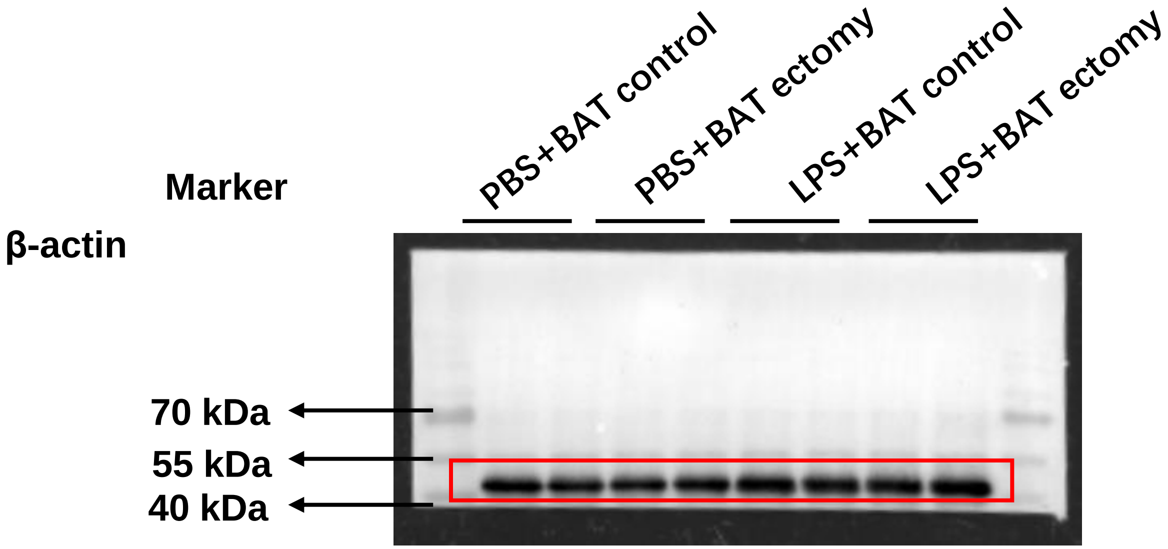
β-actin
Marker
PBS+BAT control
PBS+BAT ectomy
LPS+BAT control
LPS+BAT ectomy
70 kDa
55 kDa
40 kDa

Figure 3e

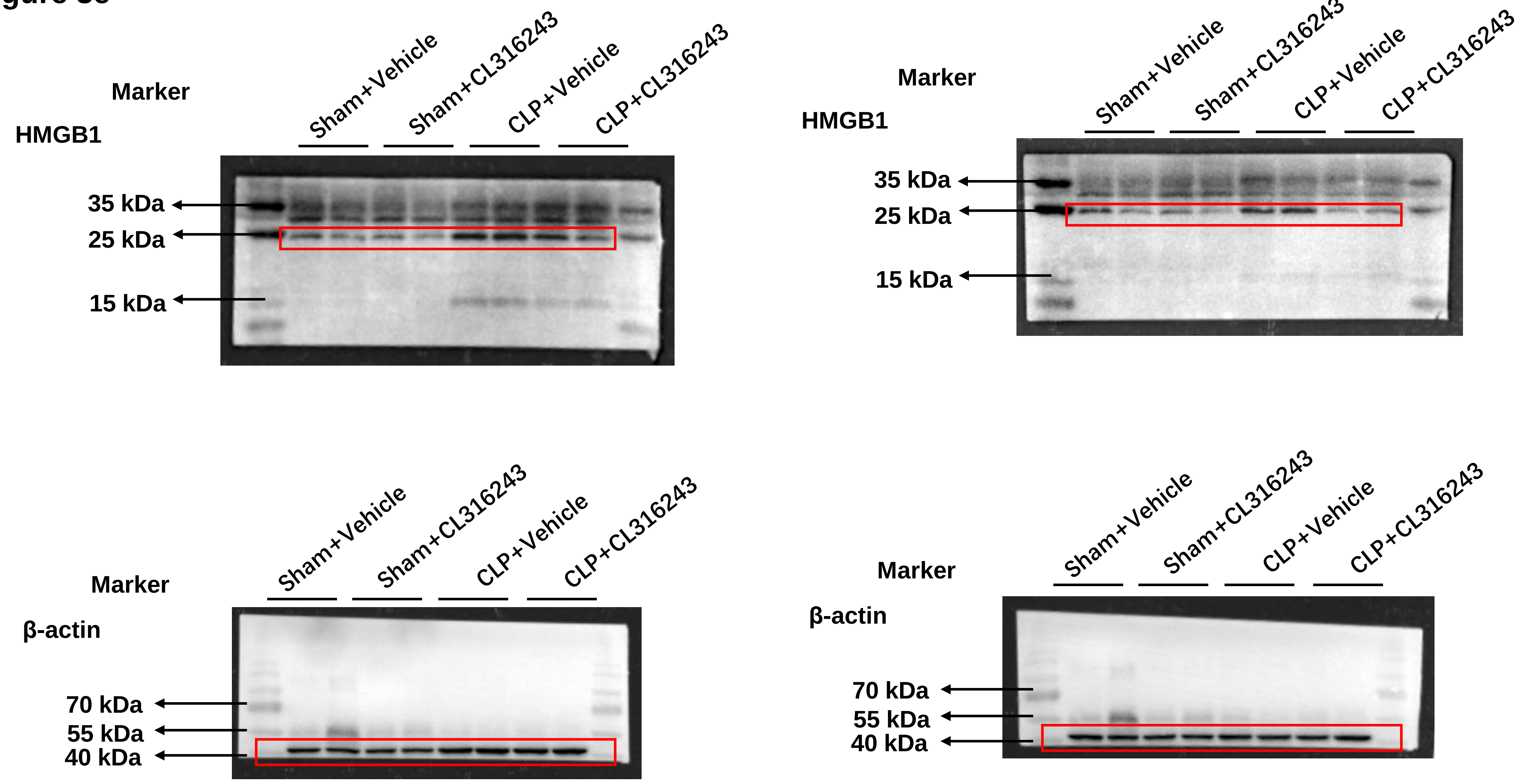

Marker
HMGB1
Sham+Vehicle
Sham+CL316243
CLP+Vehicle
CLP+CL316243
35 kDa
25 kDa
15 kDa
Marker
HMGB1
Sham+Vehicle
Sham+CL316243
CLP+Vehicle
CLP+CL316243
35 kDa
25 kDa
15 kDa
Marker
β-actin
Sham+Vehicle
Sham+CL316243
CLP+Vehicle
CLP+CL316243
70 kDa
55 kDa
40 kDa
Marker
β-actin
Sham+Vehicle
Sham+CL316243
CLP+Vehicle
CLP+CL316243
70 kDa
55 kDa
40 kDa

Figure 3e

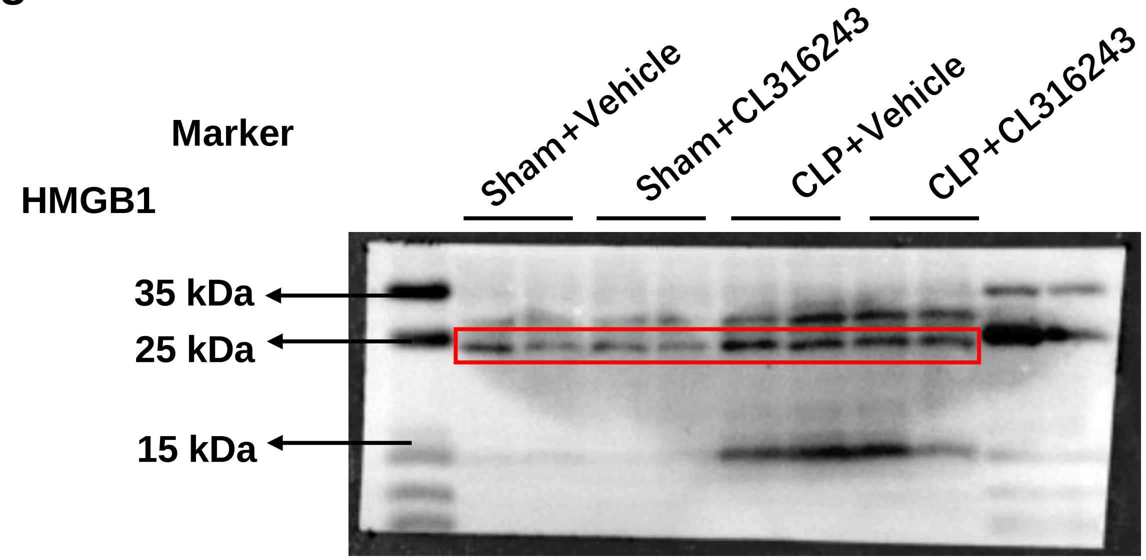

Sham+Vehicle
Sham+CL316243
CLP+Vehicle
CLP+CL316243
Marker
HMGB1
35 kDa
25 kDa
15 kDa

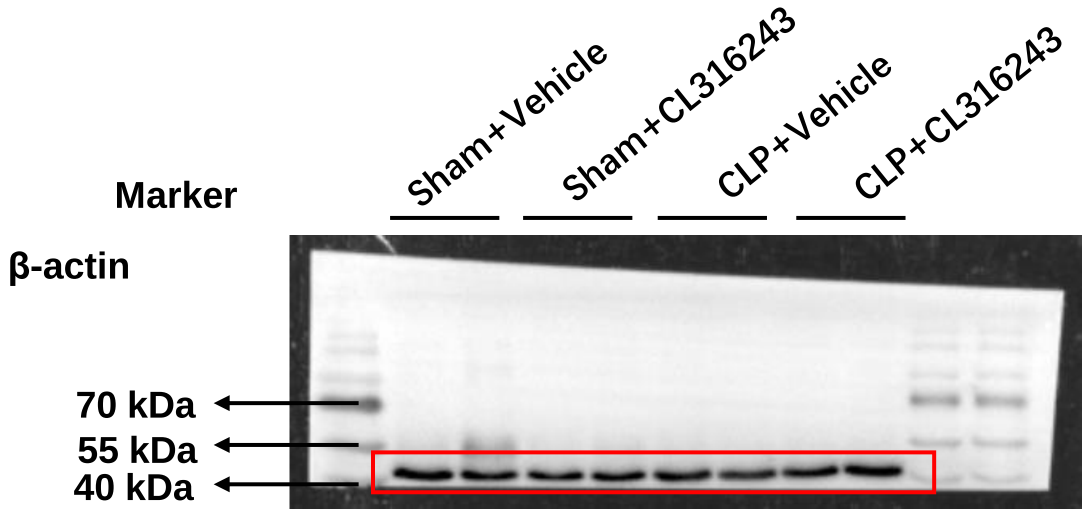

Sham+Vehicle
Sham+CL316243
CLP+Vehicle
CLP+CL316243
Marker
β-actin
70 kDa
55 kDa
40 kDa

Figure 4e

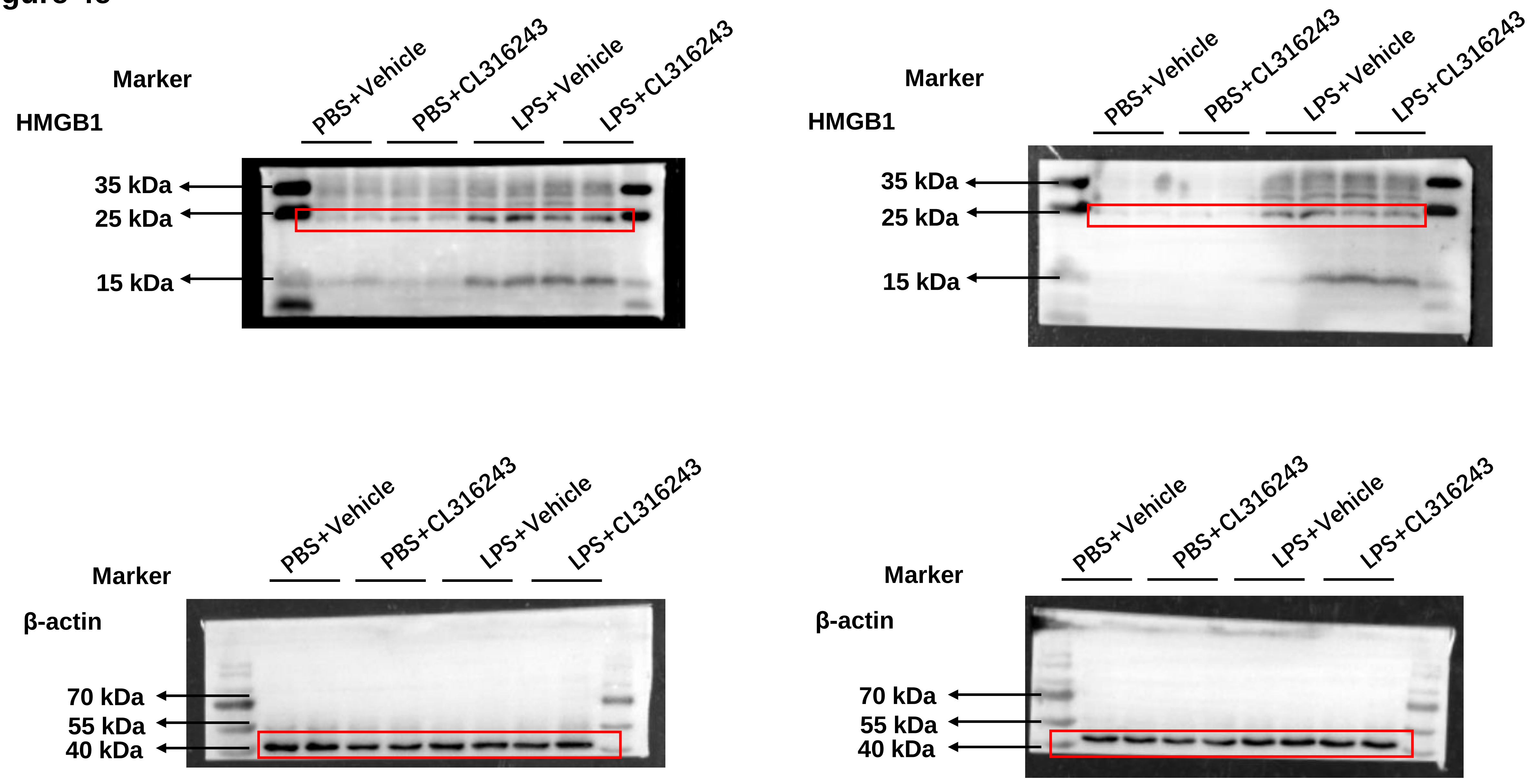
Marker
HMGB1
PBS+Vehicle
PBS+CL316243
LPS+Vehicle
LPS+CL316243
35 kDa
25 kDa
15 kDa
Marker
HMGB1
PBS+Vehicle
PBS+CL316243
LPS+Vehicle
LPS+CL316243
35 kDa
25 kDa
15 kDa
Marker
β-actin
PBS+Vehicle
PBS+CL316243
LPS+Vehicle
LPS+CL316243
70 kDa
55 kDa
40 kDa
Marker
β-actin
PBS+Vehicle
PBS+CL316243
LPS+Vehicle
LPS+CL316243
70 kDa
55 kDa
40 kDa

Figure 4e

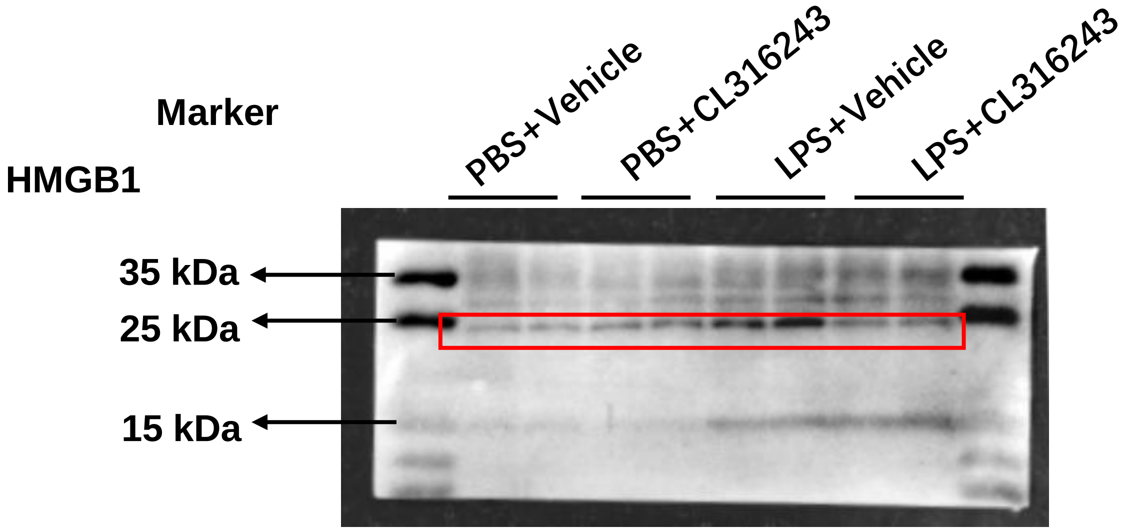

Marker
HMGB1
PBS+Vehicle
PBS+CL316243
LPS+Vehicle
LPS+CL316243
35 kDa
25 kDa
15 kDa

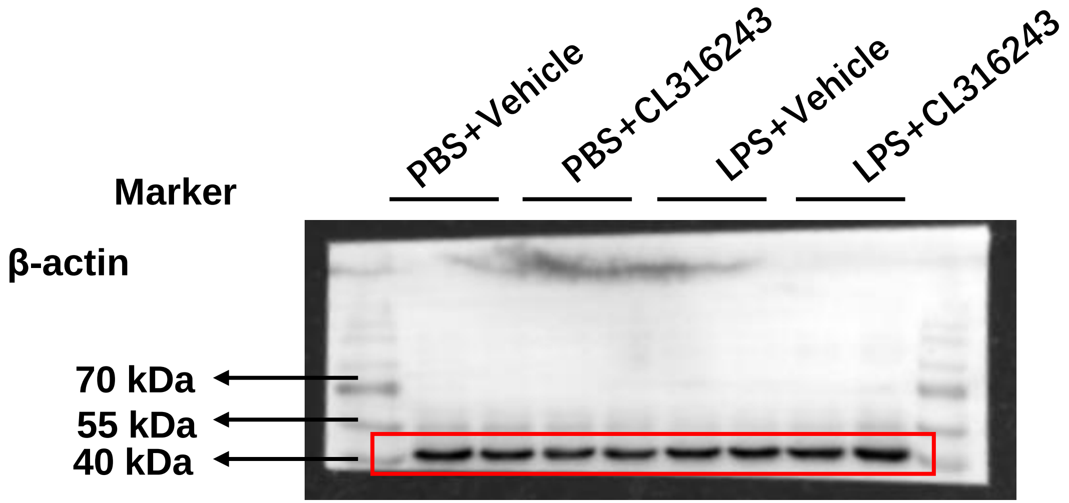

Marker
β-actin
PBS+Vehicle
PBS+CL316243
LPS+Vehicle
LPS+CL316243
70 kDa
55 kDa
40 kDa

Figure 5f

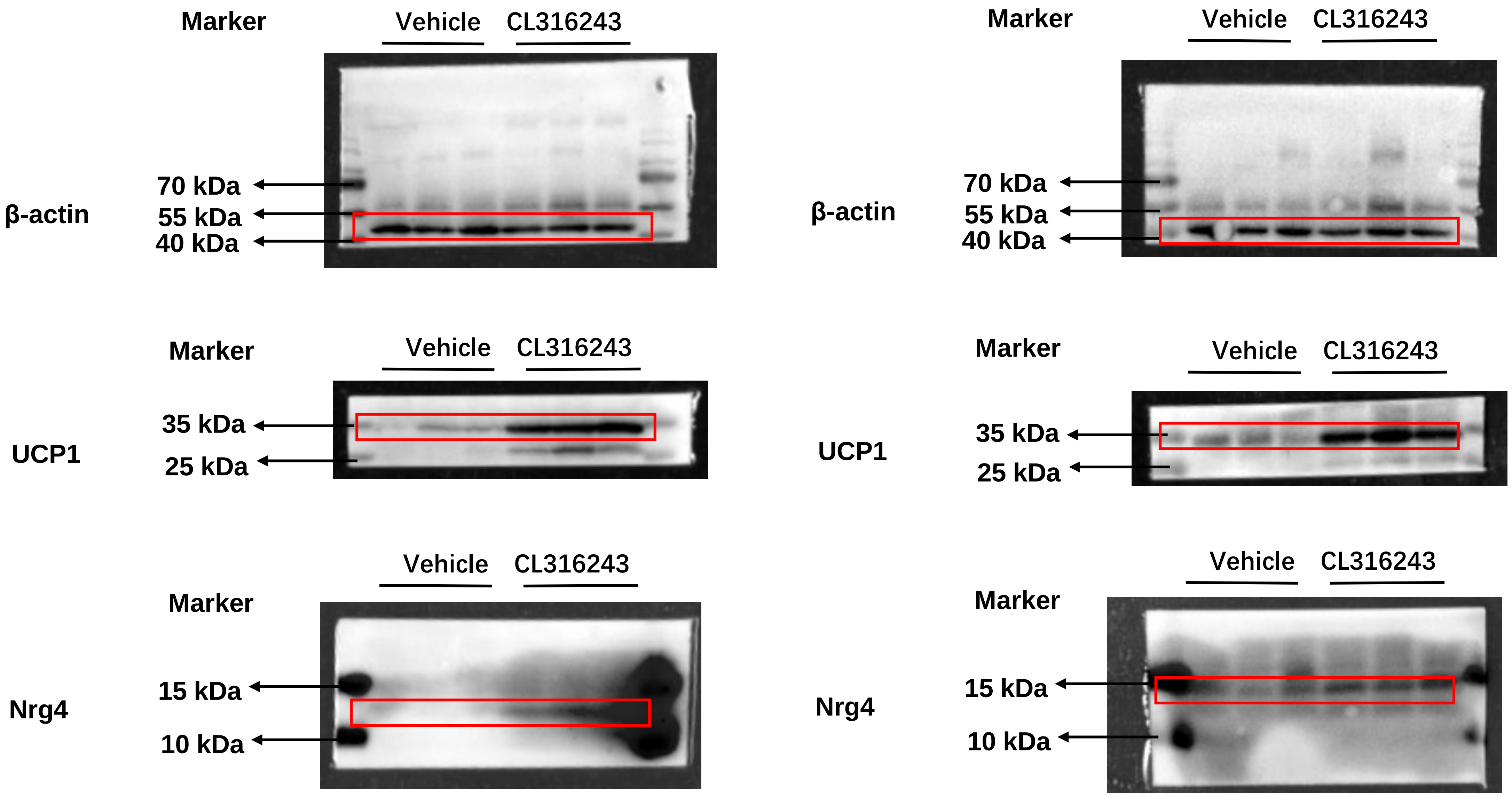

Marker
Vehicle
CL316243
β-actin
70 kDa
55 kDa
40 kDa
Marker
Vehicle
CL316243
β-actin
70 kDa
55 kDa
40 kDa
Marker
Vehicle
CL316243
UCP1
35 kDa
25 kDa
Marker
Vehicle
CL316243
UCP1
35 kDa
25 kDa
Vehicle
CL316243
Marker
Nrg4
15 kDa
10 kDa
Vehicle
CL316243
Marker
Nrg4
15 kDa
10 kDa

Figure 6e

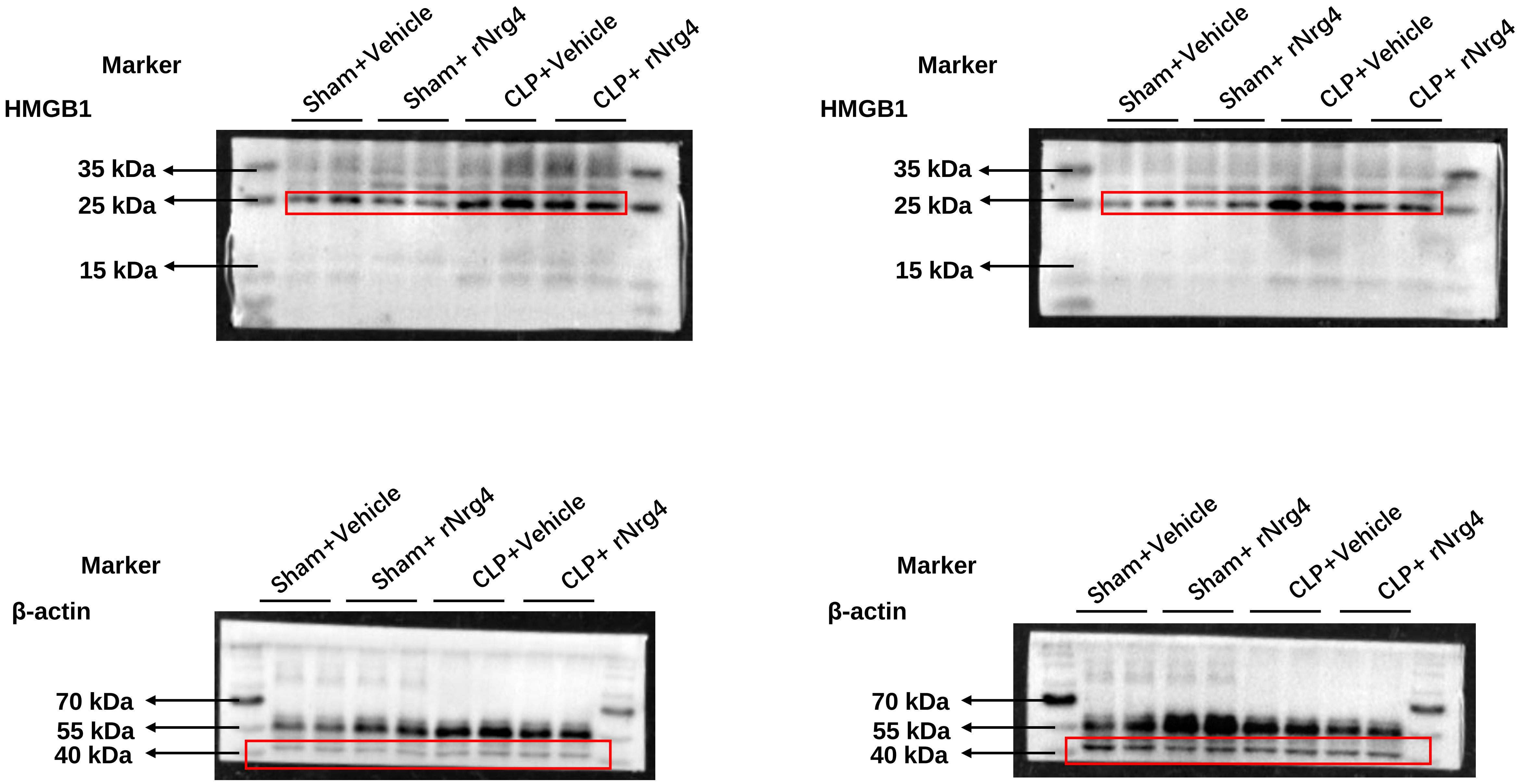
Marker
HMGB1
Sham+Vehicle
Sham+ rNrg4
CLP+Vehicle
CLP+ rNrg4
35 kDa
25 kDa
15 kDa
Marker
HMGB1
Sham+Vehicle
Sham+ rNrg4
CLP+Vehicle
CLP+ rNrg4
35 kDa
25 kDa
15 kDa
Marker
β-actin
Sham+Vehicle
Sham+ rNrg4
CLP+Vehicle
CLP+ rNrg4
70 kDa
55 kDa
40 kDa
Marker
β-actin
Sham+Vehicle
Sham+ rNrg4
CLP+Vehicle
CLP+ rNrg4
70 kDa
55 kDa
40 kDa

Figure 6e

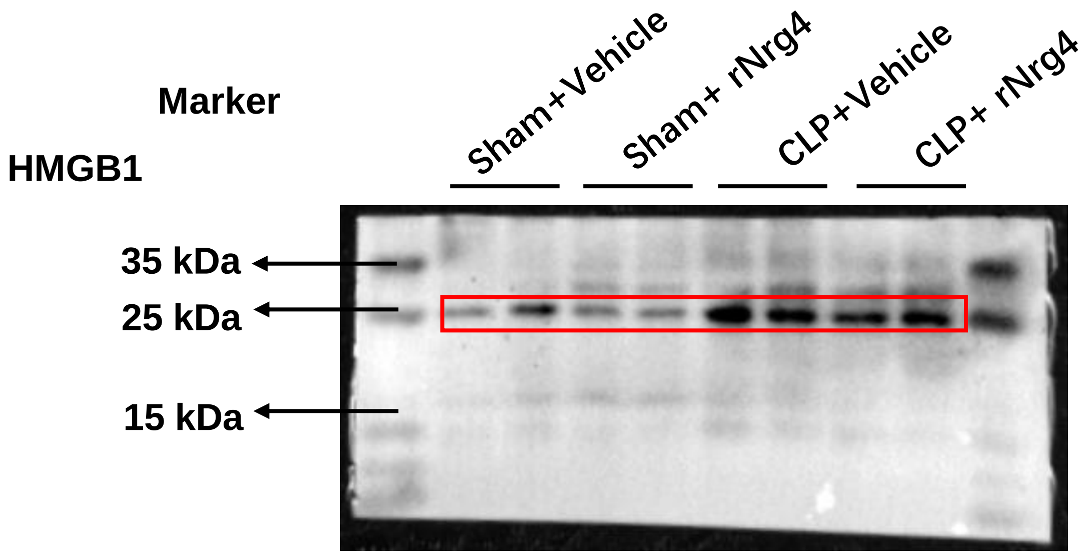
Marker
HMGB1
Sham+Vehicle
Sham+ rNrg4
CLP+Vehicle
CLP+ rNrg4
35 kDa
25 kDa
15 kDa

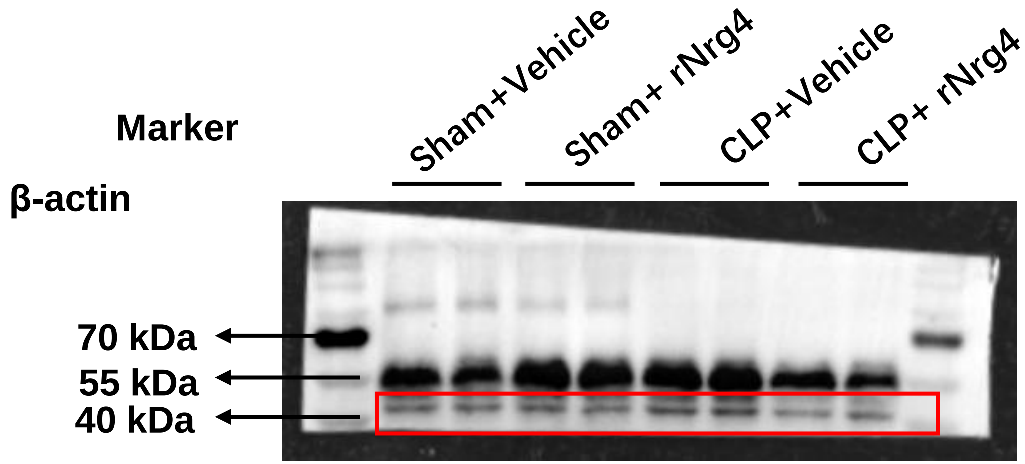
Marker
β-actin
Sham+Vehicle
Sham+ rNrg4
CLP+Vehicle
CLP+ rNrg4
70 kDa
55 kDa
40 kDa

Figure 7j

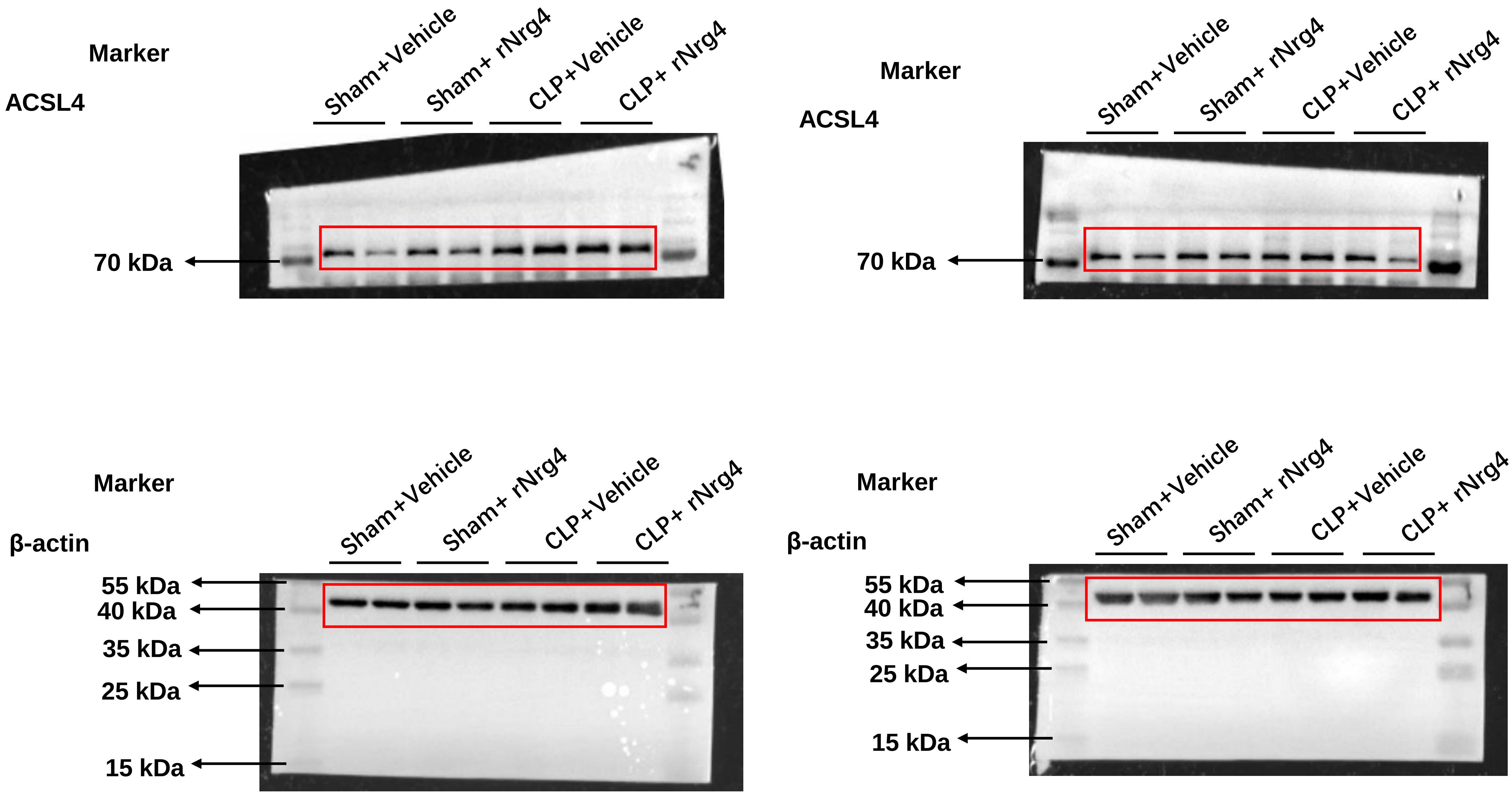

Marker
ACSL4
Sham+Vehicle
Sham+ rNrg4
CLP+Vehicle
CLP+ rNrg4
70 kDa
Marker
ACSL4
Sham+Vehicle
Sham+ rNrg4
CLP+Vehicle
CLP+ rNrg4
70 kDa
Marker
β-actin
Sham+Vehicle
Sham+ rNrg4
CLP+Vehicle
CLP+ rNrg4
55 kDa
40 kDa
35 kDa
25 kDa
15 kDa
Marker
β-actin
Sham+Vehicle
Sham+ rNrg4
CLP+Vehicle
CLP+ rNrg4
55 kDa
40 kDa
35 kDa
25 kDa
15 kDa

Figure 7j

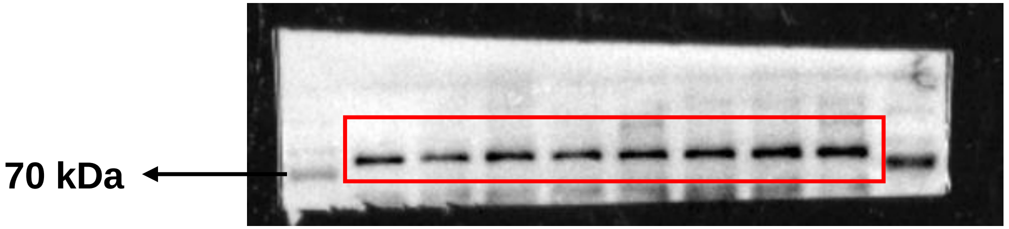
Marker
ACSL4
Sham+Vehicle
Sham+ rNrg4
CLP+Vehicle
CLP+ rNrg4
70 kDa

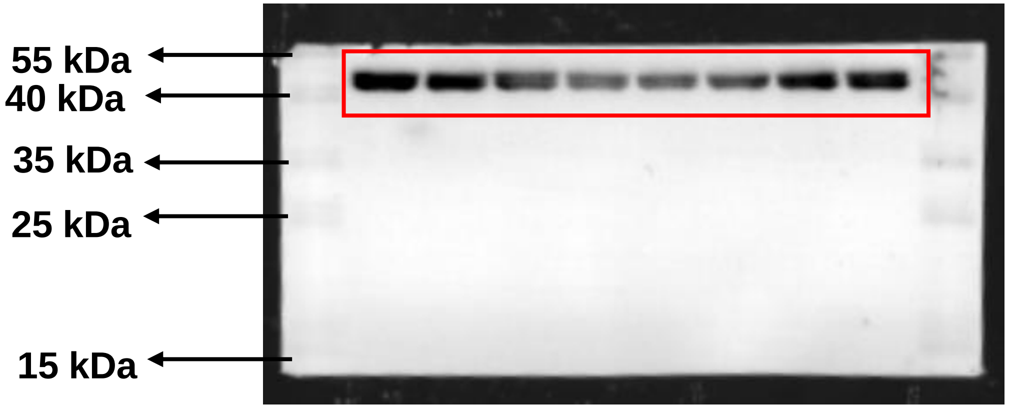
Marker
β-actin
Sham+Vehicle
Sham+ rNrg4
CLP+Vehicle
CLP+ rNrg4
55 kDa
40 kDa
35 kDa
25 kDa
15 kDa

Figure 7j

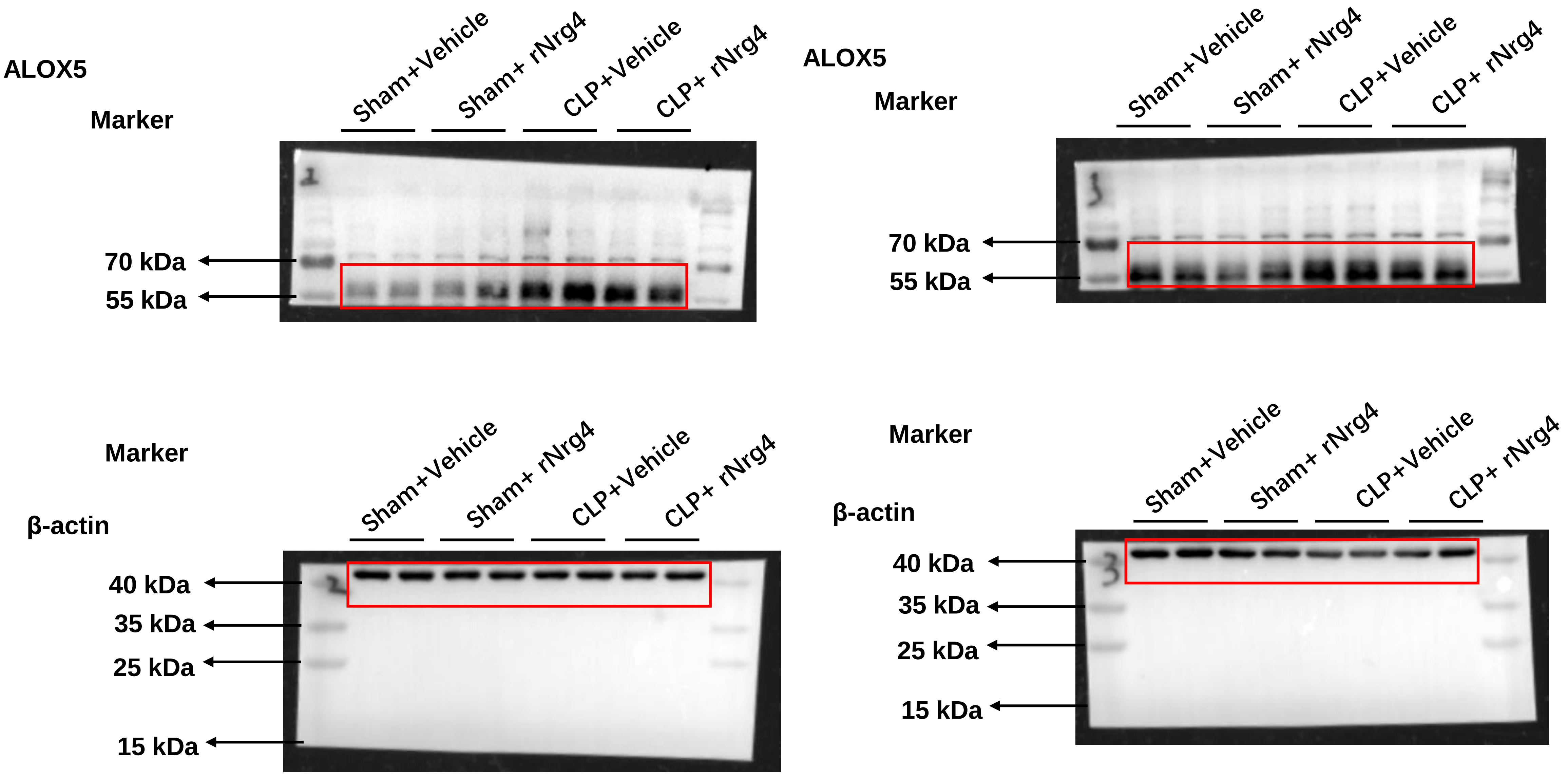
ALOX5
Marker
Sham+Vehicle
Sham+ rNrg4
CLP+Vehicle
CLP+ rNrg4
70 kDa
55 kDa
ALOX5
Marker
Sham+Vehicle
Sham+ rNrg4
CLP+Vehicle
CLP+ rNrg4
70 kDa
55 kDa
Marker
β-actin
Sham+Vehicle
Sham+ rNrg4
CLP+Vehicle
CLP+ rNrg4
40 kDa
35 kDa
25 kDa
15 kDa
Marker
β-actin
Sham+Vehicle
Sham+ rNrg4
CLP+Vehicle
CLP+ rNrg4
40 kDa
35 kDa
25 kDa
15 kDa

Figure 7j

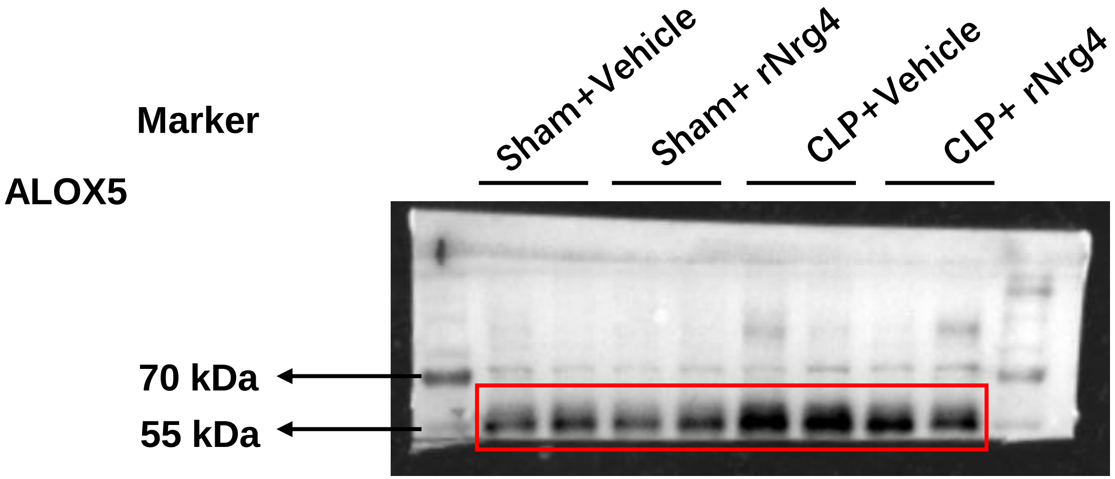

Marker
ALOX5
Sham+Vehicle
Sham+ rNrg4
CLP+Vehicle
CLP+ rNrg4
70 kDa
55 kDa

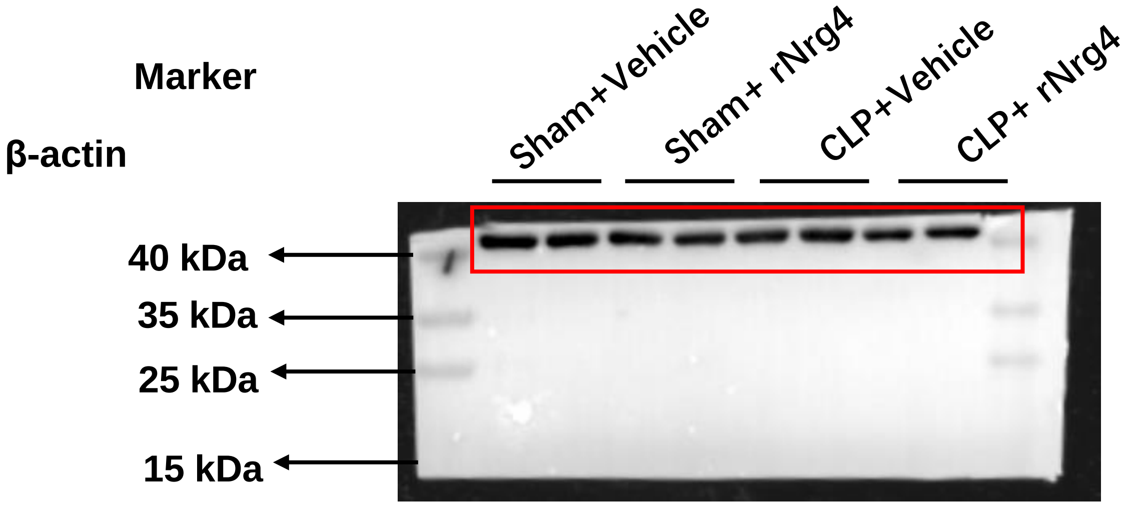

Marker
β-actin
Sham+Vehicle
Sham+ rNrg4
CLP+Vehicle
CLP+ rNrg4
40 kDa
35 kDa
25 kDa
15 kDa

Figure 7j

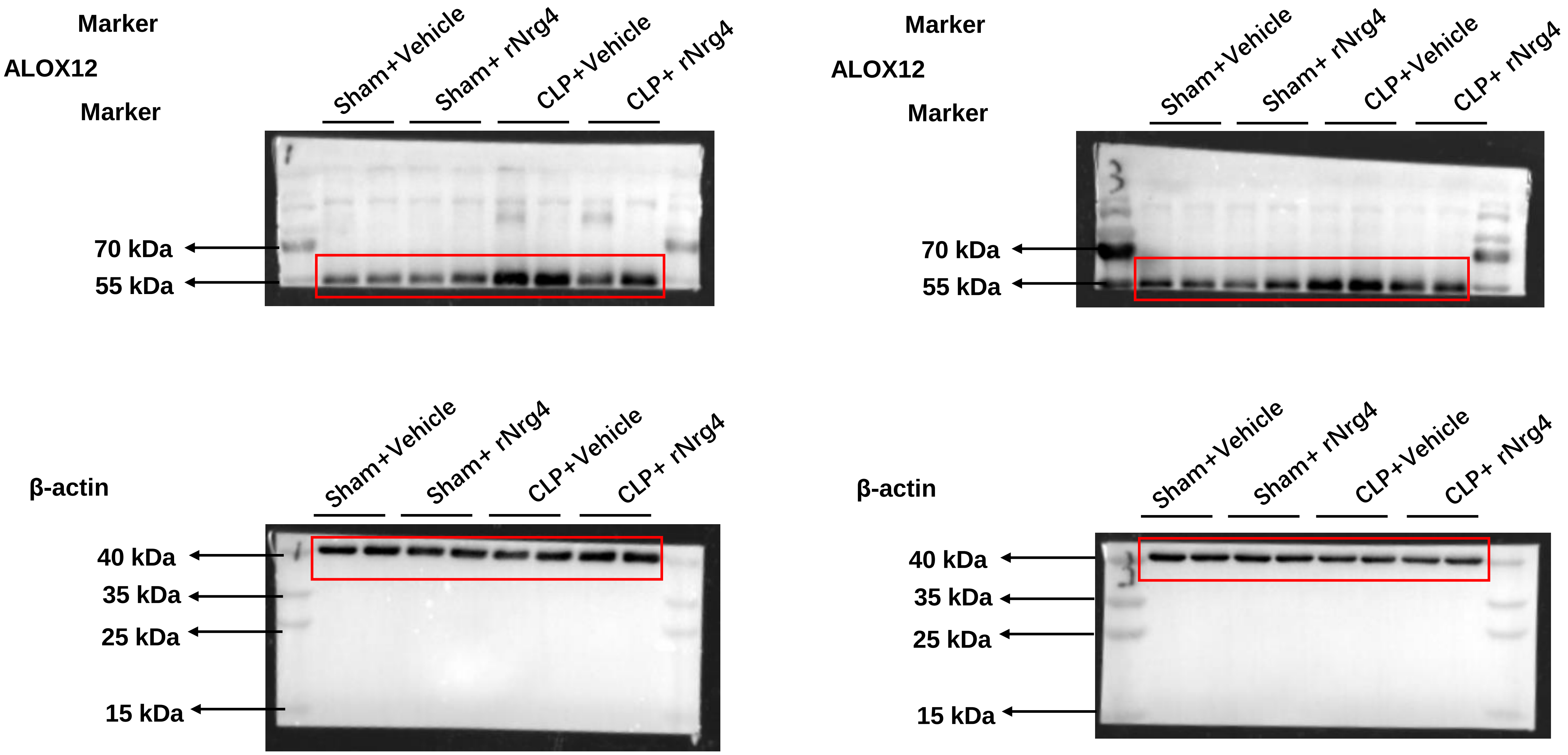
Marker
ALOX12
Marker
Sham+Vehicle
Sham+ rNrg4
CLP+Vehicle
CLP+ rNrg4
70 kDa
55 kDa
Marker
ALOX12
Marker
Sham+Vehicle
Sham+ rNrg4
CLP+Vehicle
CLP+ rNrg4
70 kDa
55 kDa
β-actin
Sham+Vehicle
Sham+ rNrg4
CLP+Vehicle
CLP+ rNrg4
40 kDa
35 kDa
25 kDa
15 kDa
β-actin
Sham+Vehicle
Sham+ rNrg4
CLP+Vehicle
CLP+ rNrg4
40 kDa
35 kDa
25 kDa
15 kDa

Figure 7j

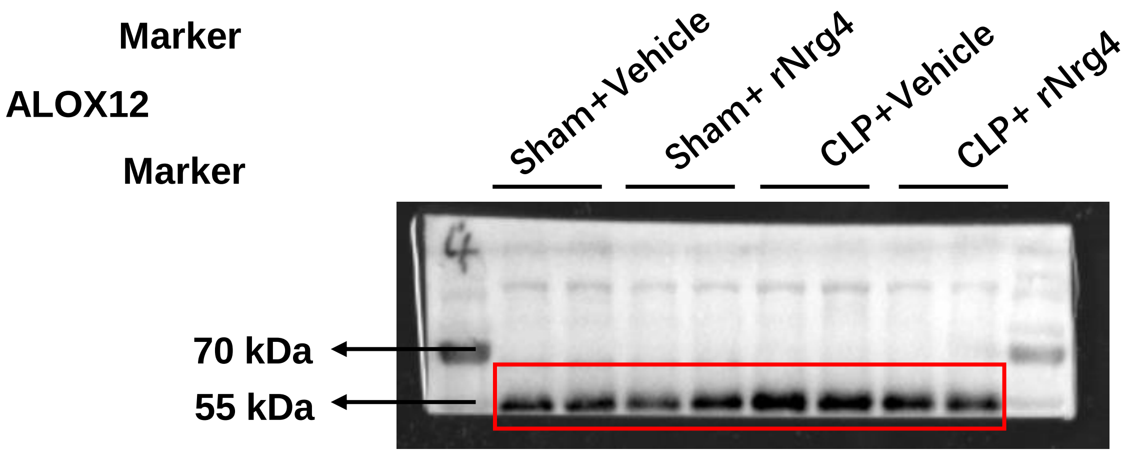
Marker
ALOX12
Marker
Sham+Vehicle
Sham+ rNrg4
CLP+Vehicle
CLP+ rNrg4
70 kDa
55 kDa

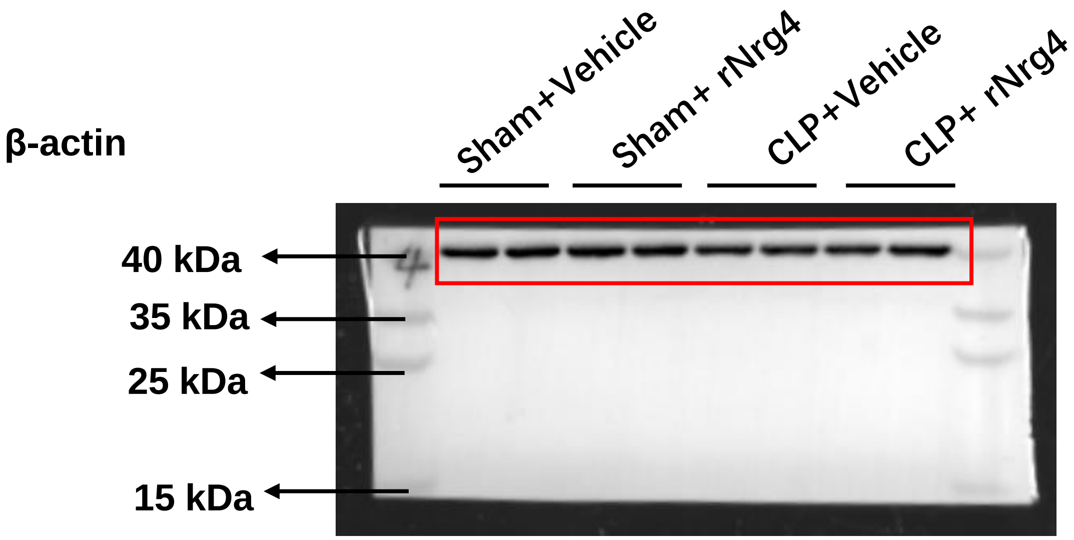
β-actin
Sham+Vehicle
Sham+ rNrg4
CLP+Vehicle
CLP+ rNrg4
40 kDa
35 kDa
25 kDa
15 kDa

Figure 7j

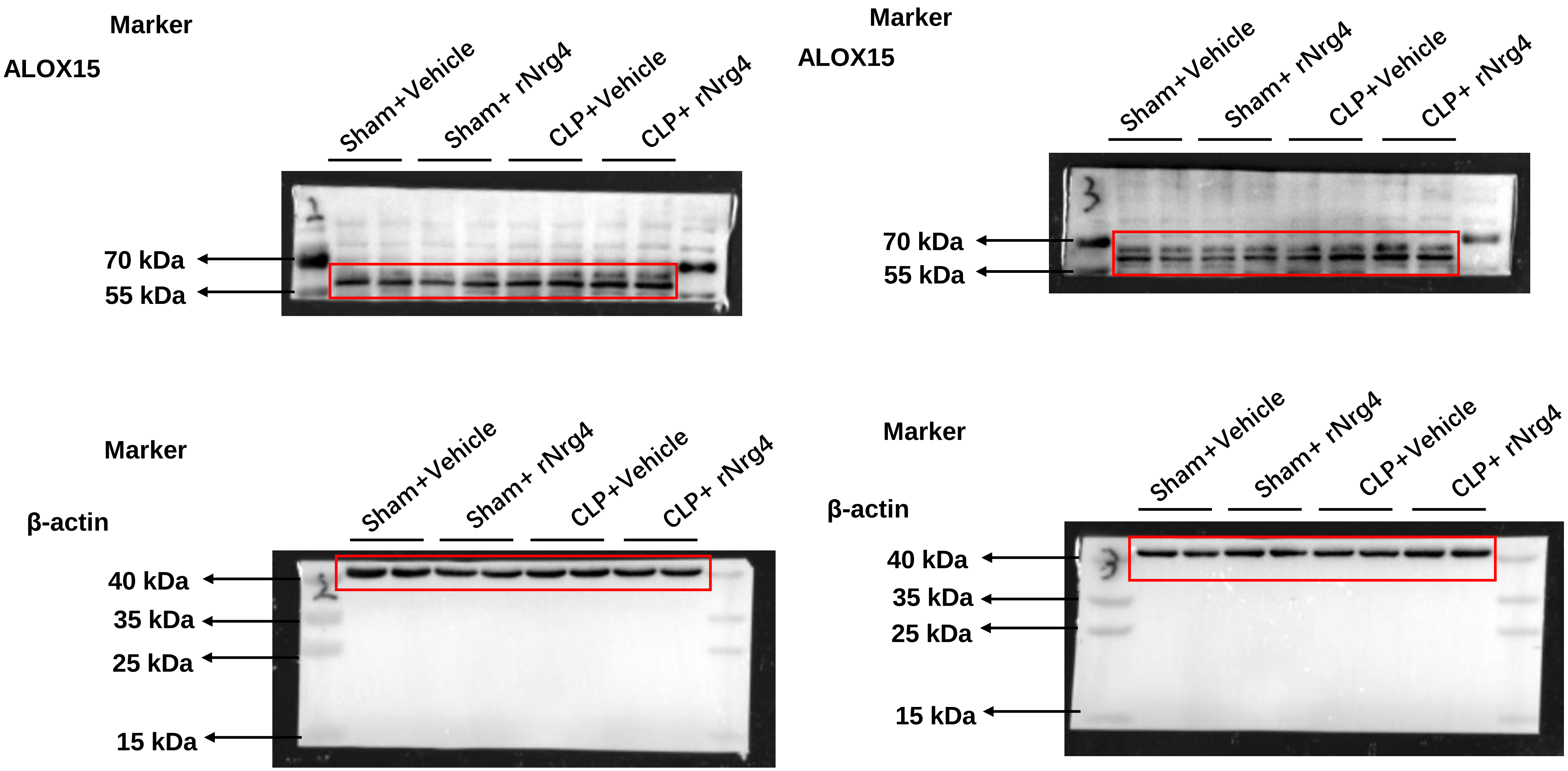

Marker
ALOX15
Sham+Vehicle
Sham+ rNrg4
CLP+Vehicle
CLP+ rNrg4
70 kDa
55 kDa
Marker
ALOX15
Sham+Vehicle
Sham+ rNrg4
CLP+Vehicle
CLP+ rNrg4
70 kDa
55 kDa
Marker
β-actin
Sham+Vehicle
Sham+ rNrg4
CLP+Vehicle
CLP+ rNrg4
40 kDa
35 kDa
25 kDa
15 kDa
Marker
β-actin
Sham+Vehicle
Sham+ rNrg4
CLP+Vehicle
CLP+ rNrg4
40 kDa
35 kDa
25 kDa
15 kDa

Figure 7j

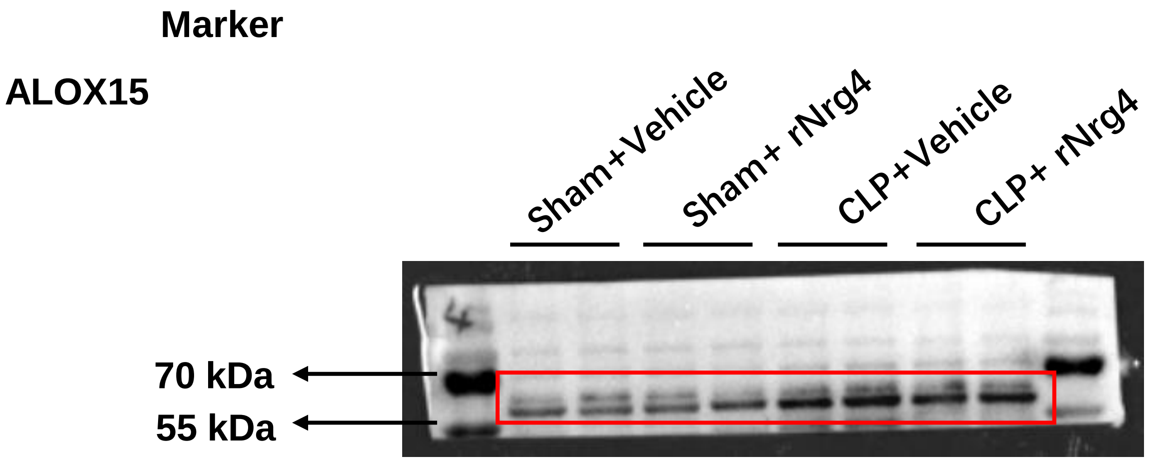
Marker
ALOX15
Sham+Vehicle
Sham+ rNrg4
CLP+Vehicle
CLP+ rNrg4
70 kDa
55 kDa

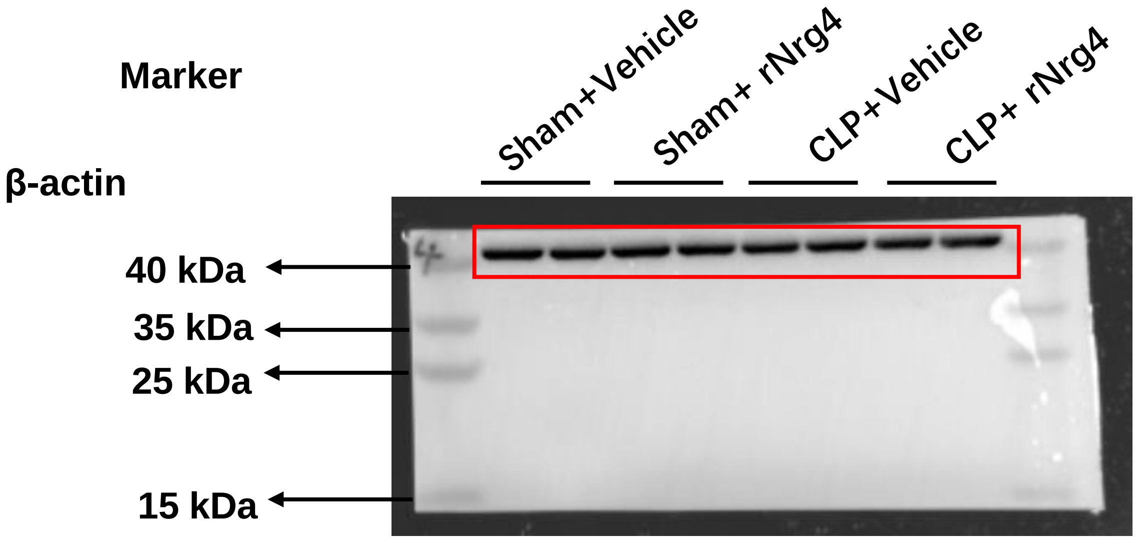
Marker
β-actin
Sham+Vehicle
Sham+ rNrg4
CLP+Vehicle
CLP+ rNrg4
40 kDa
35 kDa
25 kDa
15 kDa

Figure 7j

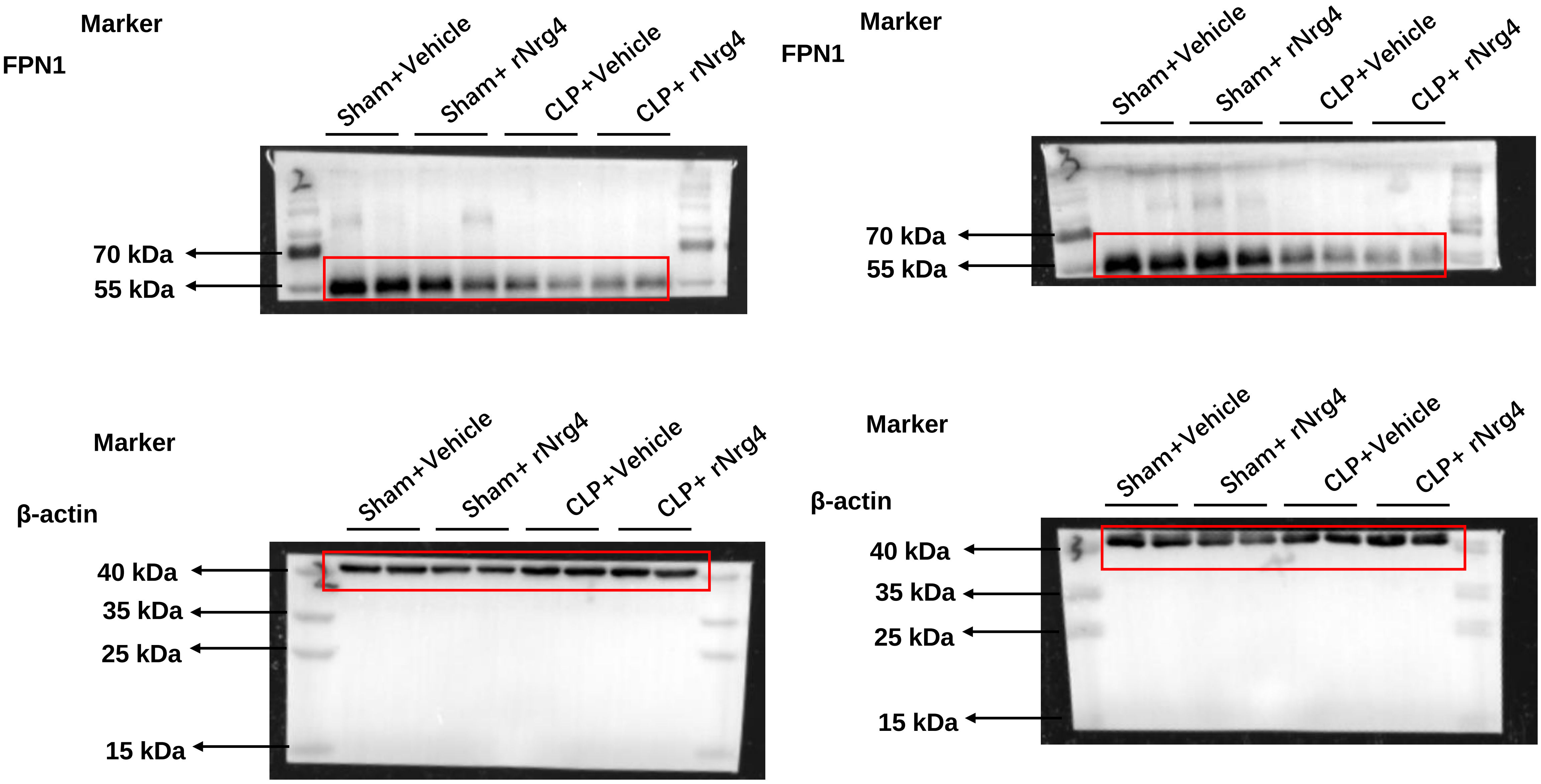
Marker
FPN1
Sham+Vehicle
Sham+ rNrg4
CLP+Vehicle
CLP+ rNrg4
70 kDa
55 kDa
Marker
FPN1
Sham+Vehicle
Sham+ rNrg4
CLP+Vehicle
CLP+ rNrg4
70 kDa
55 kDa
Marker
β-actin
Sham+Vehicle
Sham+ rNrg4
CLP+Vehicle
CLP+ rNrg4
40 kDa
35 kDa
25 kDa
15 kDa
Marker
β-actin
Sham+Vehicle
Sham+ rNrg4
CLP+Vehicle
CLP+ rNrg4
40 kDa
35 kDa
25 kDa
15 kDa

Figure 7j

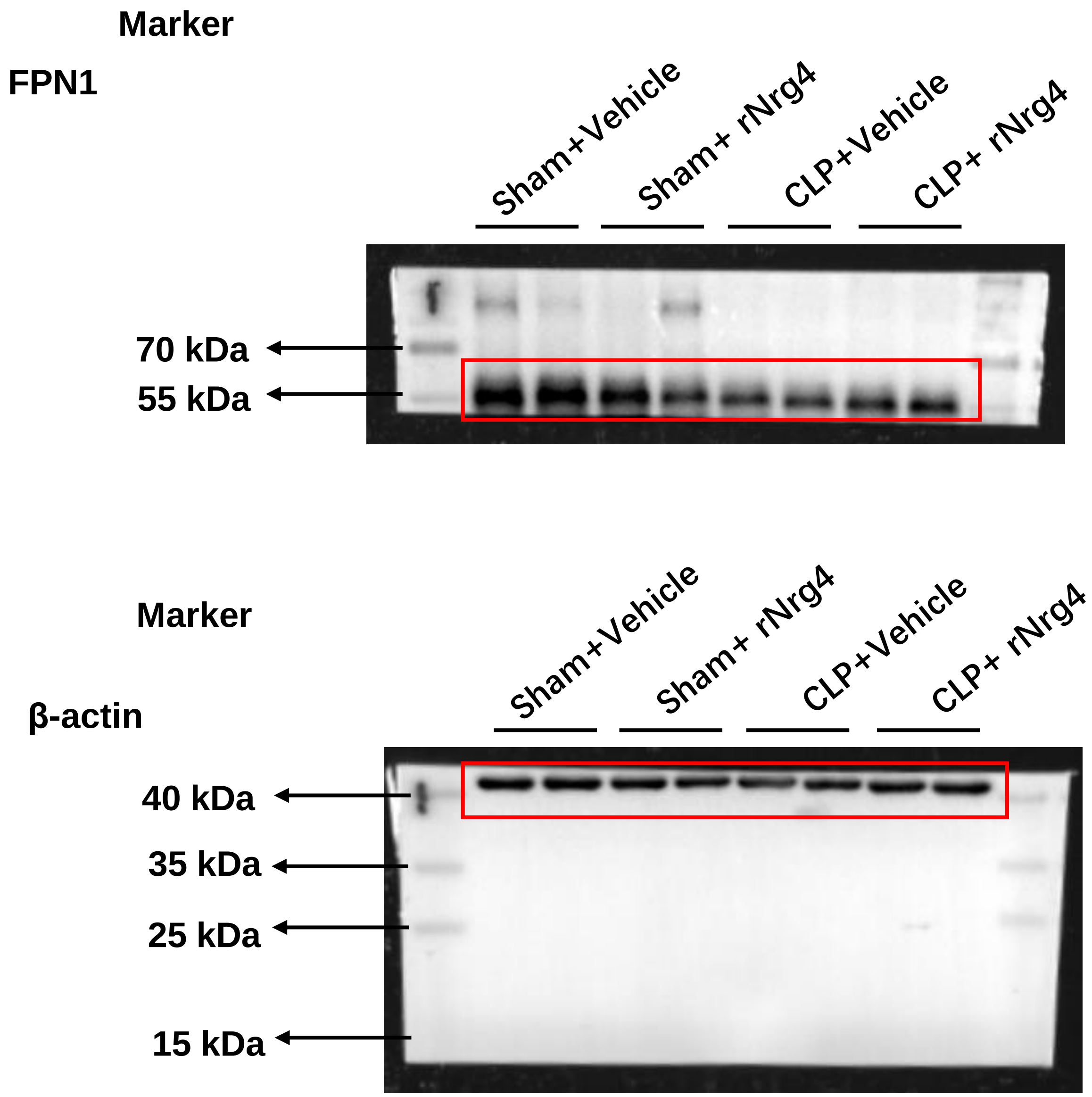
Marker
FPN1
Sham+Vehicle
Sham+ rNrg4
CLP+Vehicle
CLP+ rNrg4
70 kDa
55 kDa
Marker
β-actin
Sham+Vehicle
Sham+ rNrg4
CLP+Vehicle
CLP+ rNrg4
40 kDa
35 kDa
25 kDa
15 kDa

Figure 7j

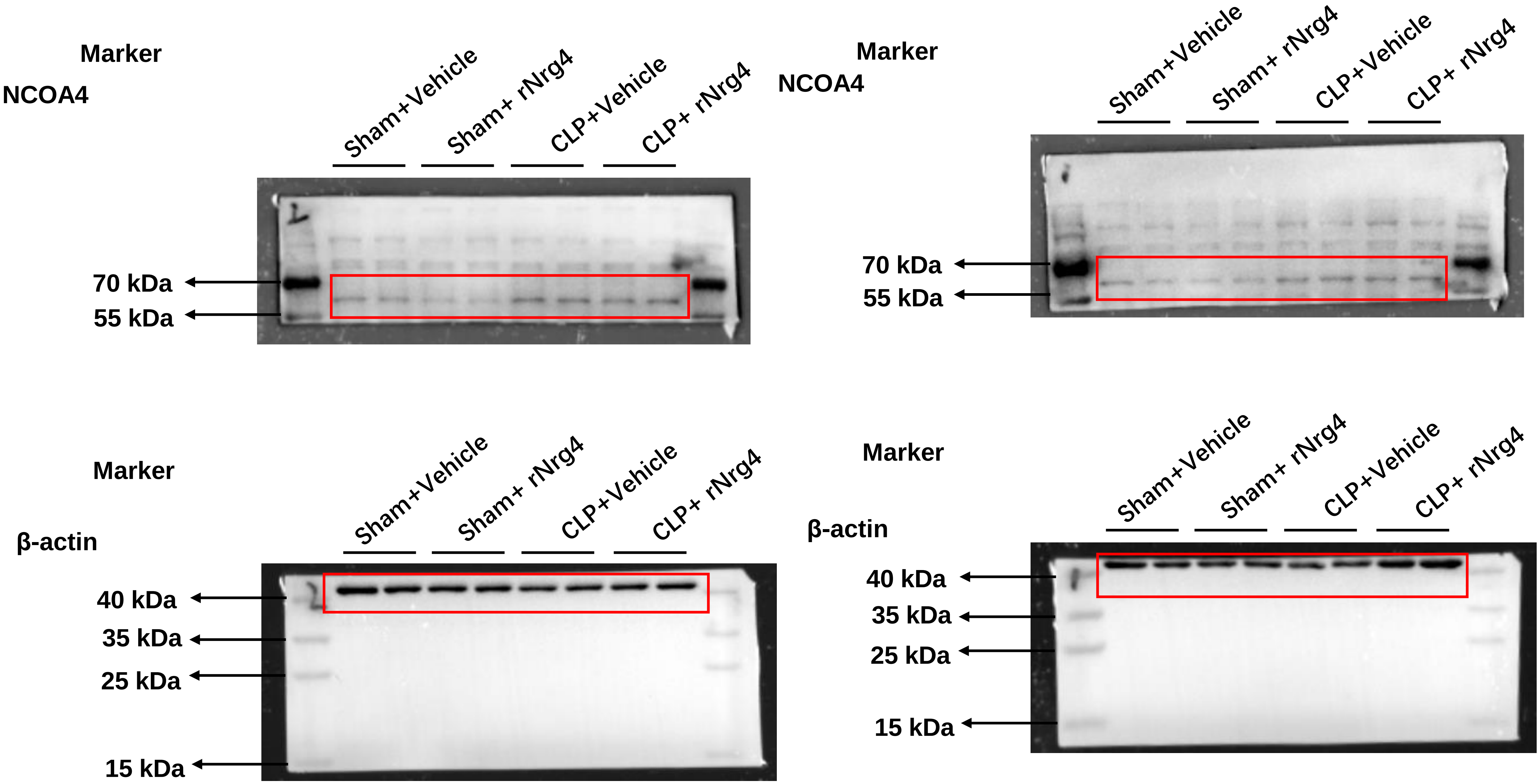

Marker
NCOA4
Sham+Vehicle
Sham+ rNrg4
CLP+Vehicle
CLP+ rNrg4
70 kDa
55 kDa
Marker
NCOA4
Sham+Vehicle
Sham+ rNrg4
CLP+Vehicle
CLP+ rNrg4
70 kDa
55 kDa
Marker
β-actin
Sham+Vehicle
Sham+ rNrg4
CLP+Vehicle
CLP+ rNrg4
40 kDa
35 kDa
25 kDa
15 kDa
Marker
β-actin
Sham+Vehicle
Sham+ rNrg4
CLP+Vehicle
CLP+ rNrg4
40 kDa
35 kDa
25 kDa
15 kDa

Figure 7j

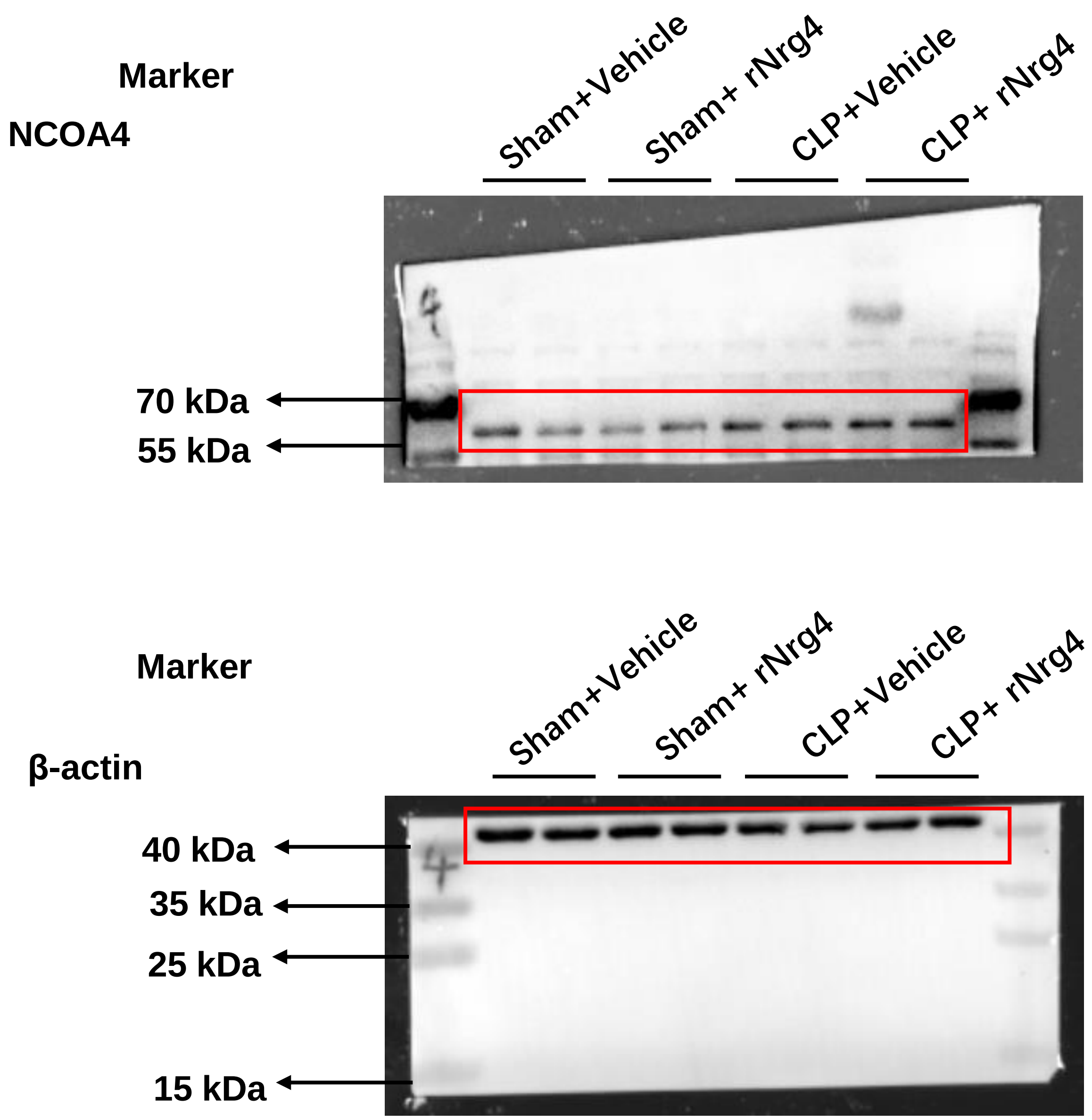

Marker
NCOA4
Sham+Vehicle
Sham+ rNrg4
CLP+Vehicle
CLP+ rNrg4
70 kDa
55 kDa
Marker
β-actin
Sham+Vehicle
Sham+ rNrg4
CLP+Vehicle
CLP+ rNrg4
40 kDa
35 kDa
25 kDa
15 kDa

Figure 7j

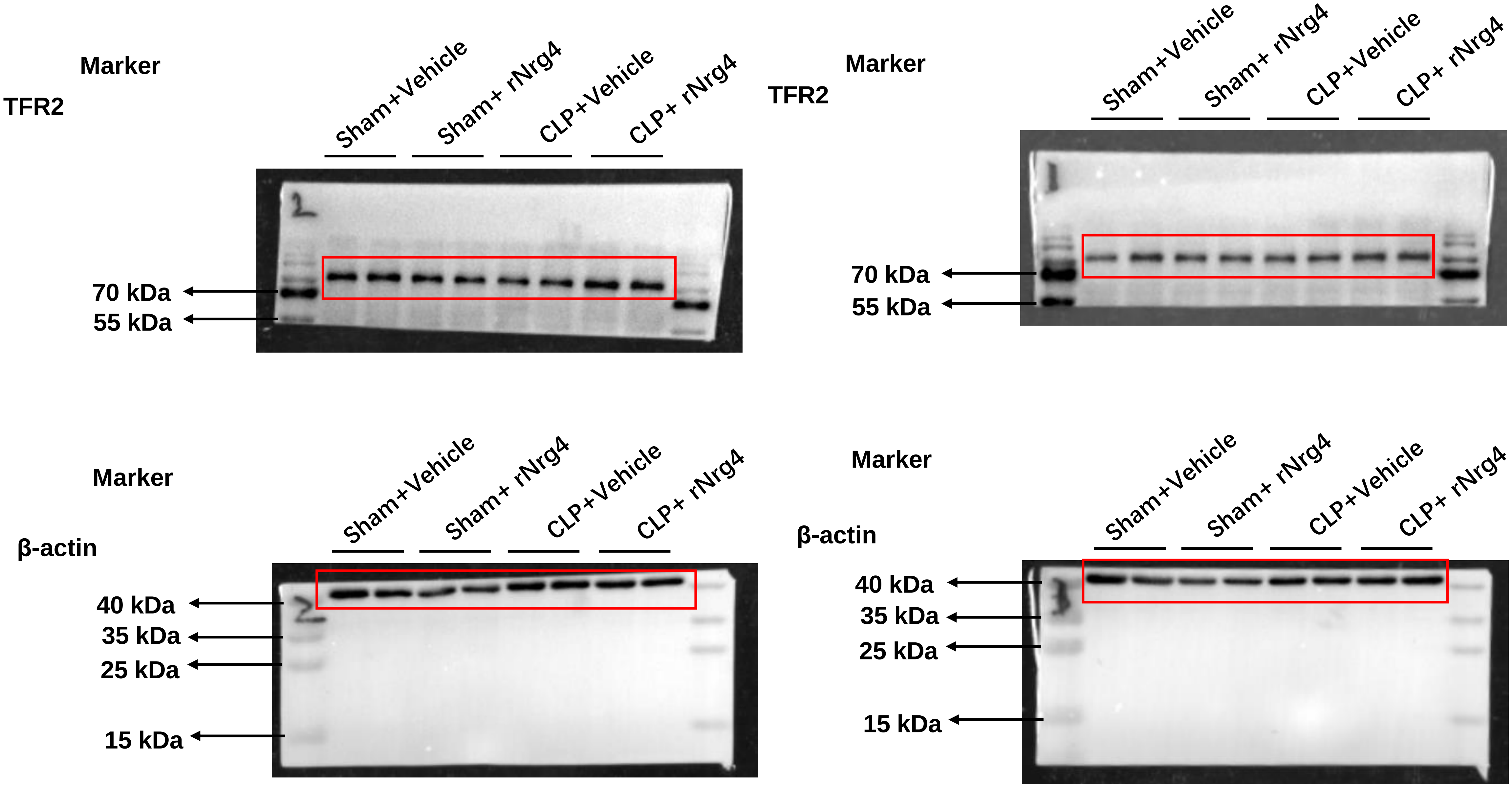

Marker
TFR2
Sham+Vehicle
Sham+ rNrg4
CLP+Vehicle
CLP+ rNrg4
70 kDa
55 kDa
Marker
TFR2
Sham+Vehicle
Sham+ rNrg4
CLP+Vehicle
CLP+ rNrg4
70 kDa
55 kDa
Marker
β-actin
Sham+Vehicle
Sham+ rNrg4
CLP+Vehicle
CLP+ rNrg4
40 kDa
35 kDa
25 kDa
15 kDa
Marker
β-actin
Sham+Vehicle
Sham+ rNrg4
CLP+Vehicle
CLP+ rNrg4
40 kDa
35 kDa
25 kDa
15 kDa

Figure 7j

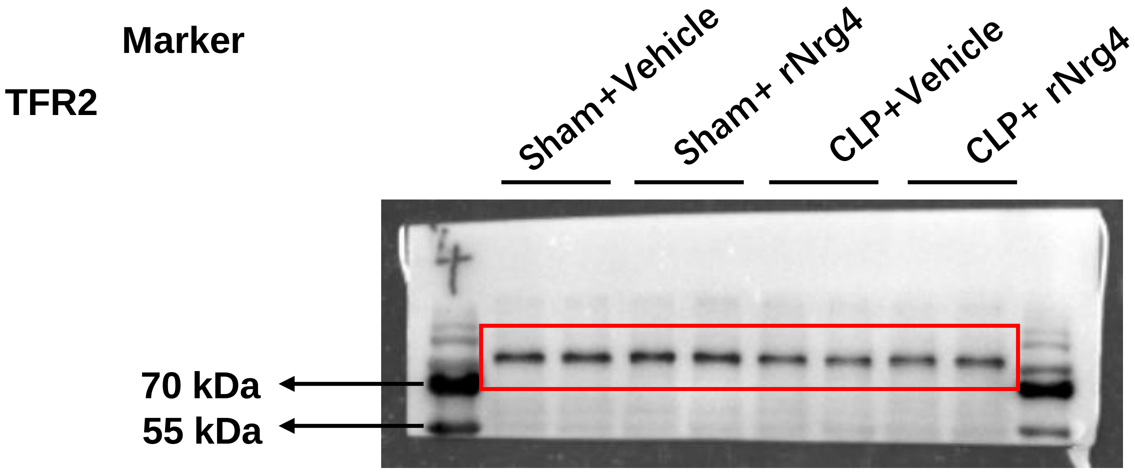
Marker
TFR2
Sham+Vehicle
Sham+ rNrg4
CLP+Vehicle
CLP+ rNrg4
70 kDa
55 kDa

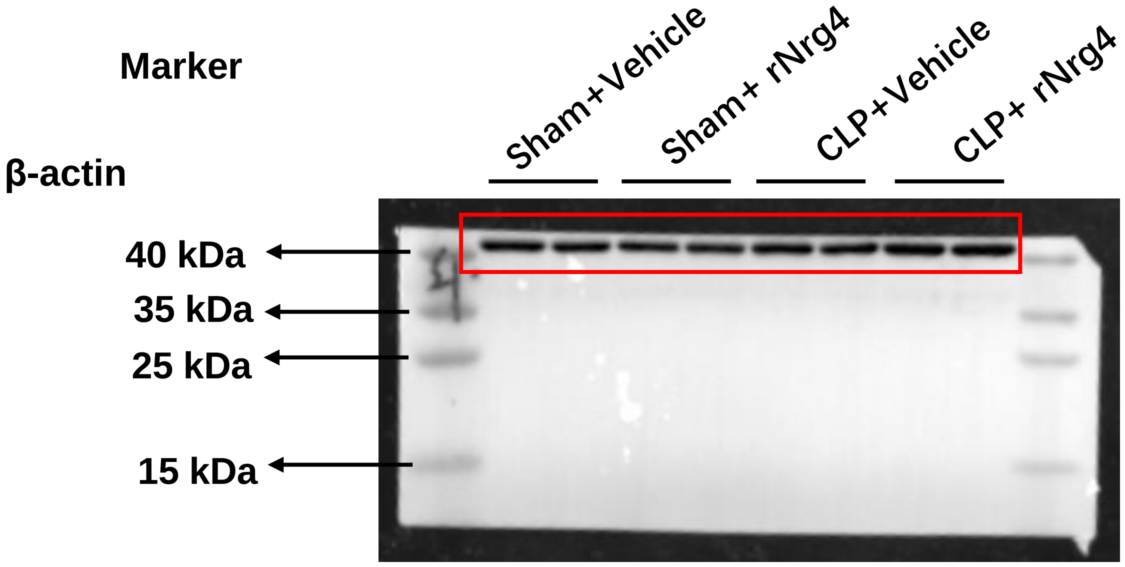
Marker
β-actin
Sham+Vehicle
Sham+ rNrg4
CLP+Vehicle
CLP+ rNrg4
40 kDa
35 kDa
25 kDa
15 kDa

Figure 7i

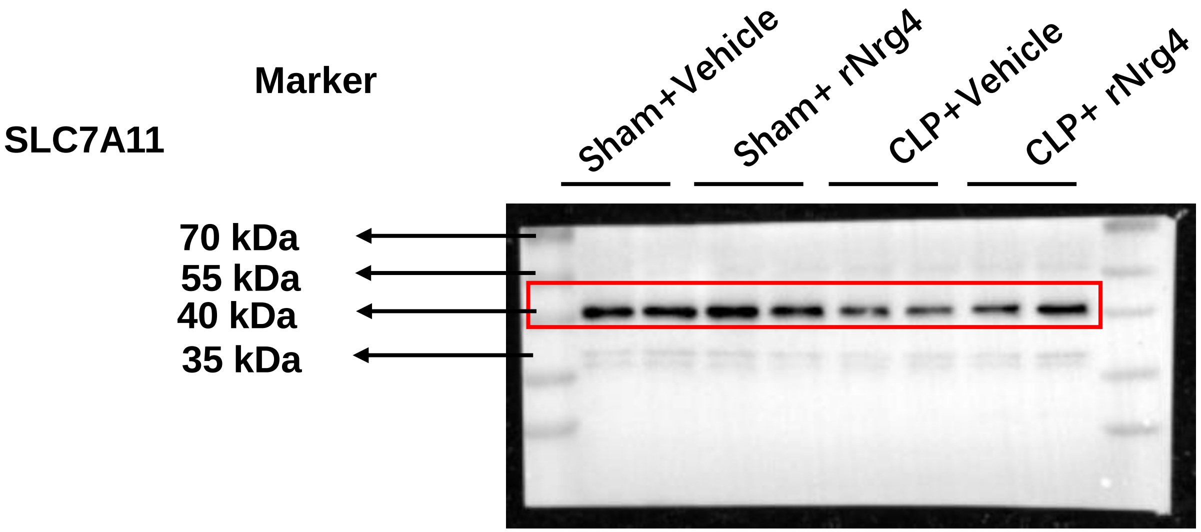

SLC7A11
Marker
Sham+Vehicle
Sham+ rNrg4
CLP+Vehicle
CLP+ rNrg4
70 kDa
55 kDa
40 kDa
35 kDa

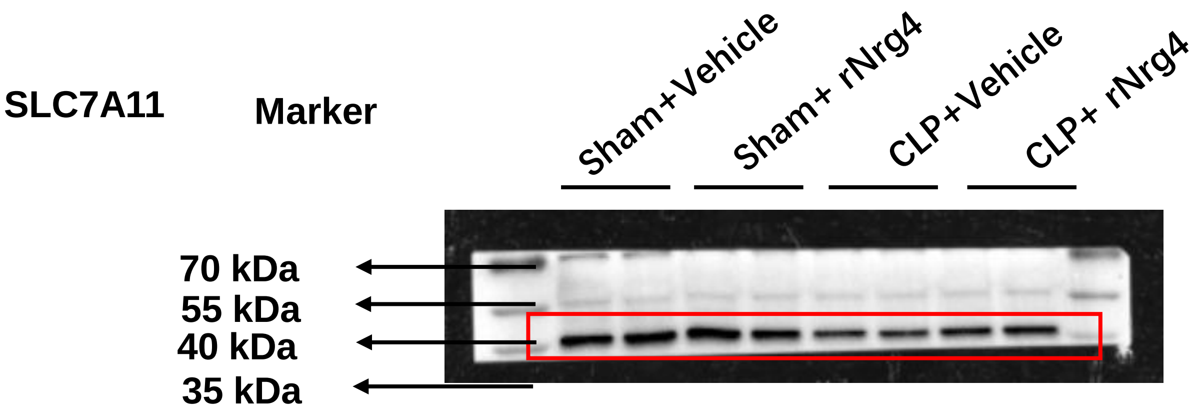

SLC7A11
Marker
Sham+Vehicle
Sham+ rNrg4
CLP+Vehicle
CLP+ rNrg4
70 kDa
55 kDa
40 kDa
35 kDa

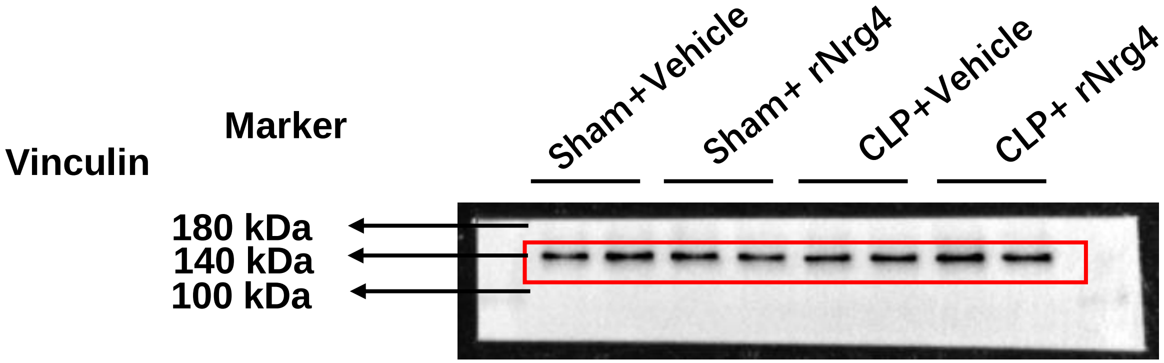

Vinculin
Marker
Sham+Vehicle
Sham+ rNrg4
CLP+Vehicle
CLP+ rNrg4
180 kDa
140 kDa
100 kDa

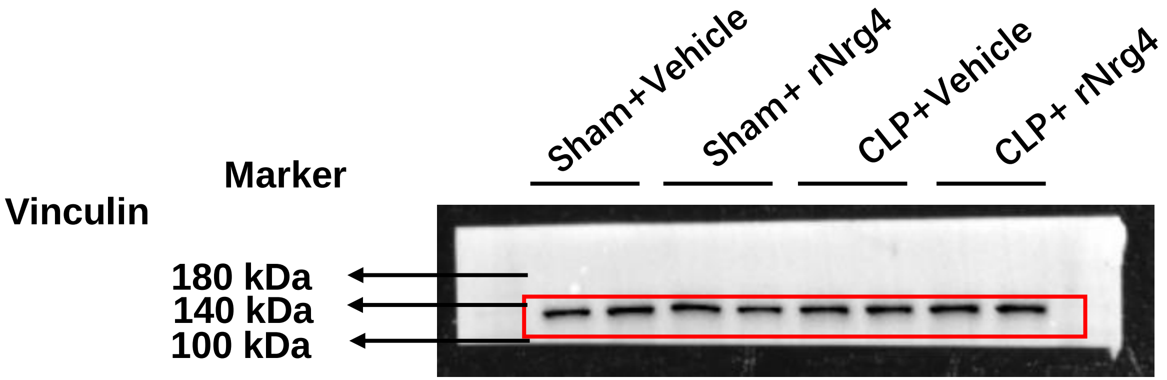

Vinculin
Marker
Sham+Vehicle
Sham+ rNrg4
CLP+Vehicle
CLP+ rNrg4
180 kDa
140 kDa
100 kDa

Figure 7i

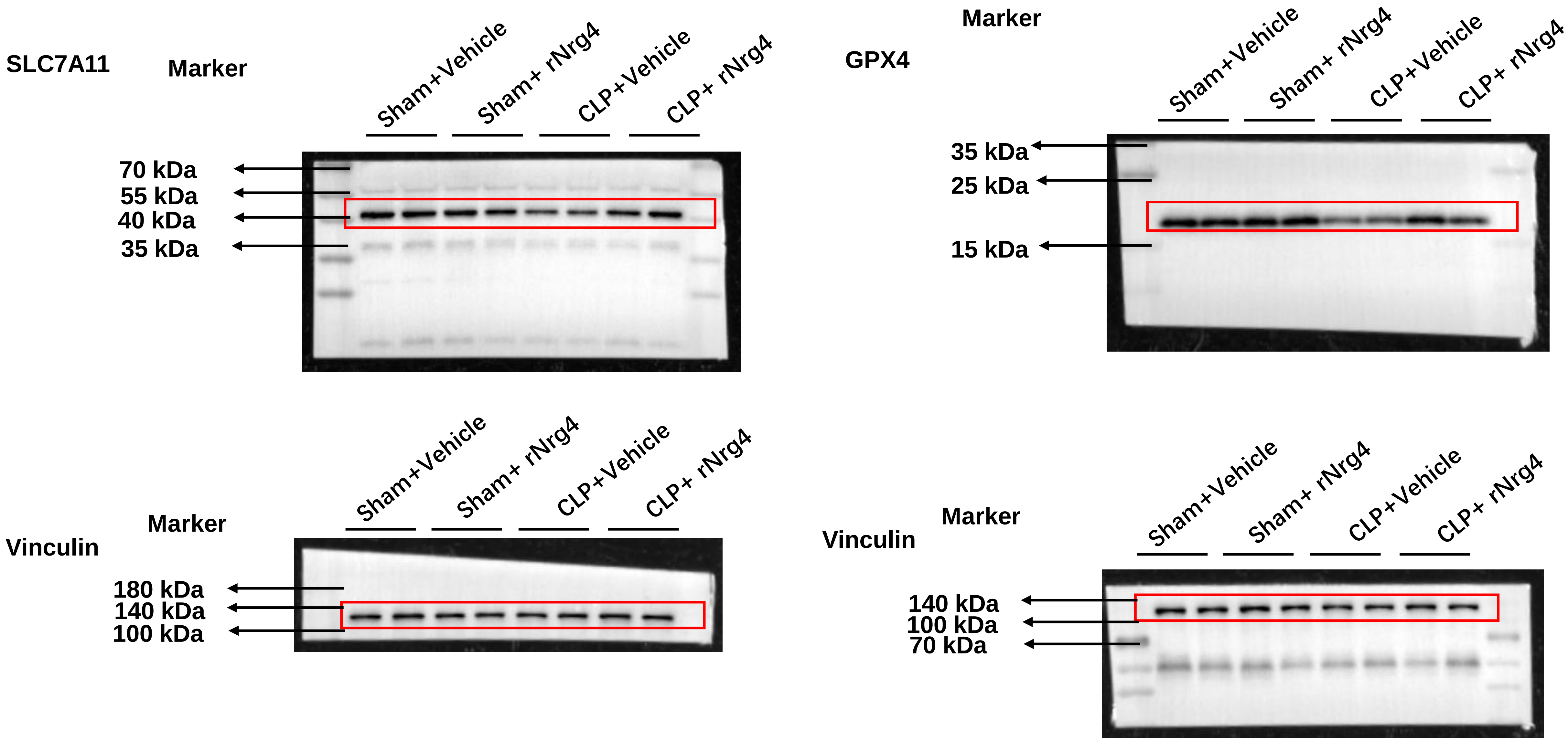
SLC7A11
Marker
Sham+Vehicle
Sham+ rNrg4
CLP+Vehicle
CLP+ rNrg4
70 kDa
55 kDa
40 kDa
35 kDa
GPX4
Marker
Sham+Vehicle
Sham+ rNrg4
CLP+Vehicle
CLP+ rNrg4
35 kDa
25 kDa
15 kDa
Vinculin
Marker
Sham+Vehicle
Sham+ rNrg4
CLP+Vehicle
CLP+ rNrg4
180 kDa
140 kDa
100 kDa
Vinculin
Marker
Sham+Vehicle
Sham+ rNrg4
CLP+Vehicle
CLP+ rNrg4
140 kDa
100 kDa
70 kDa

Figure 7i

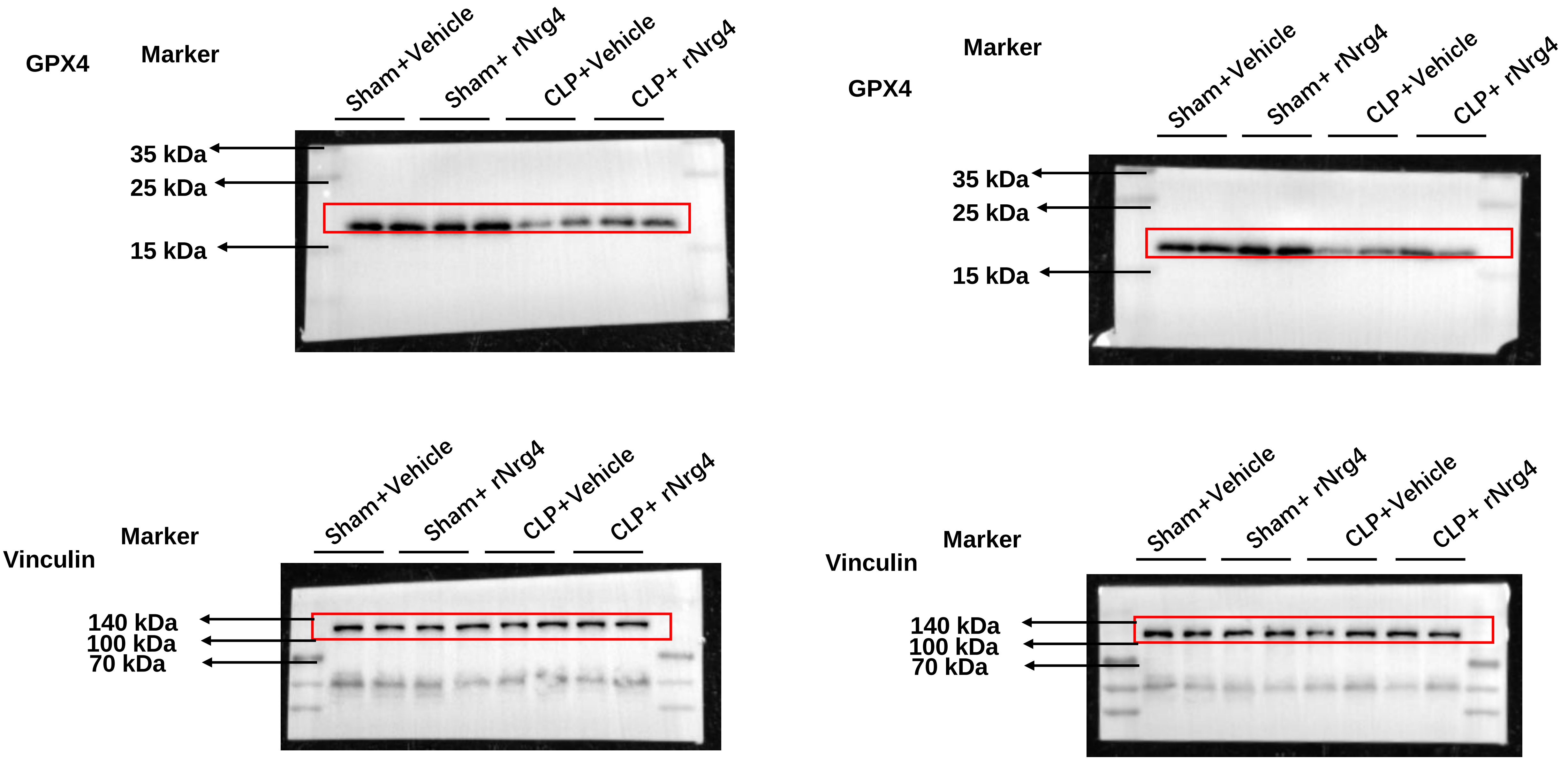
GPX4
Marker
Sham+Vehicle
Sham+ rNrg4
CLP+Vehicle
CLP+ rNrg4
35 kDa
25 kDa
15 kDa
Marker
GPX4
Sham+Vehicle
Sham+ rNrg4
CLP+Vehicle
CLP+ rNrg4
35 kDa
25 kDa
15 kDa
Marker
Vinculin
Sham+Vehicle
Sham+ rNrg4
CLP+Vehicle
CLP+ rNrg4
140 kDa
100 kDa
70 kDa
Marker
Vinculin
Sham+Vehicle
Sham+ rNrg4
CLP+Vehicle
CLP+ rNrg4
140 kDa
100 kDa
70 kDa

Figure 8c

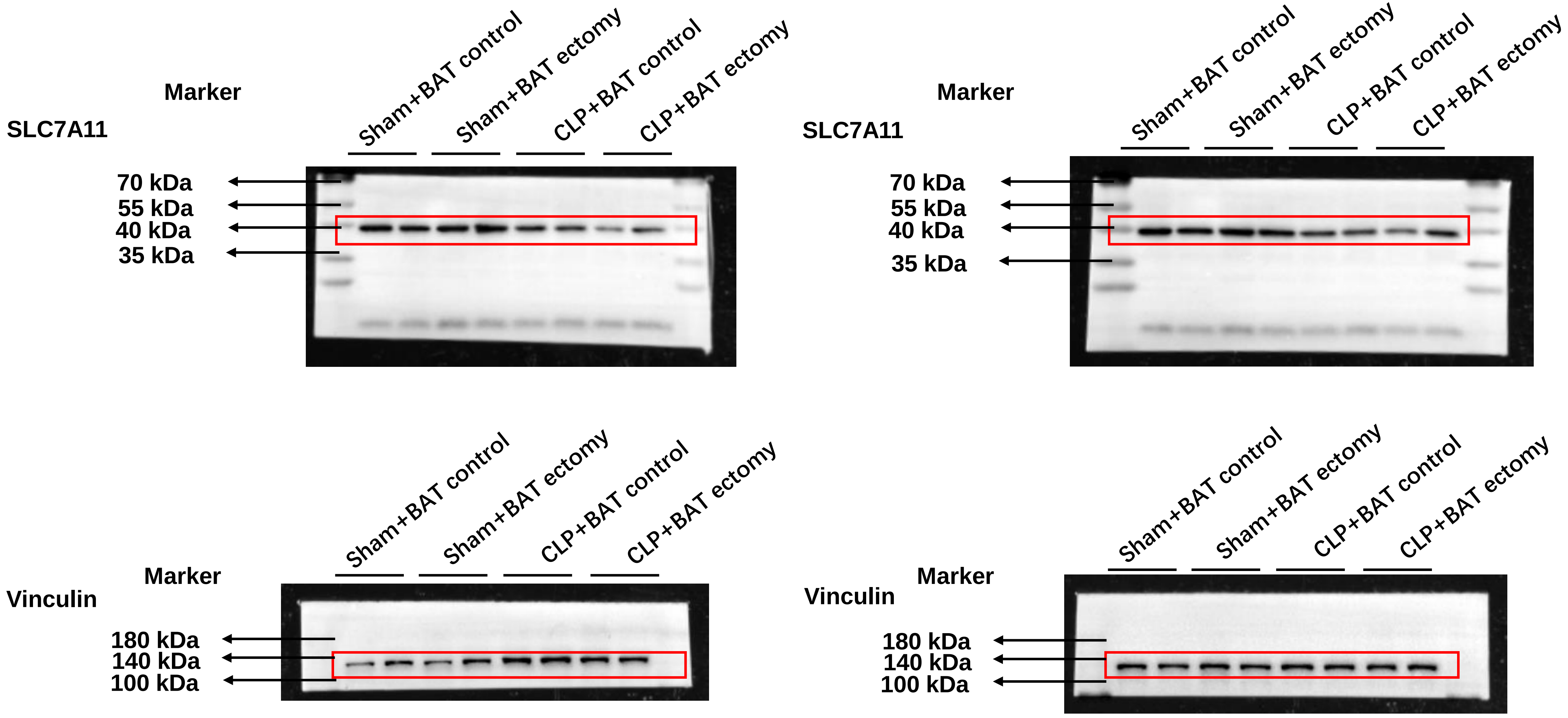
SLC7A11
Marker
Sham+BAT control
Sham+BAT ectomy
CLP+BAT control
CLP+BAT ectomy
70 kDa
55 kDa
40 kDa
35 kDa
SLC7A11
Marker
Sham+BAT control
Sham+BAT ectomy
CLP+BAT control
CLP+BAT ectomy
70 kDa
55 kDa
40 kDa
35 kDa
Vinculin
Marker
Sham+BAT control
Sham+BAT ectomy
CLP+BAT control
CLP+BAT ectomy
180 kDa
140 kDa
100 kDa
Vinculin
Marker
Sham+BAT control
Sham+BAT ectomy
CLP+BAT control
CLP+BAT ectomy
180 kDa
140 kDa
100 kDa

Figure 8c

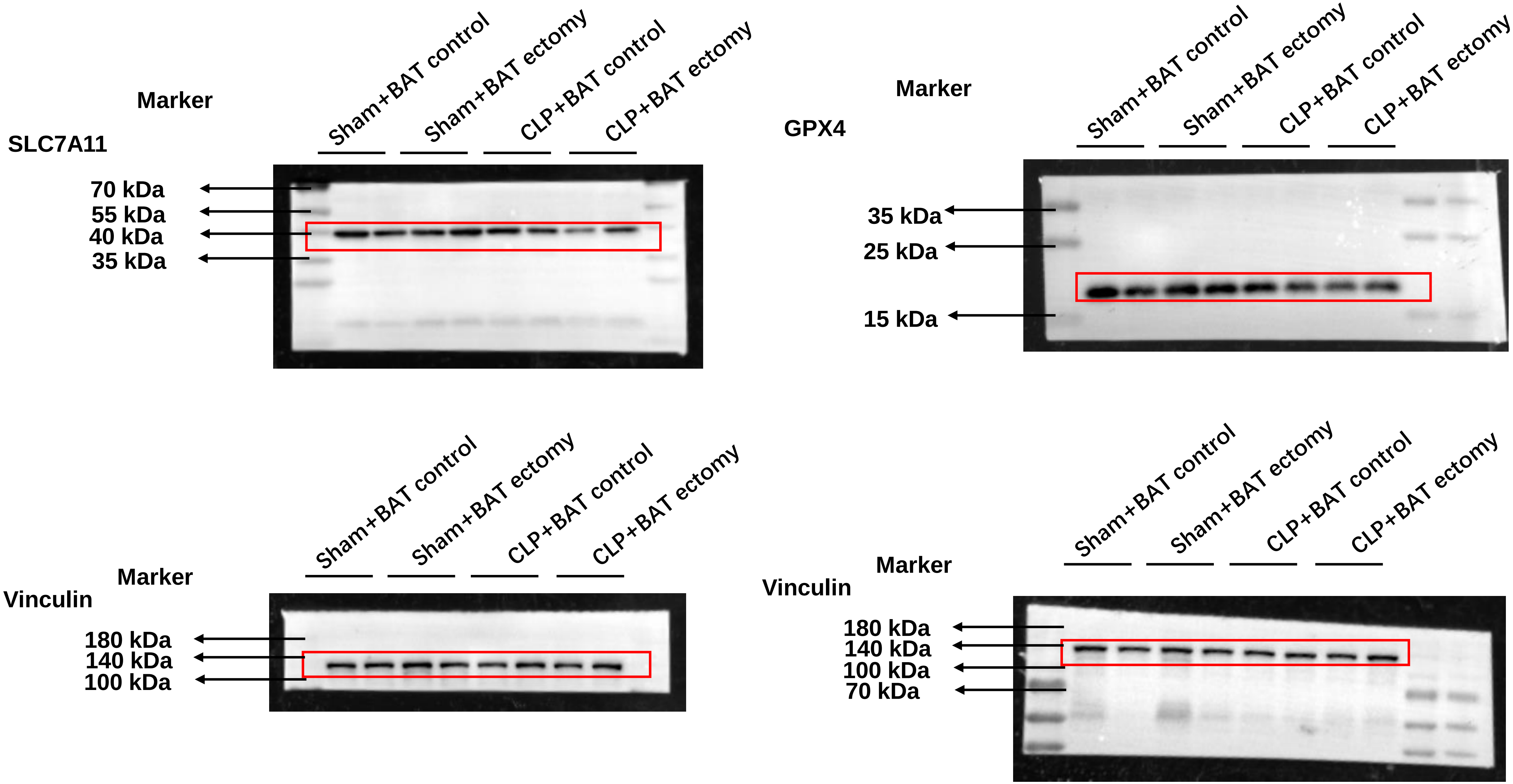

SLC7A11
Marker
Sham+BAT control
Sham+BAT ectomy
CLP+BAT control
CLP+BAT ectomy
70 kDa
55 kDa
40 kDa
35 kDa
GPX4
Marker
Sham+BAT control
Sham+BAT ectomy
CLP+BAT control
CLP+BAT ectomy
35 kDa
25 kDa
15 kDa
Vinculin
Marker
Sham+BAT control
Sham+BAT ectomy
CLP+BAT control
CLP+BAT ectomy
180 kDa
140 kDa
100 kDa
Vinculin
Marker
Sham+BAT control
Sham+BAT ectomy
CLP+BAT control
CLP+BAT ectomy
180 kDa
140 kDa
100 kDa
70 kDa

Figure 8c

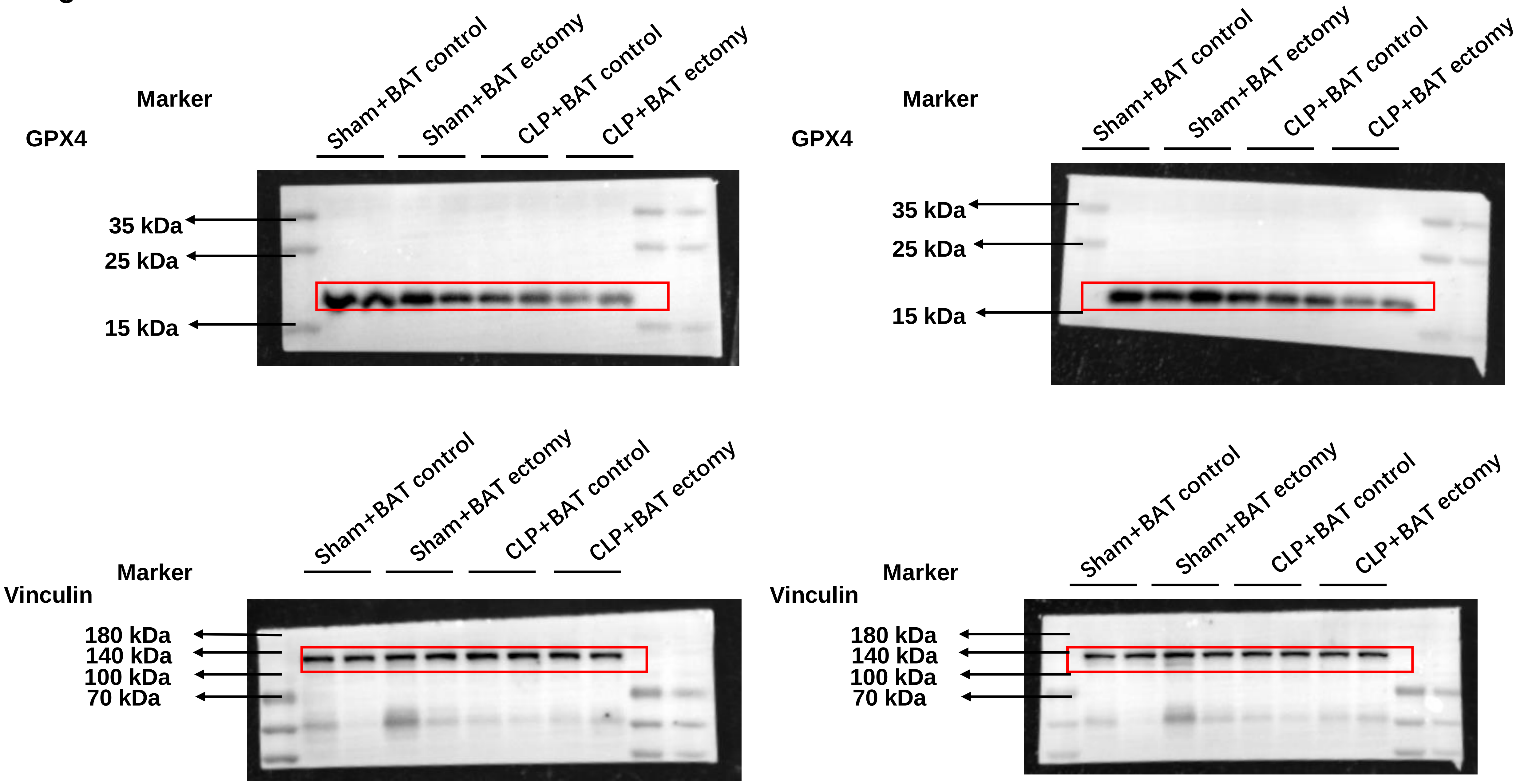

Marker
GPX4
Sham+BAT control
Sham+BAT ectomy
CLP+BAT control
CLP+BAT ectomy
35 kDa
25 kDa
15 kDa
Marker
GPX4
Sham+BAT control
Sham+BAT ectomy
CLP+BAT control
CLP+BAT ectomy
35 kDa
25 kDa
15 kDa
Marker
Vinculin
Sham+BAT control
Sham+BAT ectomy
CLP+BAT control
CLP+BAT ectomy
180 kDa
140 kDa
100 kDa
70 kDa
Marker
Vinculin
Sham+BAT control
Sham+BAT ectomy
CLP+BAT control
CLP+BAT ectomy
180 kDa
140 kDa
100 kDa
70 kDa

Figure 8f

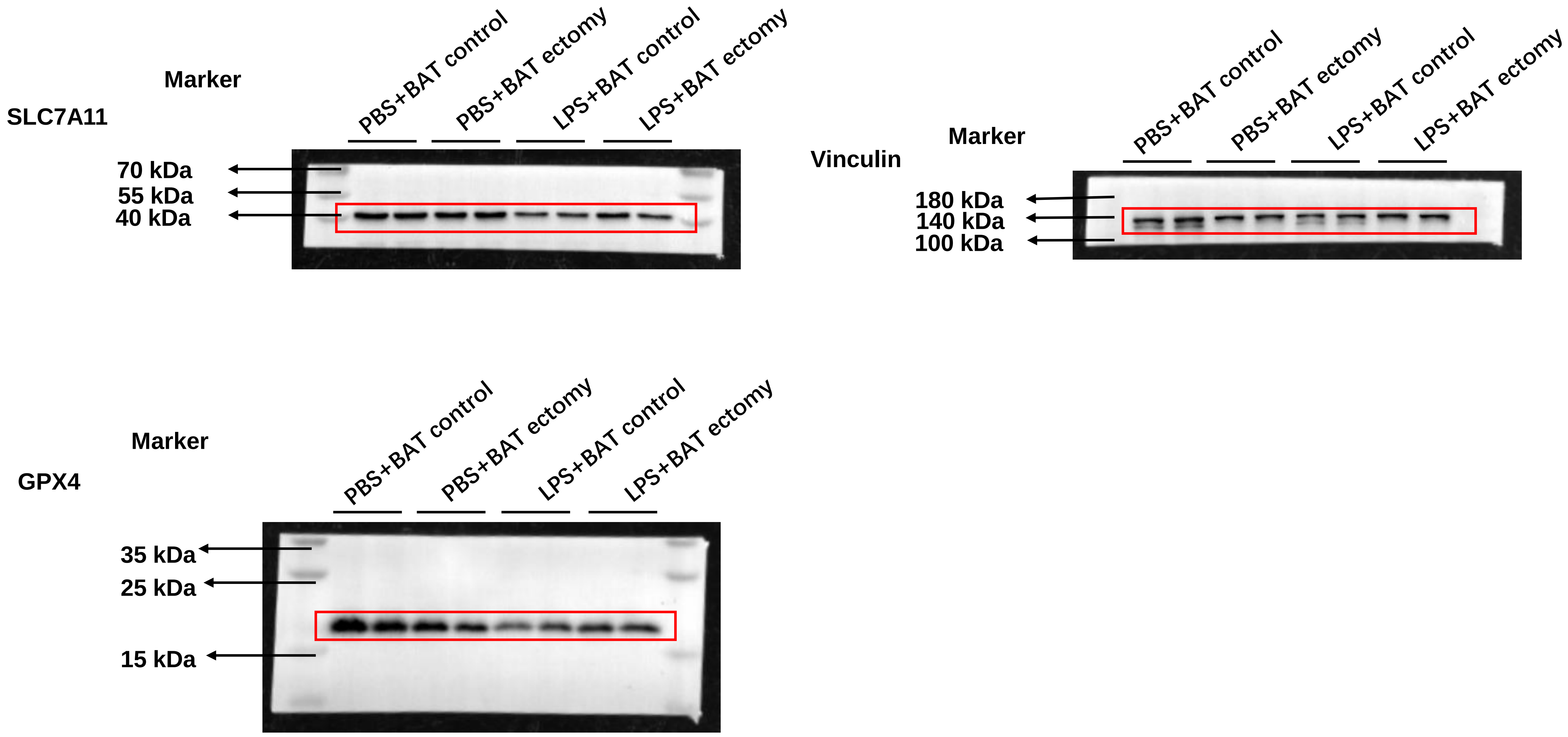
SLC7A11
Marker
PBS+BAT control
PBS+BAT ectomy
LPS+BAT control
LPS+BAT ectomy
70 kDa
55 kDa
40 kDa
Vinculin
Marker
PBS+BAT control
PBS+BAT ectomy
LPS+BAT control
LPS+BAT ectomy
180 kDa
140 kDa
100 kDa
GPX4
Marker
PBS+BAT control
PBS+BAT ectomy
LPS+BAT control
LPS+BAT ectomy
35 kDa
25 kDa
15 kDa

Figure 8f

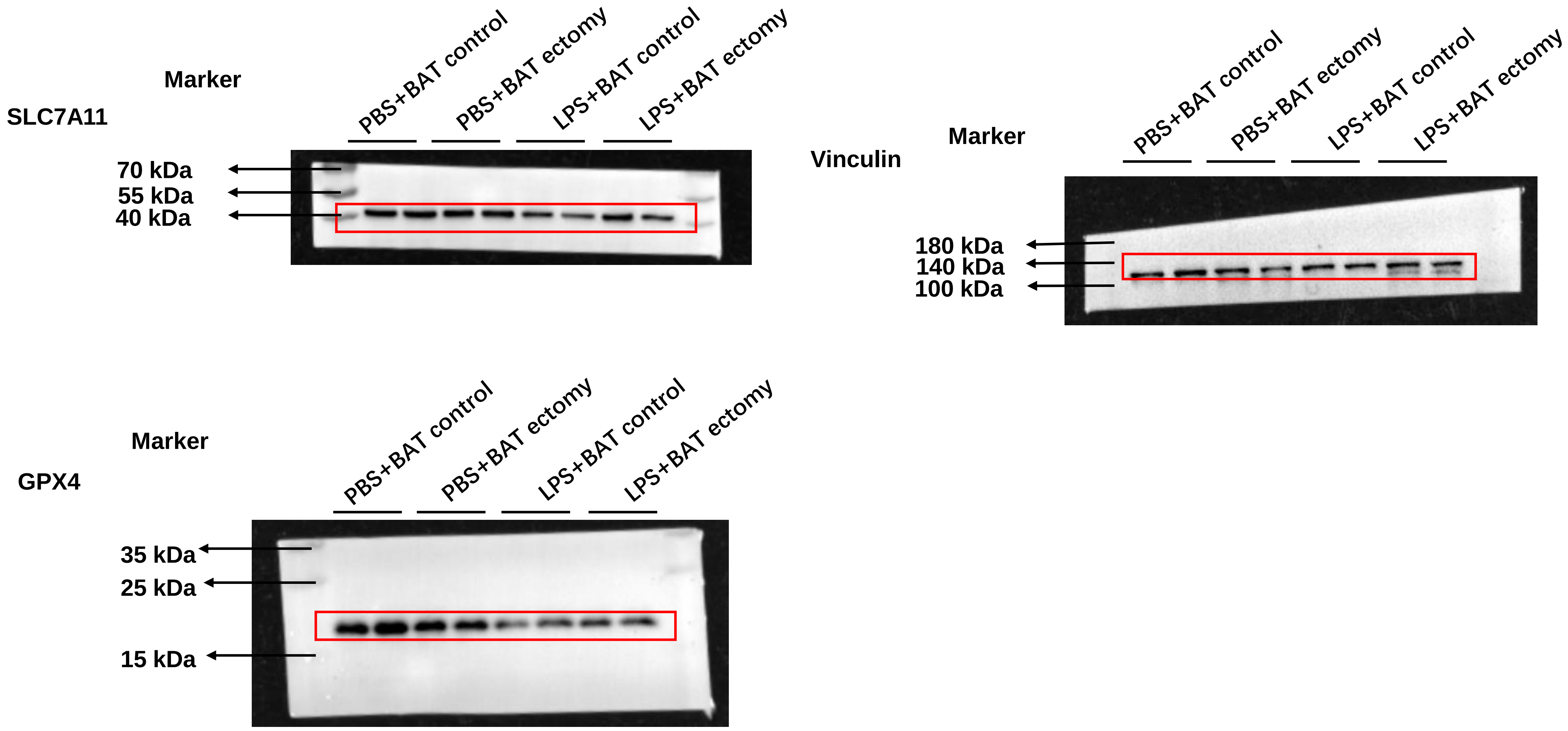
SLC7A11
Marker
PBS+BAT control
PBS+BAT ectomy
LPS+BAT control
LPS+BAT ectomy
70 kDa
55 kDa
40 kDa
Vinculin
Marker
PBS+BAT control
PBS+BAT ectomy
LPS+BAT control
LPS+BAT ectomy
180 kDa
140 kDa
100 kDa
GPX4
Marker
PBS+BAT control
PBS+BAT ectomy
LPS+BAT control
LPS+BAT ectomy
35 kDa
25 kDa
15 kDa

Figure 8f

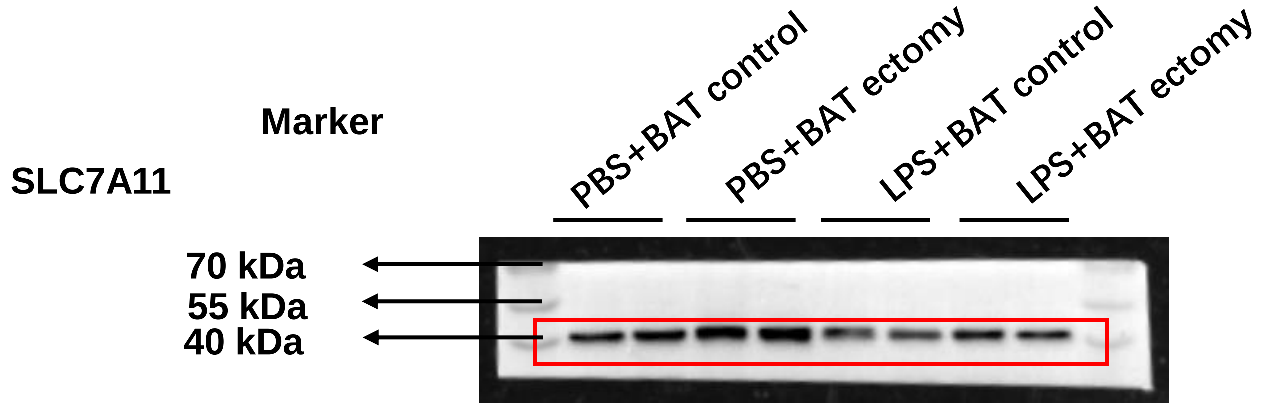
SLC7A11
Marker
PBS+BAT control
PBS+BAT ectomy
LPS+BAT control
LPS+BAT ectomy
70 kDa
55 kDa
40 kDa

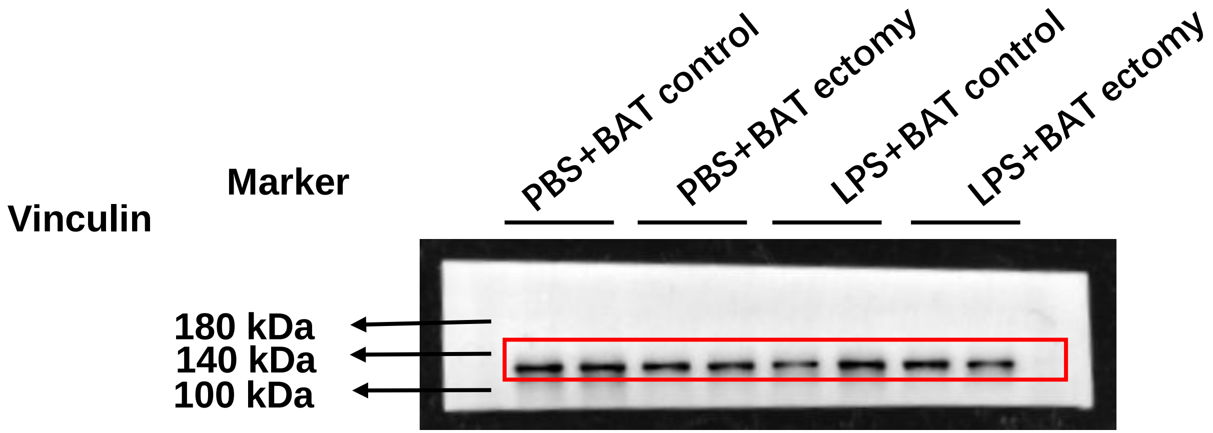
Vinculin
Marker
PBS+BAT control
PBS+BAT ectomy
LPS+BAT control
LPS+BAT ectomy
180 kDa
140 kDa
100 kDa

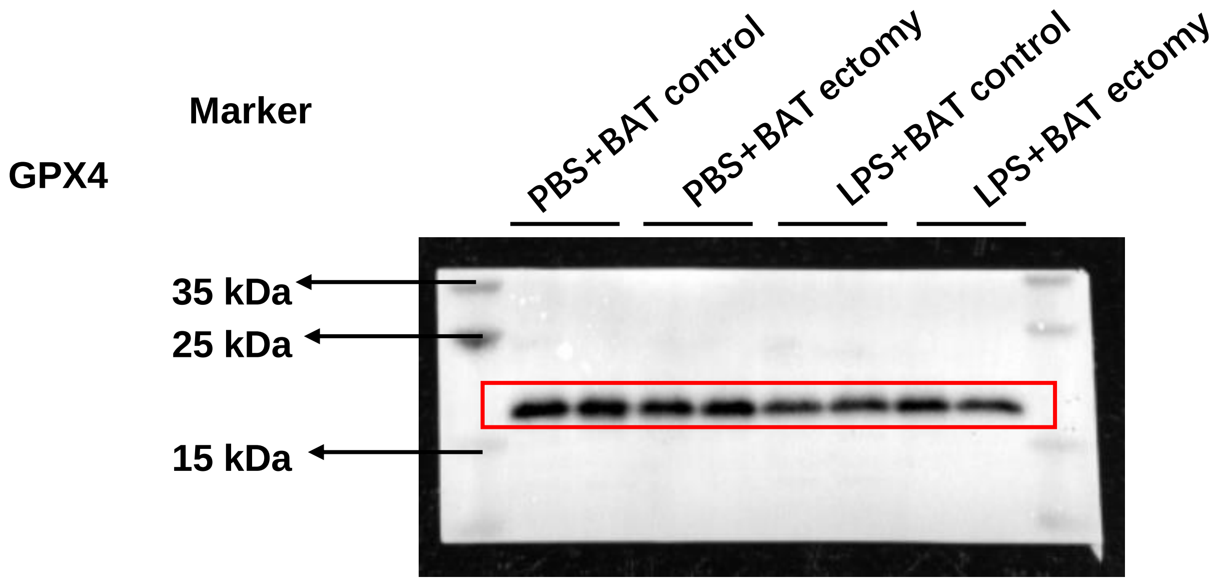
GPX4
Marker
PBS+BAT control
PBS+BAT ectomy
LPS+BAT control
LPS+BAT ectomy
35 kDa
25 kDa
15 kDa

Figure 8i

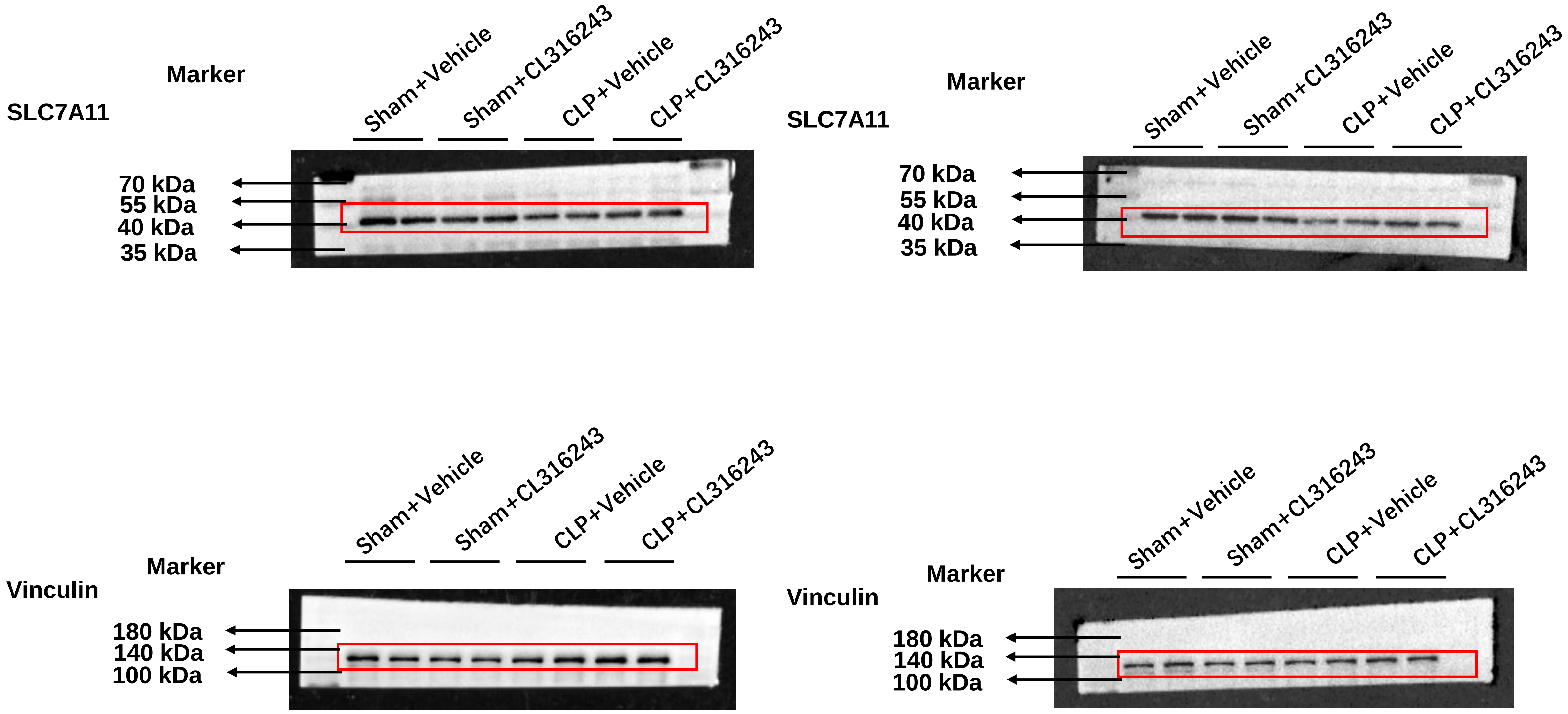
SLC7A11
Marker
Sham+Vehicle
Sham+CL316243
CLP+Vehicle
CLP+CL316243
70 kDa
55 kDa
40 kDa
35 kDa
SLC7A11
Marker
Sham+Vehicle
Sham+CL316243
CLP+Vehicle
CLP+CL316243
70 kDa
55 kDa
40 kDa
35 kDa
Vinculin
Marker
Sham+Vehicle
Sham+CL316243
CLP+Vehicle
CLP+CL316243
180 kDa
140 kDa
100 kDa
Vinculin
Marker
Sham+Vehicle
Sham+CL316243
CLP+Vehicle
CLP+CL316243
180 kDa
140 kDa
100 kDa

Figure 8i

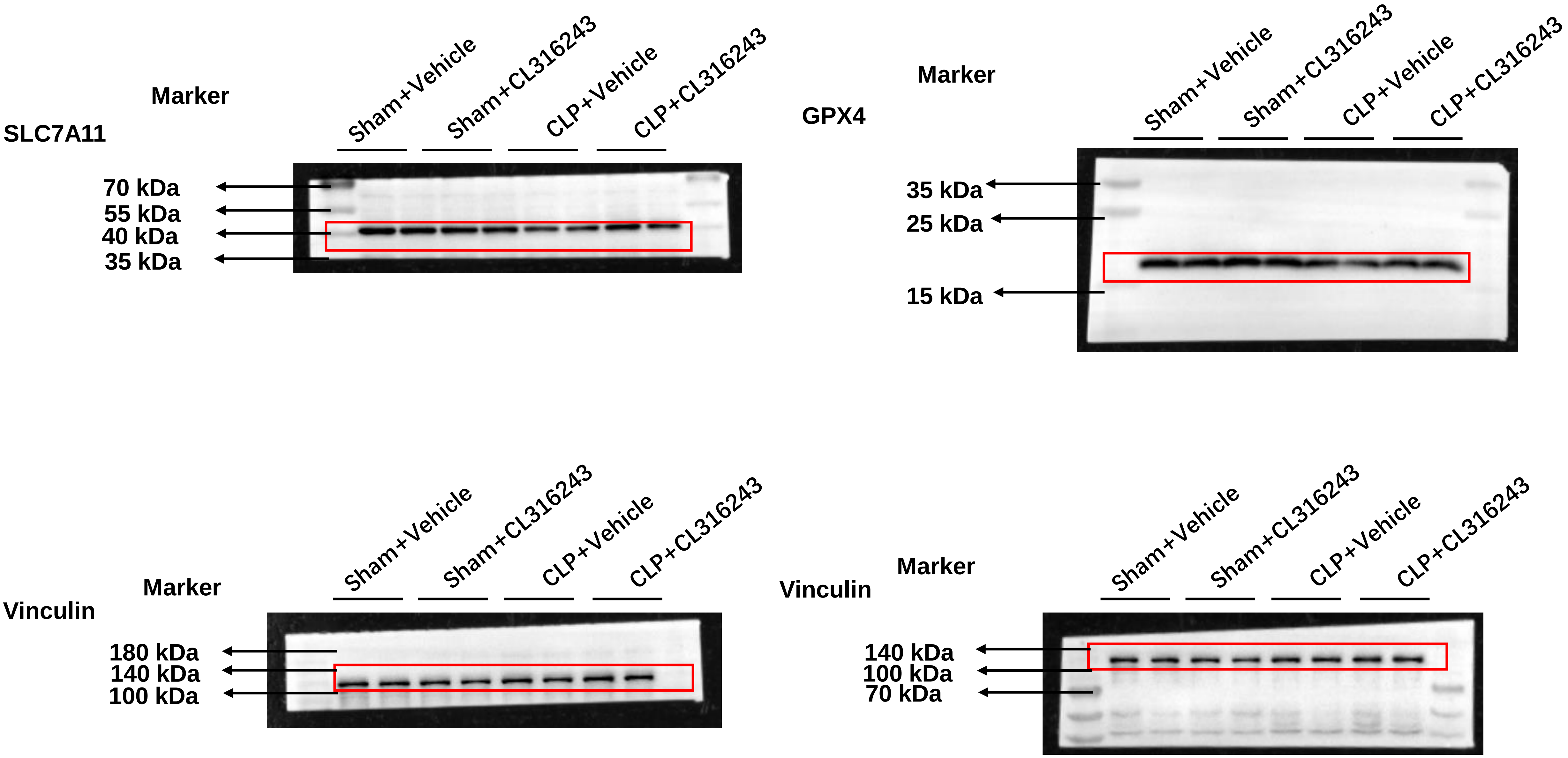
SLC7A11
Marker
Sham+Vehicle
Sham+CL316243
CLP+Vehicle
CLP+CL316243
70 kDa
55 kDa
40 kDa
35 kDa
GPX4
Marker
Sham+Vehicle
Sham+CL316243
CLP+Vehicle
CLP+CL316243
35 kDa
25 kDa
15 kDa
Vinculin
Marker
Sham+Vehicle
Sham+CL316243
CLP+Vehicle
CLP+CL316243
180 kDa
140 kDa
100 kDa
Vinculin
Marker
Sham+Vehicle
Sham+CL316243
CLP+Vehicle
CLP+CL316243
140 kDa
100 kDa
70 kDa

Figure 8i

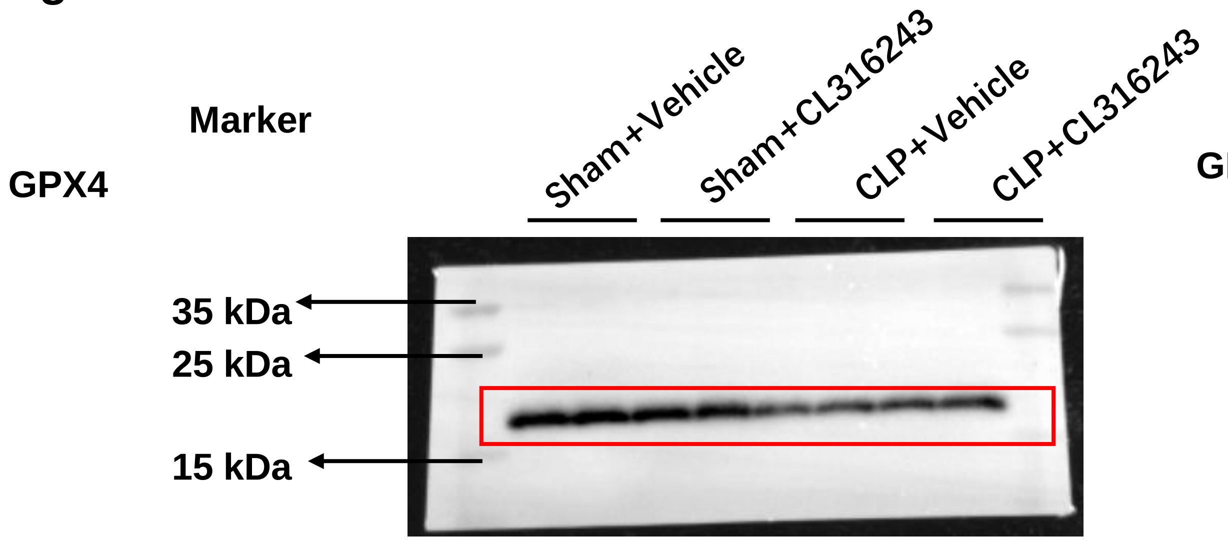

Marker
GPX4
Sham+Vehicle
Sham+CL316243
CLP+Vehicle
CLP+CL316243
35 kDa
25 kDa
15 kDa

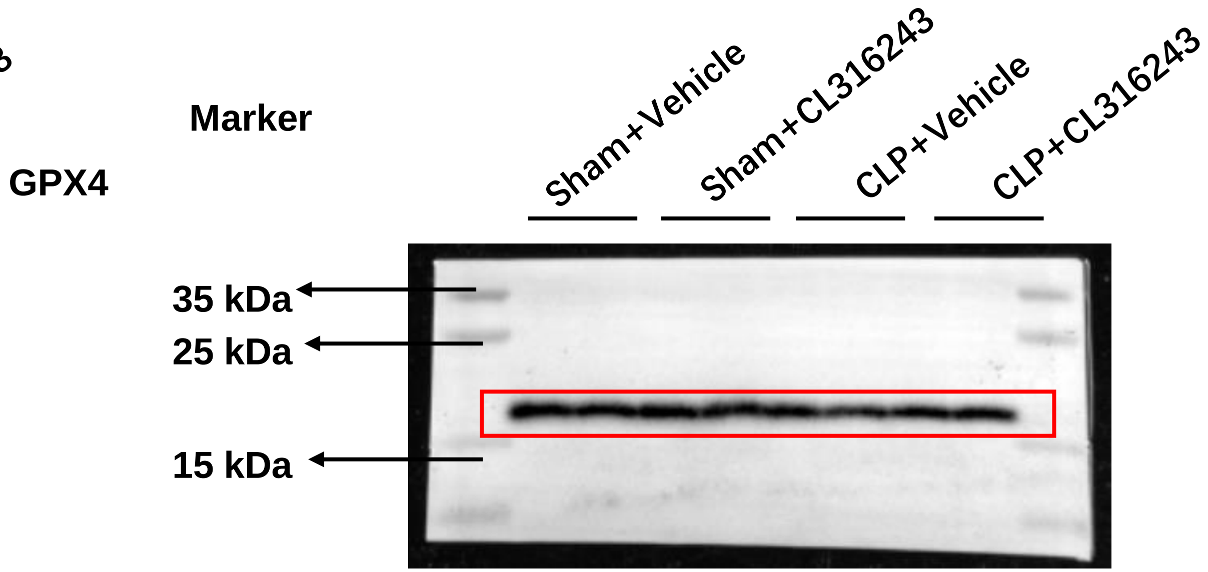

Marker
GPX4
Sham+Vehicle
Sham+CL316243
CLP+Vehicle
CLP+CL316243
35 kDa
25 kDa
15 kDa

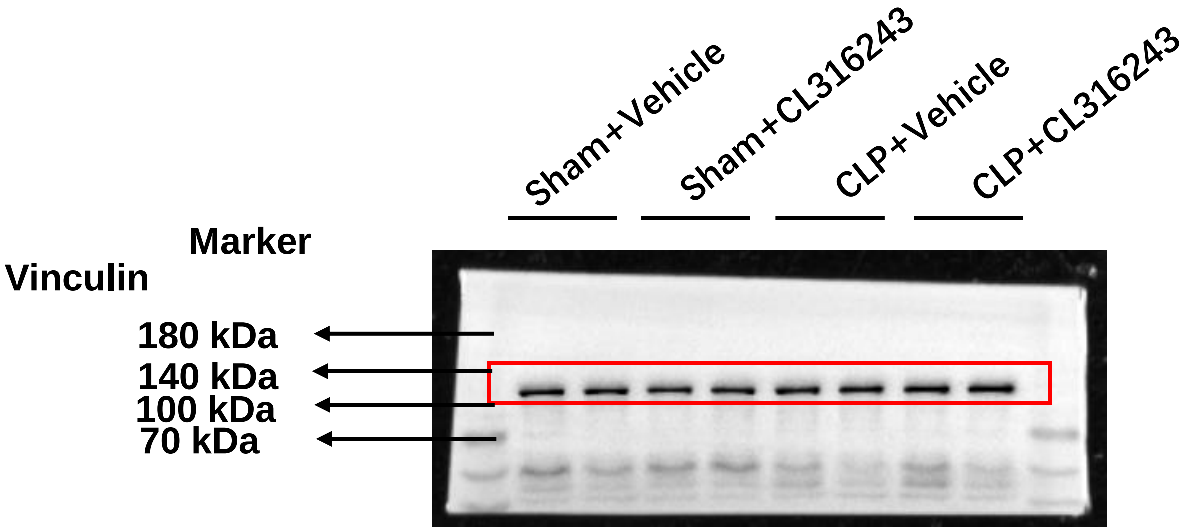

Marker
Vinculin
Sham+Vehicle
Sham+CL316243
CLP+Vehicle
CLP+CL316243
180 kDa
140 kDa
100 kDa
70 kDa

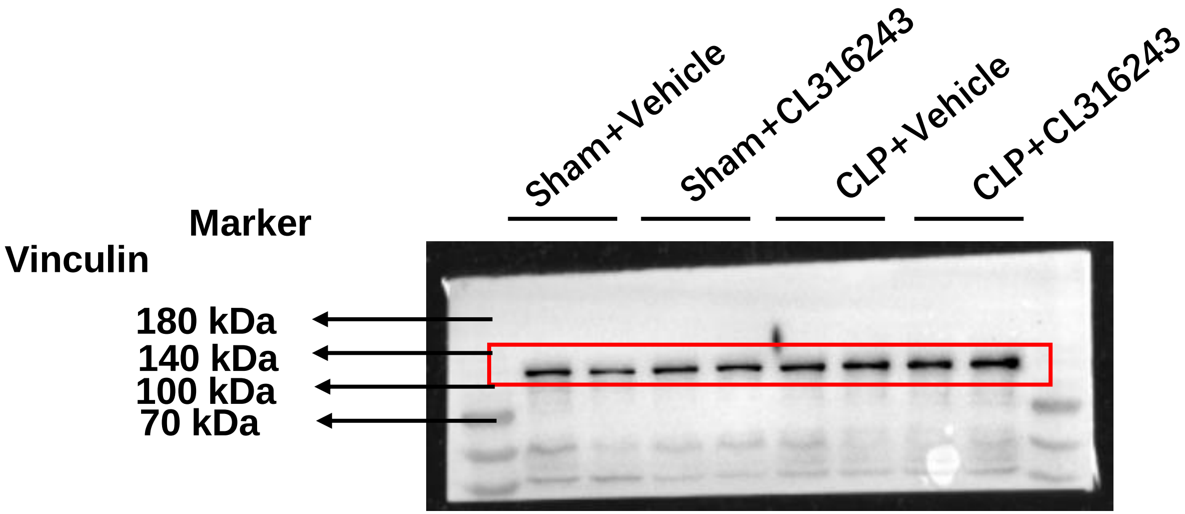

Marker
Vinculin
Sham+Vehicle
Sham+CL316243
CLP+Vehicle
CLP+CL316243
180 kDa
140 kDa
100 kDa
70 kDa

Figure 8I

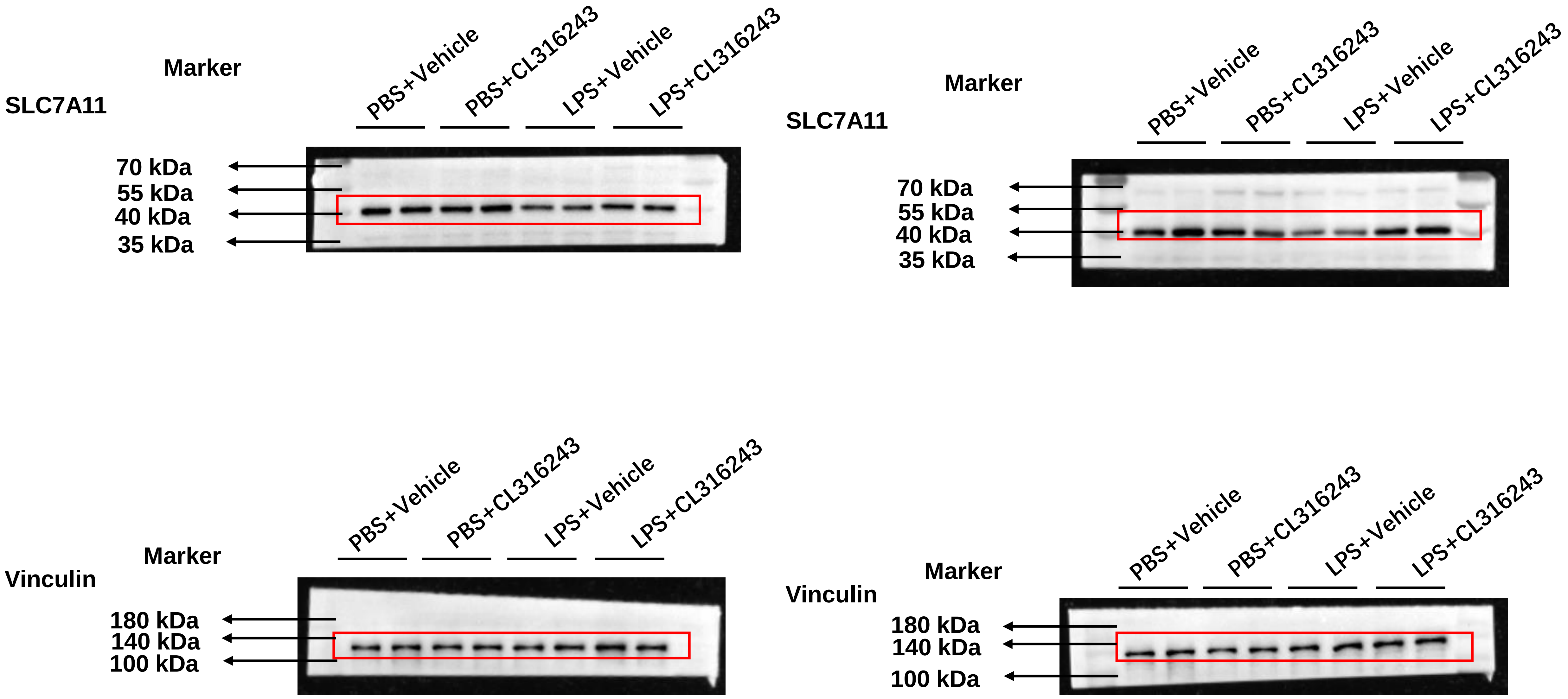
SLC7A11
Marker
PBS+Vehicle
PBS+CL316243
LPS+Vehicle
LPS+CL316243
70 kDa
55 kDa
40 kDa
35 kDa
SLC7A11
Marker
PBS+Vehicle
PBS+CL316243
LPS+Vehicle
LPS+CL316243
70 kDa
55 kDa
40 kDa
35 kDa
Vinculin
Marker
PBS+Vehicle
PBS+CL316243
LPS+Vehicle
LPS+CL316243
180 kDa
140 kDa
100 kDa
Vinculin
Marker
PBS+Vehicle
PBS+CL316243
LPS+Vehicle
LPS+CL316243
180 kDa
140 kDa
100 kDa

Figure 8l

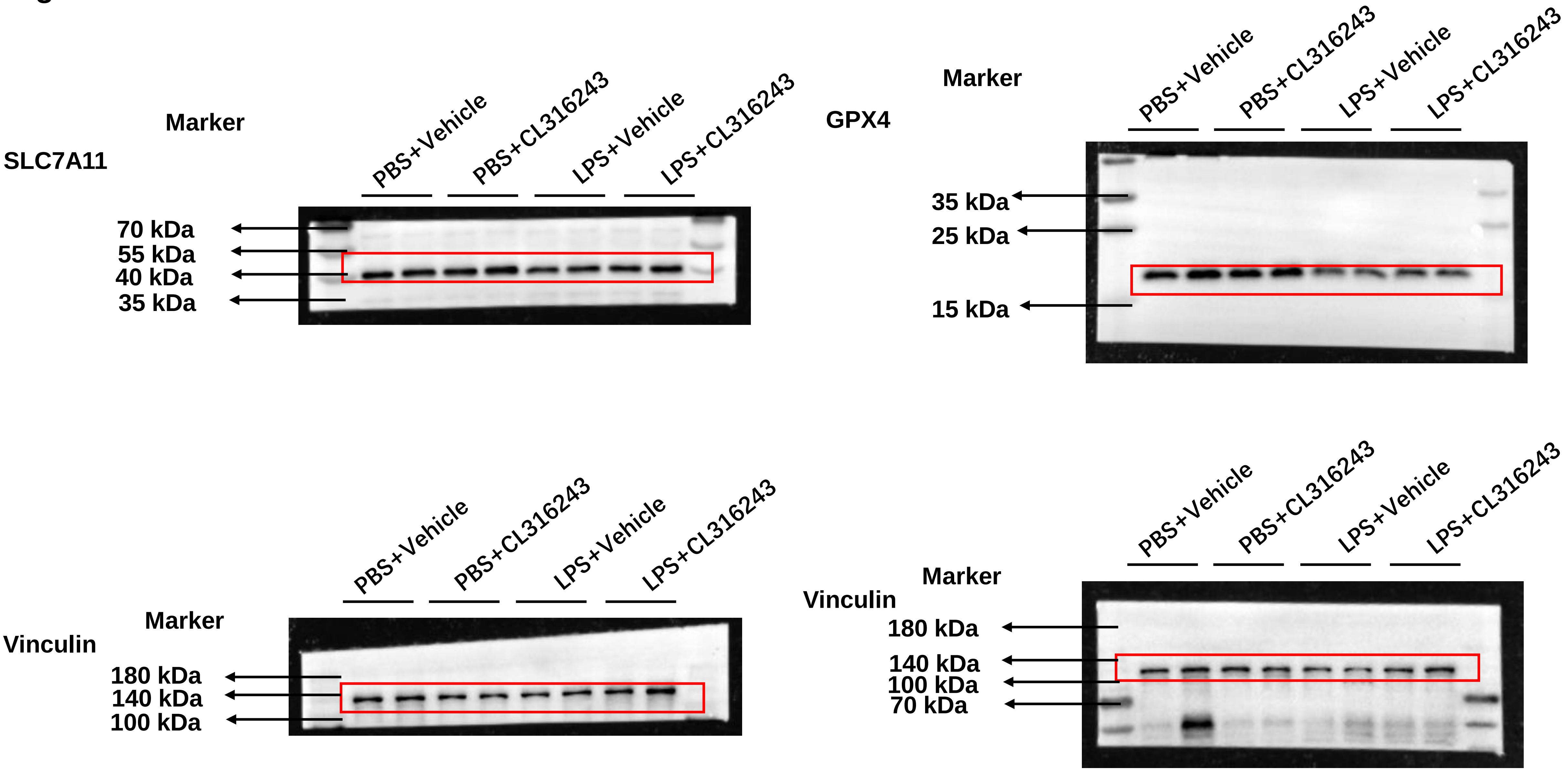

SLC7A11
Marker
PBS+Vehicle
PBS+CL316243
LPS+Vehicle
LPS+CL316243
70 kDa
55 kDa
40 kDa
35 kDa
GPX4
Marker
PBS+Vehicle
PBS+CL316243
LPS+Vehicle
LPS+CL316243
35 kDa
25 kDa
15 kDa
Vinculin
Marker
PBS+Vehicle
PBS+CL316243
LPS+Vehicle
LPS+CL316243
180 kDa
140 kDa
100 kDa
Vinculin
Marker
PBS+Vehicle
PBS+CL316243
LPS+Vehicle
LPS+CL316243
180 kDa
140 kDa
100 kDa
70 kDa

Figure 8I

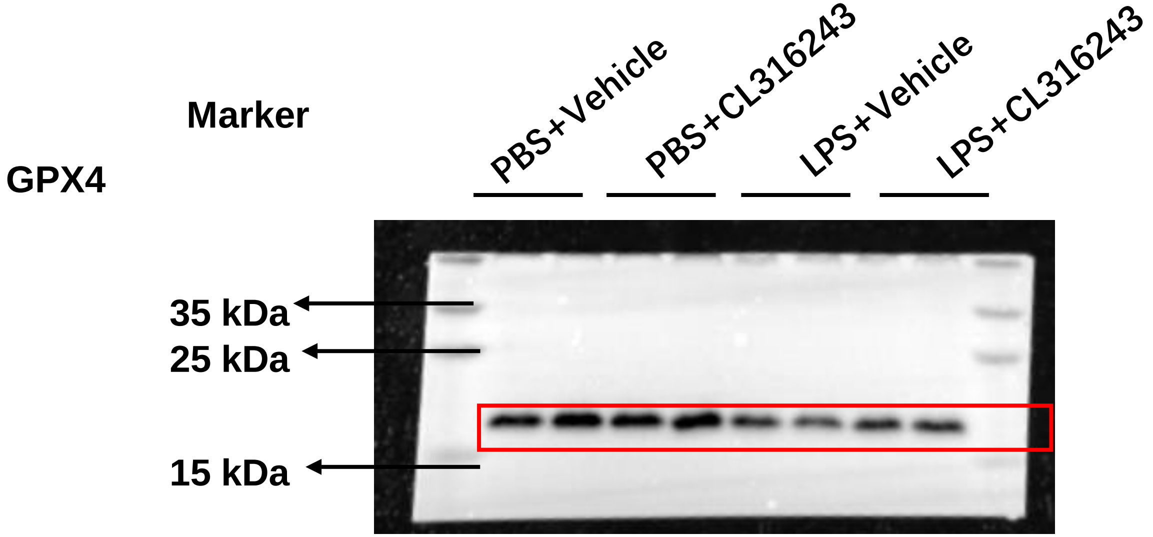

GPX4
Marker
PBS+Vehicle
PBS+CL316243
LPS+Vehicle
LPS+CL316243
35 kDa
25 kDa
15 kDa

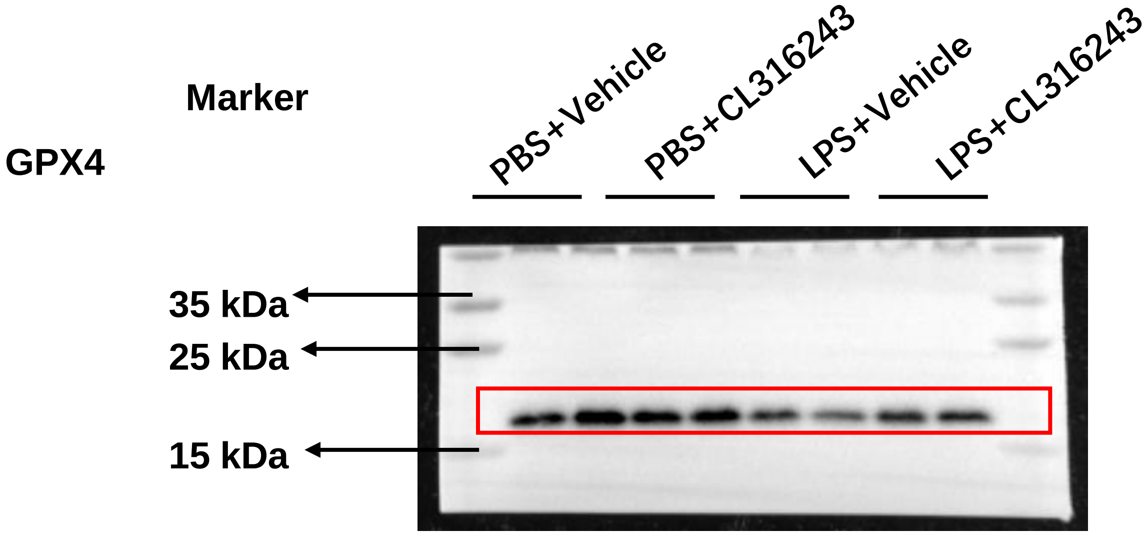

GPX4
Marker
PBS+Vehicle
PBS+CL316243
LPS+Vehicle
LPS+CL316243
35 kDa
25 kDa
15 kDa

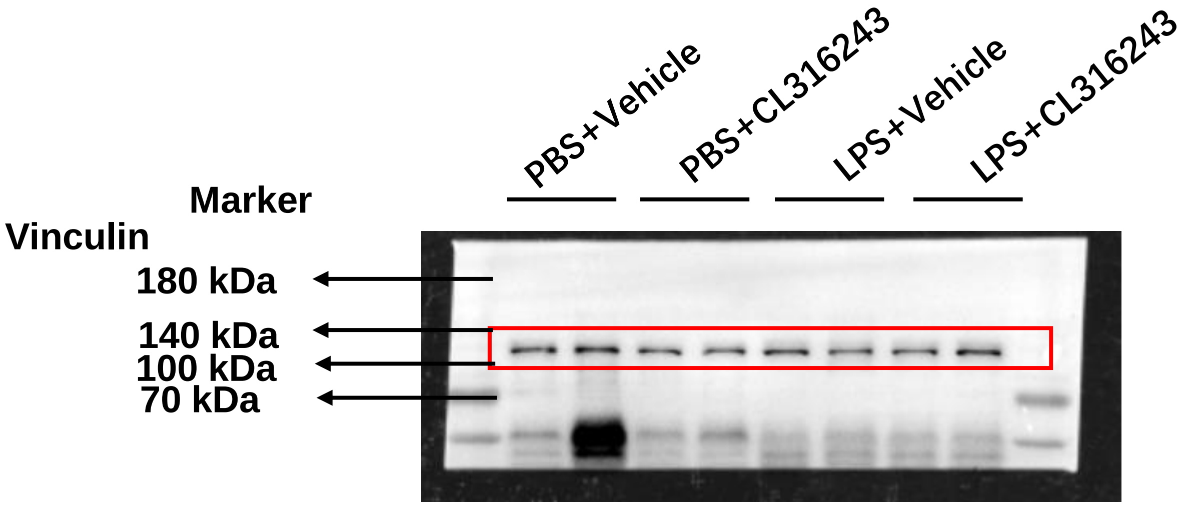

Vinculin
Marker
PBS+Vehicle
PBS+CL316243
LPS+Vehicle
LPS+CL316243
180 kDa
140 kDa
100 kDa
70 kDa

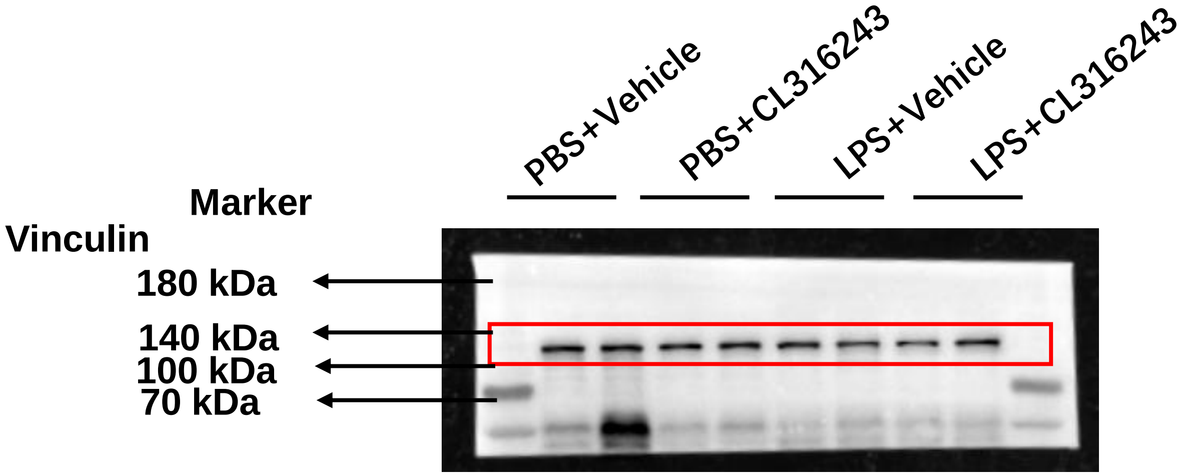

Vinculin
Marker
PBS+Vehicle
PBS+CL316243
LPS+Vehicle
LPS+CL316243
180 kDa
140 kDa
100 kDa
70 kDa
